# The foxtail millet (*Setaria italica*) terpene synthase gene family

**DOI:** 10.1111/tpj.14771

**Published:** 2020-05-03

**Authors:** Prema S. Karunanithi, David I. Berrios, Sadira Wang, John Davis, Tong Shen, Oliver Fiehn, Julin N. Maloof, Philipp Zerbe

**Affiliations:** ^1^ Department of Plant Biology University of California–Davis One Shields Avenue Davis 95616 CA USA; ^2^ West Coast Metabolomics Center University of California–Davis One Shields Avenue Davis 95616 CA USA

**Keywords:** crop stress resilience, natural products, pathway discovery, plant specialized metabolism, *Setaria italica*, terpene synthases

## Abstract

Terpenoid metabolism plays vital roles in stress defense and the environmental adaptation of monocot crops. Here, we describe the identification of the terpene synthase (TPS) gene family of the panicoid food and bioenergy model crop foxtail millet (*Setaria italica*). The diploid *S. italica* genome contains 32 TPS genes, 17 of which were biochemically characterized in this study. Unlike other thus far investigated grasses, *S. italica* contains TPSs producing all three *ent*‐, (+)‐ and *syn*‐copalyl pyrophosphate stereoisomers that naturally occur as central building blocks in the biosynthesis of distinct monocot diterpenoids. Conversion of these intermediates by the promiscuous TPS SiTPS8 yielded different diterpenoid scaffolds. Additionally, a cytochrome P450 monooxygenase (CYP99A17), which genomically clustered with SiTPS8, catalyzes the C19 hydroxylation of SiTPS8 products to generate the corresponding diterpene alcohols. The presence of syntenic orthologs to about 19% of the *S. italica* TPSs in related grasses supports a common ancestry of selected pathway branches. Among the identified enzyme products, abietadien‐19‐ol, *syn*‐pimara‐7,15‐dien‐19‐ol and germacrene‐d‐4‐ol were detectable *in planta*, and gene expression analysis of the biosynthetic TPSs showed distinct and, albeit moderately, inducible expression patterns in response to biotic and abiotic stress. *In vitro* growth‐inhibiting activity of abietadien‐19‐ol and *syn*‐pimara‐7,15‐dien‐19‐ol against *Fusarium verticillioides* and *Fusarium subglutinans* may indicate pathogen defensive functions, whereas the low antifungal efficacy of tested sesquiterpenoids supports other bioactivities. Together, these findings expand the known chemical space of monocot terpenoid metabolism to enable further investigations of terpenoid‐mediated stress resilience in these agriculturally important species.

## Significance Statement

The diverse class of terpenoids serves important functions in inter‐organismal and environmental interactions in many plant species. The discovery of terpenoid metabolic pathways in the model crop foxtail millet (*Setaria italica*) provides foundational knowledge of the diversity of terpenoid metabolism in poaceous food and bioenergy crops for possible future application of these natural defense mechanisms for improving crop traits.

## INTRODUCTION

Foxtail millet (*Setaria italica*) is a grain crop of the grass family (Panicoideae; Poaceae) that was domesticated from its wild relative green foxtail (*Setaria viridis*) in northern China over 8700 years ago (Lu *et al.*, 2009). Valued for their nutritional benefits, climate resilience and low‐input cultivation, foxtail millet and related small millets are cultivated in many arid and semi‐arid regions of Asia, southern Europe, India, South America and North Africa (Goron and Raizada, 2015; Pant *et al.*, 2016). In addition, their relatively short lifecycle and small diploid genomes (about 500 Mb) have made *S. italica* and *S. viridis* well‐suited as model species for related food and bioenergy crops (Doust *et al.*, 2009; Bennetzen *et al.*, 2012; Huang *et al.*, 2016).

While the molecular mechanisms underlying environmental resilience in millets have remained largely unknown, related monocot crops, especially rice (*Oryza sativa*) and maize (*Zea mays*), deploy species‐specific mixtures of terpenoid metabolites as core modulators of both biotic and abiotic stress defenses (Schmelz *et al.*, 2014; Murphy and Zerbe, 2020). Rice accumulates distinct groups of stress‐inducible diterpenoid phytoalexins with potent antimicrobial efficacy against major rice pathogens such as rice blast (*Magnaporthe oryza*) (Xu *et al.*, 2012; Bagnaresi *et al.*, 2012; Han *et al.*, 2015; Lu *et al.*, 2018). Among these bioactive diterpenoids, momilactones further demonstrate allelopathic functions (Kato‐Noguchi and Peters, 2013; Toyomasu *et al.*, 2014). Species‐specific arrays of stress‐elicited volatile sesquiterpenoids and non‐volatile diterpenoids have also been identified in maize and shown to confer substantial resilience to various fungal pathogens, including species of *Fusarium*, *Aspergillus* and *Colletotrichum*, as well as against herbivore pests such as the European corn borer (*Ostrinia nubilalis*) and lepidopteran larvae (Harris *et al.*, 2005; Schnee *et al.*, 2006; Köllner *et al.*, 2008; Dafoe *et al.*, 2011; Huffaker *et al.*, 2011; Schmelz *et al.*, 2011; Köllner *et al.*, 2013; Ding *et al.*, 2017; Mafu *et al.*, 2018; Ding *et al.*, 2019). Beyond the functions of terpenoids in biotic stress responses, the accumulation of terpenoids in response to abiotic stressors has been reported in maize and rice (Kodama *et al.*, 1988; Horie *et al.*, 2015; Vaughan *et al.*, 2015; Mafu *et al.*, 2018). In addition, terpenoid‐deficient maize mutants show increased drought susceptibility, and high CO_2_ levels have a negative impact on terpenoid production and stress resistance, supporting the importance of terpenoids also in abiotic stress tolerance (Vaughan *et al.*, 2014; Christensen *et al.*, 2018).

Despite their structural diversity, all terpenoids are derived from only a few acyclic prenyl pyrophosphate precursors that differ by the number of condensed five‐carbon isoprenoid units (C10, C15, C20, etc.) (Chen *et al.*, 2011). Downstream of this central precursor pool, the vast chemical space of species‐specific terpenoids is largely determined by terpene synthases (TPSs), cytochrome P450 monooxygenases (P450s) and other modifying enzyme classes (Banerjee and Hamberger, 2018; Karunanithi and Zerbe, 2019). Most commonly, TPSs catalyze the cyclization and rearrangement of their respective prenyl pyrophosphate substrates to generate a range of mono‐ (C10), sesqui‐ (C15) and di‐ (C20) terpenoid scaffolds (Chen *et al.*, 2011). The majority of these enzymes represent class I TPSs that initiate substrate conversion through cleavage of the pyrophosphate leaving group. Uniquely, in angiosperms the formation of labdane diterpene scaffolds, which include kauranes, pimaranes, abietanes and related terpene groups, recruits pairs of class II and class I diTPSs that function sequentially to generate distinct scaffolds (Peters, 2010; Karunanithi and Zerbe, 2019). Here, the central diterpenoid precursor, geranylgeranyl pyrophosphate (GGPP), first undergoes protonation‐dependent cyclization by a class II diTPS to generate bicyclic prenyl pyrophosphate intermediates of different normal (+)‐, *ent*‐ or *syn*‐stereochemistry. Ionization‐dependent cyclization and rearrangement of the resulting intermediate by class I diTPSs then yields an array of distinct labdane scaffolds. Functional decoration of these terpene building blocks, typically initiated by oxygenation through the activity of cytochrome P450 monooxygenases (P450), ultimately generates the broad structural and functional diversity of plant terpenoids (Banerjee and Hamberger, 2018).

Enabled by the increasing availability of genomic resources, the TPS families and selected terpenoid metabolic members of the vast P450 families have been identified in several crop species of the Panicoideae, including rice, maize, wheat (*Triticum aestivum*) and switchgrass (*Panicum virgatum*) (reviewed in Schmelz *et al.*, 2014; Murphy and Zerbe, 2020). Functional enzyme characterization has provided detailed insights into the expansion of the TPS and P450 families through repeated duplication and functional diversification of ancestral gibberellin (GA) biosynthetic genes that forms the basis for the evolution of species‐specific terpenoid networks as critical components of monocot stress resilience (Schmelz *et al.*, 2014; Zi *et al.*, 2014).

To deepen our understanding of the diversity of terpenoid metabolism in monocot crops, this study describes the genome‐wide discovery of the *S. italica* TPS family. Biochemical characterization of 17 TPSs and a diterpenoid‐metabolic P450 (CYP99A17) demonstrates the presence of both common and species‐specific terpenoid pathways. Terpenoid bioactivity assays combined with patterns of stress‐elicited terpenoid biosynthesis suggest possible roles for *S. italica* terpenoid metabolism in both biotic and abiotic stress responses.

## RESULTS

### Identification of TPS candidates

**Figure 1:**
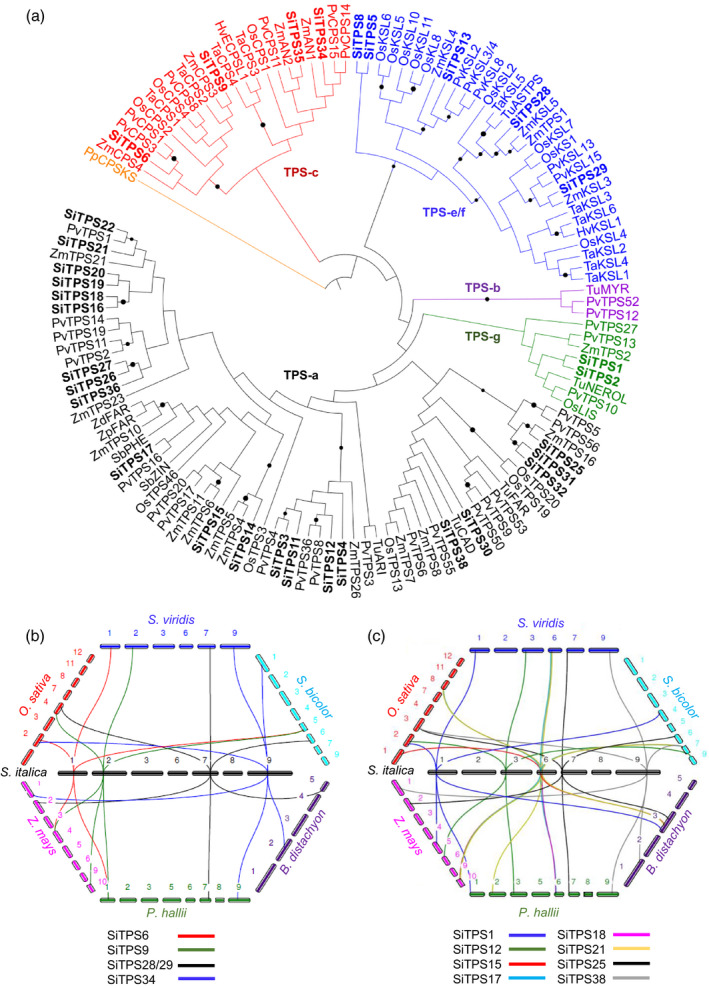
Phylogenetic and gene synteny analysis of *Setaria italica* terpene synthases (TPSs). (a) Maximum likelihood phylogeny of *S. italica* TPSs (bold) and selected TPS‐c, TPS‐e/f, TPS‐g and TPS‐a2 clade TPSs from related poaceous crop species. Tree rooted with the *ent*‐CPP/*ent*‐kaurene synthase from Physcomitrella* patens*, PpCPS/KS. Black circles denote bootstrap support of > 80% (1000 repetitions). Accession numbers and protein sequences are given in Table S1. (b), (c) Synteny and orthology of diterpene synthases (b) and sesquiterpene synthases (c) between *S. italica*, *Setaria viridis* and related diploid poaceous grasses. All chromosomes for *Zea mays* (magenta), *Panicum hallii* (green), *Brachypodium distachyon* (purple), *Sorghum bicolor* (cyan), *S. viridis* (blue) and *Oryza sativa* (red) are arranged in a polygon to emphasize synteny with *S. italica* (black). Chromosome IDs are given on each line segment. A syntenic network is depicted as a colored line specific to a TPS gene located on *S. italica* chromosomes (black) connecting to respective syntenic orthologs on chromosomes of other genomes (Table S3).

To identify the *S. italica* TPS gene family*,* we mined publicly available genome data (v.2.2; https://phytozome.jgi.doe.gov) using BLAST analysis against a curated protein database of known plant TPSs (Zerbe *et al.*, 2013). A total of 39 genes with best matches to known TPSs were identified, of which 32 represented full‐length sequences (Table S1 in the online Supporting Information). Most TPS genes mapped to *S. italica* chromosomes 1, 6 and 7, whereas no TPS genes were located on chromosomes 4 and 5 (Table S2). Next, analysis of signature sequence motifs and phylogenetic comparison with TPSs from related monocot crops were used to functionally categorize the identified gene candidates. The presence of the catalytic DxDD motif and lack of a DDxxD motif characteristic for class I catalysis, combined with a close phylogenetic relationship to known TPSs of the TPS‐c clade, supported class II diTPS activity for SiTPS6, SiTPS9, SiTPS34 and SiTPS35 (Figures 1a, S1 and S2). Specifically, SiTPS34 and SiTPS35 clustered most closely with characterized *ent*‐copalyl pyrophosphate (CPP) synthases of general metabolism (e.g. *Z. mays* ANTHER EAR 1, ZmAn1) or specialized metabolism (e.g. *Z. mays* ANTHER EAR 2, ZmAn2), respectively, and contained the catalytic His–Asn dyad conserved among *ent*‐CPP synthases involved in GA biosynthesis (Harris *et al.*, 2005; Köksal *et al.*, 2014; Potter *et al.*, 2014; Lemke *et al.*, 2019), suggesting related functionalities (Figures 1a and S1). By contrast, SiTPS6 and SiTPS9 lacked the His–Asn motif, and phylogenetic analysis placed both enzymes with class II diTPSs of specialized metabolism, such as the maize 8,13‐CPP synthase ZmCPS4 and the wheat (+)‐CPP synthase TaCPS2 (Wu *et al.*, 2012; Murphy *et al.*, 2018) (Figures 1a and S1). The remaining 28 TPS candidates were designated as putative class I TPSs on account of the presence of only the conserved C‐terminal DDxxD motif (Figure S2). Of these TPSs, five clustered with diTPSs of the TPS‐e/f clade (Figure 1a). A close phylogenetic relationship of SiTPS28 and SiTPS29 to ZmTPS1, ZmKSL3 and ZmKSL5 indicated possible *ent‐*kaurene synthase activity for the encoded enzymes. This is further supported by the presence of a catalytic isoleucine residue previously shown to be conserved among *ent*‐kaurene synthases (Xu *et al.*, 2007a; Jia and Peters, 2016) (Figure S2). By contrast, SiTPS5, SiTPS8 and SiTPS13 were most closely related to class I diTPSs of known functions in specialized metabolism (Figure 1a). Notably, SiTPS5 and SiTPS8 clustered on a separate branch distant from other specialized diTPSs. In addition, SiTPS8 was the only *S. italica* diTPS candidate featuring the βα‐bi‐domain architecture more commonly present in mono‐ and sesqui‐TPSs. Similar bi‐domain diTPS have also been discovered in a range of other plant species including wheat and switchgrass (Hillwig *et al.*, 2011; Zhou *et al.*, 2012; Pelot *et al.*, 2018). However, SiTPS8 does not phylogenetically cluster with the respective bi‐domain diTPSs from wheat (TaKSL5; Hillwig *et al* *.*, 2011) and switchgrass (PvKS3/4; Pelot *et al* *.*, 2018) (Figure 1a). Outside the TPS‐e/f clade, SiTPS1 and SiTPS2 were placed with members of the TPS‐g clade that predominantly contains class I TPSs producing acyclic terpenes (Gao *et al.*, 2018). The remaining 23 class I TPS candidates were assigned to the TPS‐a2 clade comprising monocot sesqui‐TPSs (Figure 1a). Putative functions of these TPSs in sesquiterpenoid metabolism were further supported by the absence of the N‐terminal RRx_8_W motif characteristic of mono‐TPSs and the lack of plastidial transit peptides in at least 19 of the identified TPS candidates. Close phylogenetic relationships of several *S. italica* TPS candidates with known sesqui‐TPSs from related monocot species, such as maize and switchgrass, suggested similar sesqui‐TPS activities (Schnee *et al.*, 2002; Köllner *et al.*, 2008a; Köllner *et al.*, 2008b, Köllner *et al.*, 2009; Muchlinski *et al.*, 2019).

Having identified gene candidates for the *S. italica* TPS family, we next applied the same strategy to identify related TPS genes in the wild ancestor *S. viridis*. Consistent with the close evolutionary relationship between both species (Huang *et al.*, 2016), the *S. viridis* genome (v.1.1; https://phytozome.jgi.doe.gov) contains 34 putative full‐length TPS genes. High protein sequence identities of 86–100% between these genes and their respective *S. italica* homologs. with the majority of amino acid variation occurring in regions outside the predicted active site domains, suggested identical or closely related catalytic activities of the TPSs in these two species (Figure S3). To further examine the evolutionary interrelations of the *S. italica* and *S. viridis* TPS families with TPSs in closely related diploid poaceous species, including maize, rice, sorghum, *Panicum hallii* (diploid relative of the allotetraploid *Panicum virgatum*) and *Brachypodium distachyon,* gene synteny studies were conducted (Figure 1b,c, Table S3). Comparison of the syntenic orthologs across the respective genomes demonstrated that, with the exception of *SiTPS19*, *SiTPS26* and *SiTPS31*, all *S. italica* TPS loci had syntenic orthologs on *S. viridis* chromosomes. The degree of syntenic ortholog conservation for *S. italica* TPS candidates on other genomes varied, with 53% to *P. hallii*, 38% to *B. distachyon*, 59% to rice, 59% to sorghum and 50% to maize (Table S3). Of the 32 *S. italica* TPS candidates, 13 TPS syntenic networks were conserved across almost all genomes analyzed in this study (Figure 1b,c, Table S3). Of the five conserved *diTPS* syntenic networks, those originating from *S. italica* genes (*SiTPS29* and *SiTPS34*) were observed among the genomes of all species, whereas *SiTPS6* and *SiTPS9* were common to all but the *B. distachyon* genome and *SiTPS28* was common to all but the *O. sativa* genome (Figure 1b). Similarly, among the eight conserved *S. italica* class I TPS syntenic networks, *SiTPS1*, *SiTPS21*, *SiTPS25* and *SiTPS38* showed gene synteny across all investigated genomes, while *SiTPS12* and *SiTPS17* lacked syntenic orthologs only in the genome of *B. distachyon*, and *SiTPS15* and *SiTPS18* lacked syntenic orthologs only in the genome of *Sorghum bicolor* or *Z. mays*, respectively (Figure 1c). All 13 of these TPS syntenic networks were highly conserved among the genomes of *S. italica* and *S. viridis*, whereas 11 were conserved between the genomes of *S. italica* and *P. hallii*, consistent with their close evolutionary relatedness. Syntenic TPS networks on *S. italica* chromosomes 3, 6, 7 and 9 are mostly conserved on the respective homologous chromosomes 3, 6, 7 and 9 of *S. viridis* and *P. hallii*, consistent with the expected collinearity between genomes of closely related species (Table S3). Select syntenic network associations included genes of known function, including switchgrass *1,8‐cineole synthase* (*PvTPS3*, chromosome 3), *β‐bisabolene synthase* (*PvTPS17*, chromosome 6), and a *germacrene d‐synthase* (*PvTPS55*, chromosome 9) (Muchlinski *et al.*, 2019). Additionally, TPSs on *S. italica* chromosome 6 featured syntenic orthologs mapping to one chromosome in *S. viridis* (chromosome 6), *P. hallii* (chromosome 6), *B. distachyon* (chromosome 3), *S. bicolor* (chromosome 7) and *Z. mays* (chromosome 10), the latter including ZmTPS10 producing *α‐bergamotene* and *β‐farnesene* (Köllner *et al.*, 2009). In contrast, TPSs on *S. italica* chromosome 7 have syntenic orthologs mapping to one chromosome in *S. viridis* (chromosome 6), *S. bicolor* (chromosome 6) and *Z. mays* (chromosome 2), but different chromosomes in *P. hallii* (chromosomes 2 and 7), *B. distachyon* (chromosomes 3 and 5) and *O. sativa* (chromosomes 3, 4 and 8), indicating diverse chromosomal origins for evolutionary hotspots for TPS genes across the Poaceae (Table S3). Interestingly, *SiTPS34* (chromosome 9) with a predicted *ent*‐CPP synthase function, was placed in a syntenic network with known *ent‐*CPP synthases from rice (*OsCPS1*, chromosome 2), maize (*ZmAN1*, chromosome 1) and switchgrass (*PvCPS14/15*, chromosome 9) (Bensen *et al.*, 1995; Toyomasu *et al.*, 2015; Pelot *et al.*, 2018). Similarly, the predicted *ent‐* *kaurene synthases* *SiTPS28*/*29* (chromosome 7) clustered in a syntenic network with *ent*‐*kaurene synthases* from rice (*OsKS1*, chromosome 4), maize (*ZmKSL3*/*5*, chromosome 2) and switchgrass (*PvKSL13*/*15*, chromosome 7). Additionally, *S. italica* class II diTPS candidates potentially involved in specialized metabolism, such as *SiTPS6*, were in syntenic networks with the *ent*‐*CPP* synthase of specialized metabolism in rice (*OsCPS2*, chromosome 2) and 8,13‐*CPP* synthases from maize (*ZmCPS4*, chromosome 4) and switchgrass (*PvCPS3*, chromosome 1) (Prisic *et al.*, 2004; Murphy *et al.*, 2018; Pelot *et al.*, 2018). Likewise, *SiTPS9* was placed in a syntenic network with *ZmCPS4* and the maize (+)‐*CPP* *synthase* *ZmCPS3* (chromosome 10) (Murphy *et al.*, 2018), *PvCPS3* (Pelot *et al.*, 2018) and the *rice syn*‐*CPP synthase* OsCPS4 (chromosome4) (Xu *et al.*, 2004; Otomo *et al.*, 2004).

### Functional characterization of class II diTPSs from *S. italica*

**Figure 2:**
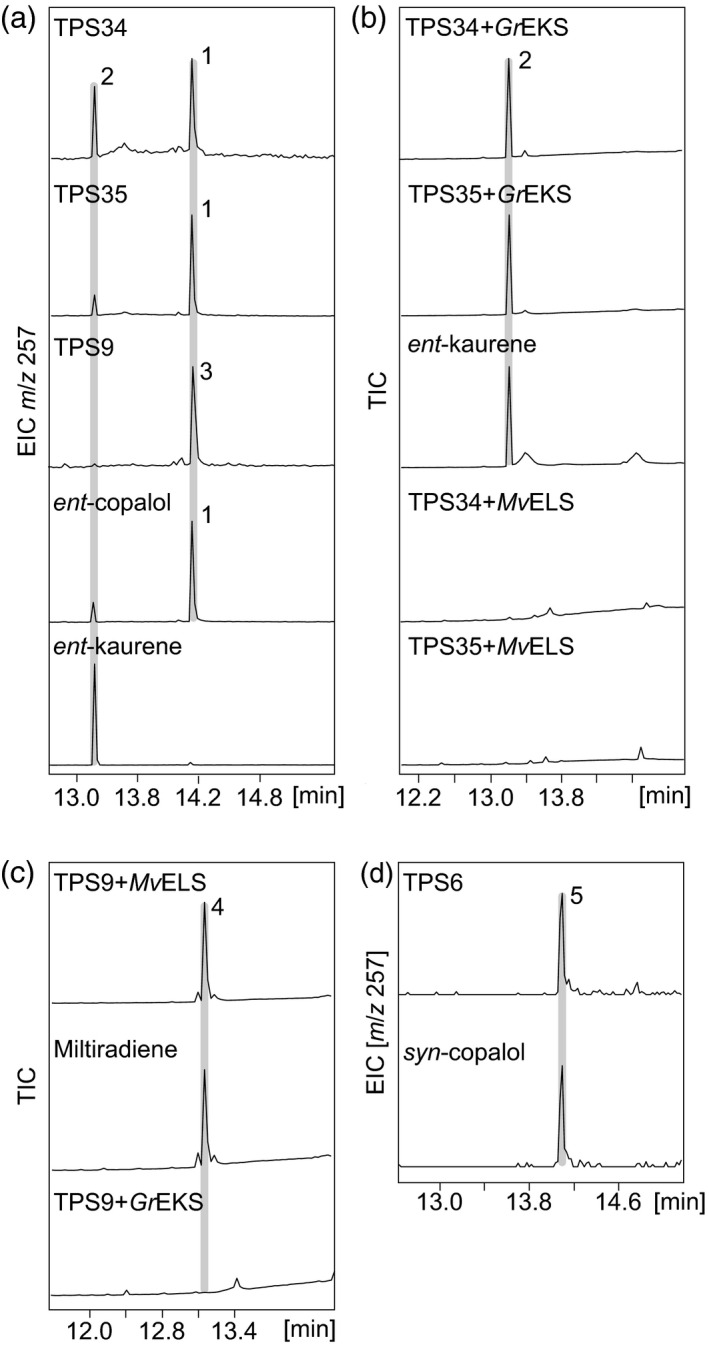
The GC‐MS analysis of *Setaria italica* class II diterpene synthase (diTPS) reaction products. (a) The GC‐MS extracted ion chromatograms (EIC, *m*/*z* 257) of reaction products from *Nicotiana benthamiana* co‐expression assays of the class II diTPS candidates SiTPS9, SiTPS34 and SiTPS35. Enzyme products were identified by comparison with the product of the known *ent*‐copalyl pyrophosphate synthase (CPS), ZmAn2 (Harris *et al.*, 2005). (b) The GC‐MS total ion chromatograms (TIC) of reaction products from *Nicotiana benthamiana* co‐expression assays of SiTPS34 and SiTPS35 with the *ent*‐kaurene synthase from *Grindelia robusta* (GrEKS) (Zerbe *et al.*, 2015) specific to converting *ent*‐copalyl pyrophosphate (*ent*‐CPP) into *ent*‐kaurene or a class I diTPS from *Marrubium vulgare* (MvELS) (Zerbe *et al.*, 2014) specific to converting (+)‐CPP into miltiradiene verified the stereochemistry of the SiTPS34 and SiTPS35 products as *ent*‐CPP. (c) The GC‐MS TICs of reaction products from *N. benthamiana* co‐expression assays of SiTPS9 with GrEKS or MvELS identify the SiTPS9 product as (+)‐CPP. (d) The GC‐MS EICs (*m*/*z* 257) of reaction products from *Escherichia coli* co‐expression assays of SiTPS6 compared with the product of the rice (*Oryza sativa*) *syn*‐CPP synthase, *Os*CPS4, identified the SiTPS6 product as *syn*‐CPP (Xu *et al.*, 2004). Note that all TPS products were analyzed in the form of their respective dephosphorylated derivatives to enable detection via GC‐MS analysis. Mass spectra for all compounds are given in Figure S2. 1, dephosphorylated *ent*‐CPP (*ent*‐copalol); 2, *ent*‐kaurene; 3, dephosphorylated (+)‐CPP ((+)‐copalol); 4, miltiradiene; 5, dephosphorylated *syn*‐CPP (*syn*‐copalol).

To biochemically analyze the predicted TPS activities we focused on the *S. italica* TPS family on account of the agricultural relevance of this species. Enzyme functional studies were conducted via *in vivo* combinatorial expression assays using *Nicotiana benthamiana* or *Escherichia coli* as platforms (Zerbe *et al.*, 2013; Kitaoka *et al.*, 2015a), dependent on the relative activity of individual TPS candidates in each host system. Consistent with their predicted activity as CPP synthases, expression of the class II diTPSs SiTPS9, SiTPS34 and SiTPS35 in *N. benthamiana* showed that these three enzymes produced CPP as compared to the authentic product of the maize *ent*‐CPP synthase, ZmAn2 (Harris *et al.*, 2005) (Figure 2a). To verify the stereochemistry of the respective CPP products, each enzyme was further co‐expressed with class I diTPSs specific to CPP of *ent*‐ or normal (+)‐stereochemistry, represented by an *ent*‐kaurene synthase from *Grindelia robusta* (GrEKS) (Zerbe *et al.*, 2013) converting *ent*‐CPP into *ent*‐kaurene and a class I diTPS from *Marrubium vulgare*, MvELS, that converts (+)‐CPP into miltiradiene (Zerbe *et al.*, 2014). This modular co‐expression approach revealed that SiTPS34 and SiTPS35 showed coupled activity only with GrEKS, identifying both enzymes as *ent*‐CPP synthases (Figure 2b). Conversely, SiTPS9 showed activity only in the pairwise reaction with MvELS, verifying the SiTPS9 product as (+)‐CPP (Figure 2c). Small quantities of *ent*‐kaurene observed in the product profiles of SiTPS34 and SiTPS35 probably result from the activity of endogenous *ent*‐kaurene synthases in *N. benthamiana* (Zerbe *et al.*, 2013)*.* Expression of SiTPS6 in *N. benthamiana* did not yield detectable products. However, the use of *E. coli* co‐expression assays revealed that SiTPS6 produced *syn*‐CPP as verified by comparison with the product of the rice *syn‐*CPP synthase, OsCPS4, and characteristic product mass ions of *m*/*z* 192 and 177 (Xu *et al.*, 2004) (Figure 2d). Mass spectra of enzyme products are given in Figure S4.

### Functional characterization of class I diTPSs from *S. italica*

**Figure 3:**
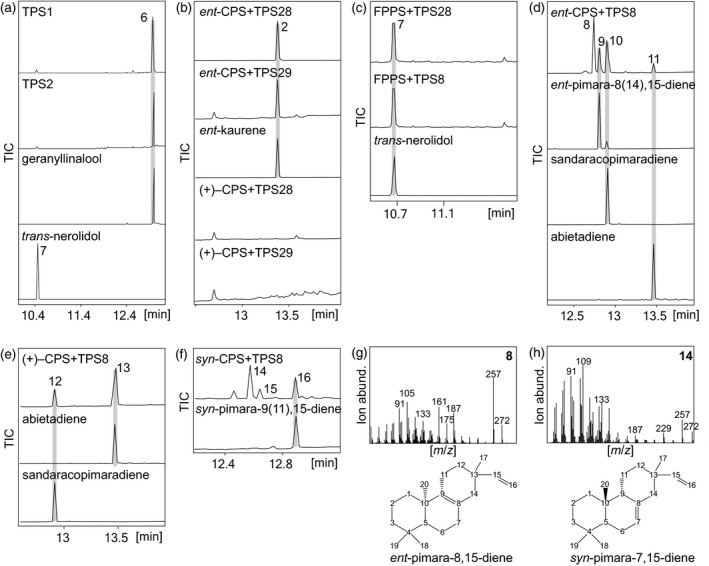
Functional characterization of the *Setaria italica* class I diterpene synthase (diTPS) candidates. (**a**) GC‐MS traces of products resulting from expression of SiTPS1 or SiTPS2 in *Nicotiana benthamiana* identifying both enzymes as geranyllinalool/*trans*‐nerolidol synthases. (b) Co‐expression of SiTPS28 or SiTPS29 with the *ent*‐copalyl pyrophosphate (*ent*‐CPP) synthase (CPS), ZmAN2 (Harris *et al.*, 2005) or the (+)‐CPP synthase, IrTPS3, from *Isodon rubescens* (Pelot *et al.*, 2017). (c) The GC‐MS traces of products resulting from *Escherichia coli* co‐expression assays of SiTPS28 and SiTPS8 with *E*,*E*‐farnesyl pyrophosphate (FPP) produced by the maize FPP synthase ZmFPPS (Cervantes‐Cervantes *et al.*, 2006). (d) The GC‐MS traces of products resulting from *E. coli* co‐expression assays of SiTPS8 with the *ent*‐CPP synthase ZmAn2. (e) The GC‐MS traces of products resulting from *E. coli* co‐expression assays of SiTPS8 with the grand fir (*Abies grandis*) abietadiene synthase variant D621A that produces (+)‐CPP (Cyr *et al.*, 2007; Morrone *et al.*, 2010). (f) The GC‐MS traces of products resulting from *E. coli* co‐expression of SiTPS8 with the *syn*‐CPP synthase OsCPS4 (Xu *et al.*, 2004). (g) Mass spectrum of the major product of the coupled reaction of ZmAn2 and SiTPS8 (compound 8) and NMR‐based identification of the product as pimara‐8,15‐diene. (h) Mass spectrum of the primary product of the coupled reaction of OsCPS4 and SiTPS8 (compound 14) and NMR‐verified structure of the product as *syn*‐pimara‐7,15‐diene. Mass spectra for all detected TPS products are given in Figure S3. 2, *ent*‐kaurene; 6, geranyllinalool; 7, *trans*‐nerolidol; 8, *ent*‐pimara‐8,15‐diene; 9, *ent*‐pimara‐8(14),15‐diene; 10, *ent*‐sandaracopimaradiene; 11, *ent*‐abietadiene; 12, sandaracopimaradiene; 13, abietadiene; 14, *syn*‐pimara‐7,15‐diene; 15, unknown diterpene; 16, *syn*‐pimara‐9,11‐diene.

Having identified the *ent*‐, (+)‐ or *syn*‐CPP activity of the four class II diTPSs present in the *S. italica* genome, we set out to functionally analyze the identified *S. italica* class I TPSs using co‐expression in *N. benthamiana* or *E. coli*. Individual expression of the class I TPS candidates SiTPS1 and SiTPS2 (sharing 82% amino acid sequence identity) in *N. benthamiana* resulted in the formation of geranyllinalool and minor amounts of *trans*‐nerolidol, derived from GGPP and FPP, respectively, endogenously produced in *N. benthamiana* (Figures 3a and S3). Next, to verify the predicted *ent*‐kaurene synthase activity of the two class I diTPSs SiTPS28 and SiTPS29, co‐expression with the *ent*‐CPP synthase from maize (ZmAn2) (Harris *et al.*, 2005) or a (+)‐CPP synthase IrTPS3 from *Isodon rubescens* (Pelot *et al.*, 2017) (used here in place of SiTPS9 due to higher catalytic activity in *N. benthamiana*) were performed. Both SiTPS28 and SiTPS29 showed activity when combined with *ent*‐CPP synthases, whereas no class I diTPS products were detected with (+)‐CPP as a substrate, identifying these enzymes as *ent*‐kaurene synthases (Figures 3b and S5). Close phylogenetic relatedness of SiTPS28 to ZmTPS1 further indicated a possible dual diTPS and sesqui‐TPS activity, as previously demonstrated for ZmTPS1 (Fu *et al.*, 2016). Corresponding *E. coli* co‐expression assays of SiTPS28 with the maize *trans*‐FPP synthase, ZmFPPS (Cervantes‐Cervantes *et al.*, 2006), indeed resulted in the formation of *trans*‐nerolidol in addition to the *ent*‐kaurene synthase activity of SiTPS28 (Figure 3c).

To examine the class I diTPSs with probable functions in specialized diterpenoid metabolism (SiTPS5, SiTPS8, SiTPS13), *E. coli* co‐expression assays were performed with *ent*‐, (+)‐ and *syn*‐CPP as substrates using, as partnering class II diTPSs, the *ent*‐CPP synthase ZmAn2, the rice *syn*‐CPP synthase OsCPS4 and a grand fir (*Abies grandis*) abietadiene synthase variant producing (+)‐CPP (Morrone *et al.*, 2010). No class I diTPS products were detectable when co‐expressing SiTPS5 and SiTPS13 in *E. coli* with enzymes producing *ent*‐CPP, (+)‐CPP or *syn‐*CPP or in *N. benthamiana* expression assays with the (+)‐CPP synthase SiTPS9 or the *ent*‐CPP synthases SiTPS34/35 (Figure S6). In contrast, SiTPS8 showed catalytic promiscuity, converting all CPP stereoisomers (Figure 3d,f). The SiTPS8‐catalyzed conversion of *ent*‐CPP yielded multiple diterpene products, including *ent*‐pimara‐8(14),15‐diene, *ent*‐sandaracopimaradiene, *ent*‐abietadiene and a major product (product 8) that featured dominant mass ions of *m*/*z* 133, 161, 187, 257 and 272, indicating a related diterpene olefin (Figure 3d,g). To structurally identify product 8 via NMR analysis, in excess of 1 mg of the product was generated using large‐scale *E. coli* co‐expression assays and purified by silica chromatography and semi‐preparative HPLC (Murphy *et al.*, 2019). The purified product was then subject to 1D (^1^H and ^13^C) and 2D NMR (HSQC, COSY, HMBC, H2BC) analyses that identified the compound as *ent*‐pimara‐8,15‐diene, tentatively assigning the same *ent*‐stereochemistry at C9 (^13^CH_2_ 20.67 p.p.m.) and C10 (^13^CH_3_ 19.43 p.p.m.) based on *ent*‐CPP as the initial substrate. Similarly, stereochemistry of methyl groups at C18 (CH_3_, ^13^C 21.68 p.p.m.) and C19 (CH_3_, ^13^C 33.25 p.p.m.) was tentatively assigned based on *ent*‐CPP as substrate and mutual HMBC correlations to each other as well as C3 (CH_2_, ^13^C 41.86 p.p.m.), C4 (C, ^13^C 33.32 p.p.m.) and C5 (CH, ^13^C 51.82 p.p.m.), whereas the defining double bond between quaternary carbons C8 (^13^C 124.15 p.p.m.) and C9 (^13^C 136.61 p.p.m.) was verified by HMBC correlations with the neighboring C14 (CH_2_, ^13^C 42.48 p.p.m.), as well as the double bond between C15 (CH, ^13^C 149.45 p.p.m.) and C16 (CH_2_, ^13^C 109.43 p.p.m.) with HMBC correlation to C17 (CH_3_, ^13^C 23.20 p.p.m.) (Figures 3g and S7). SiTPS8‐catalyzed conversion of (*+*)‐CPP yielded abietadiene and sandaracopimaradiene as a minor byproduct (Figure 3e). Additionally, co‐expression of SiTPS8 with a *syn*‐CPP synthase yielded *syn*‐pimara‐9(11),15‐diene and two unidentified diterpene products (Figure 3f). The major product (compound 14) featured characteristic labdane diterpene mass fragments of *m*/*z* 229, 257 and 272. The NMR analysis of the purified compound identified it as *syn*‐pimara‐7,15‐diene as based on a comparison with published NMR spectra (Mafu *et al.*, 2016; Ye *et al.*, 2017) (Figures 3h and S8). Beyond its diTPS activity, co‐expression of SiTPS8 with ZmFPPS resulted in *trans*‐nerolidol, highlighting the substrate promiscuity of this enzyme (Figure 3c). Mass spectra of all identified class I TPS products are given in Figure S5.

### Functional characterization of selected class I sesquiterpene synthases from *S. italica*

**Figure 4:**
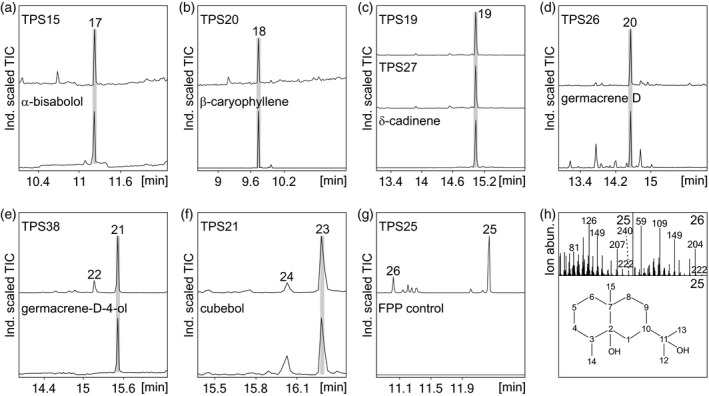
Functional characterization of the *Setaria italica* class I sesquiterpene synthases. (a)–(e) The GC‐MS traces of reaction products resulting from *Nicotiana benthamiana* co‐expression assays of the focal *S. italica* sesquiterpene synthases with product identification by comparison with authentic standards identified SiTPS15 as an α‐bisabolol synthase (a), SiTPS20 as a β‐caryophyllene synthase (b), SiTPS19 and SiTPS27 as δ‐cadinene synthases (c), SiTPS26 as a germacrene d‐synthase (d) and SiTPS38 as a germacrene‐d‐4‐ol synthase (e). (f), (g) The GC‐MS traces of reaction products resulting from *Escherichia coli* co‐expression assays of the maize *E*,*E*‐farnesyl pyrophosphate (*E*,*E*‐FPP) synthase, ZmFPPS (Cervantes‐Cervantes *et al.*, 2006), with focal *S. italica* sesquiterpene synthases identified SiTPS21 as a cubebol synthase (f), whereas SiTPS25 produced two distinct products, compound 25 and a minor byproduct, compound 26 (g). (h) Mass spectrum of compounds 25 and 26, and NMR‐verified structure of compound 25 identifying the SiTPS25 product as eudesme‐2,11‐diol. Mass spectra for all detected compounds are given in Figure S3. 17, α‐bisabolol; 18, β‐caryophyllene; 19, δ‐cadinene; 20, germacrene D; 21, germacrene‐D‐4‐ol; 22, unidentified terpene product; 23, cubebol; 24, unidentified terpene product; 25, eudesme‐2,11‐diol; 26, unidentified terpene product.

Activity screening of selected *S. italica* sesqui‐TPS candidates using *N. benthamiana* and *E. coli* co‐expression with ZmFPPS showed several different TPS activities (Figure 4). *Nicotiana benthamiana* expression revealed SiTPS15 as an α‐bisabolol synthase, SiTPS20 as a β‐caryophyllene synthase, SiTPS19 and SiTPS27 as δ‐cadinene synthases and SiTPS26 as a germacrene d‐synthase compared with commercial standards, while SiTPS38 produced germacrene‐d‐4‐ol as verified by comparison with the product of a δ‐cadinene synthase variant (GhCAD:N403P/L405H) from cotton (*Gossypium arboreum*) (Yoshikuni *et al.*, 2006) (Figures 4a,e and S5). Co*‐*expression of enzymes not showing activity in *N. benthamiana* with ZmFPPS in *E. coli* identified SiTPS21 as a cubebol synthase as validated by comparison with the product profile of cubebol synthase from *Coniophora puteana* (Mischko *et al.*, 2018) (Figures 4e,f and S5). The primary product of SiTPS25 featured mass ions of *m*/*z* 161, 207, 222 and 240 (Figure 4g,h), which closely matched the fragmentation pattern of the sesquiterpene alcohol eudesme‐2,11‐diol, recently identified in maize (Liang *et al.*, 2018). Additional 2D NMR analysis (H2BC and HMBC) of the purified compound verified the presence of two hydroxyl groups at quaternary carbons C2 (C, ^13^C 74.257 p.p.m.) and C11 (C, ^13^C 73.090 p.p.m.) and identified the product as eudesme‐2,11‐diol (Figures 4h and S9).

### Identification of SiCYP99A17 as a P450 catalyzing C19‐hydroxylation of labdane scaffolds

**Figure 5:**
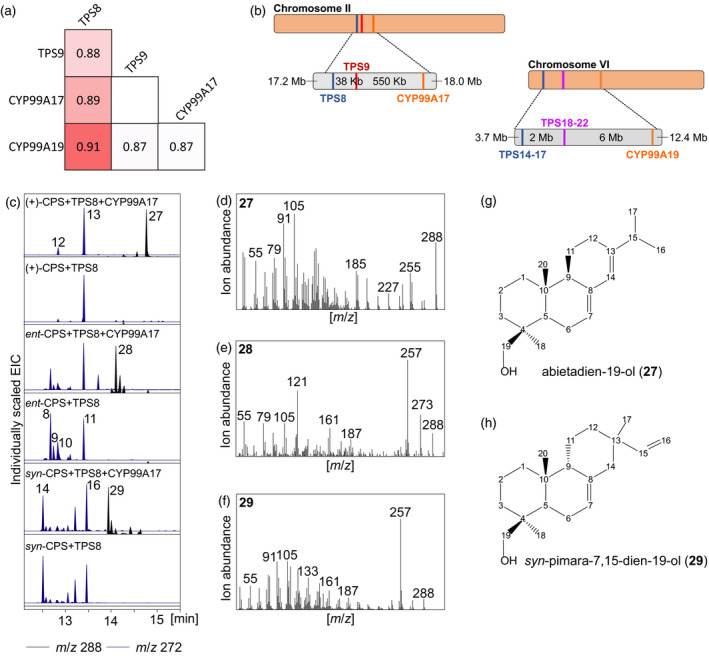
Identification of *Setaria italica* CYP99A17 as a diterpene monohydroxylase. (a) Pearson correlation coefficients of *SiTPS8*, *SiTPS9* and *CYP99A17* (data obtained from Phytozome, https://phytozome.jgi.doe.gov). (b) Chromosomal location of *SiTPS8*, *SiTPS9* and *CYPP99A17* on *S. italica* chromosome 2. (c) The GC‐MS extracted ion chromatograms (EIC) for dominant mass ions of *m*/*z* 272 (blue) and 288 (black) of reaction products resulting from *Escherichia coli* co‐expression assays of SiTPS8 with (+)‐, *ent*‐ or *syn*‐copalyl pyrophosphate (CPP) synthases and CYP99A17. (d)–(f) Mass spectra of SiTPS8‐CYP99A17 products with (+)‐CPP (d), *ent*‐CPP (e) or *syn*‐CPP (f) as a substrate. (g) Structure of CYP99A17 product 27 as abietadien‐19‐ol as verified by NMR analysis. (h) Structure of CYP99A17 product 29 as *syn*‐pimara‐7,15‐dien‐19‐ol as verified by NMR analysis.

Monocot diterpenoids are almost invariably functionally modified by P450 enzymes, with members of the CYP701, CYP71, CYP76 and CYP99 subfamilies having been shown to function in rice and maize diterpenoid metabolism (Swaminathan *et al*, 2009; Wang *et al.*, 2011; Wu *et al.*, 2011; Wang *et al.*, 2012a; Wang *et al.*, 2012b; Mao *et al.*, 2016; Mafu *et al.*, 2018; Ding *et al.*, 2019). Previous rice studies further revealed patterns of gene co‐regulation and genomic clustering of *diTPSs* and *P450s* involved in the biosynthesis of diterpenoid phytoalexins, such as momilactones and phytocassanes (Wilderman *et al.*, 2004; Prisic *et al.*, 2004; Shimura *et al.*, 2007; Swaminathan *et al.*, 2009; Boutanaev *et al.*, 2015). Based on these insights, we analyzed publicly available gene co‐expression data (https://phytozome.jgi.doe.gov) to identify similar *diTPS*/*P450* co‐expression patterns in *S. italica*. Indeed, significant co‐expression was observed for *SiTPS8*, *SiTPS9* and two P450 candidates, namely *Seita.2G145800* and *Seita.6G139100*, henceforth designated *CYP99A17* and *CYP99A19*, respectively (Figure 5a, Table S1). Both P450s showed best BLAST matches against rice CYP99A2 and CYP99A3 that catalyze the consecutive oxidation at C19 of *syn*‐pimara‐7,15‐diene in momilactone biosynthesis (Wang *et al.*, 2011; Kitaoka *et al.*, 2015b). Pearson‐correlated gene expression between *SiTPS9* and *SiTPS8* (0.88), *CYP99A17* and *SiTPS8* (0.89), *CYP99A19* and *SiTPS9* (0.87) and *CYP99A19* and *SiTPS8* (0.91) supported a functional relationship between these genes (Figure 5a). Further mining of the *S. italica* genome regions upstream and downstream of the identified TPS loci revealed that *CYP99A17* was located proximal to *SiTPS8* and *SiTPS9* on chromosome 2 with a distance of about 550 kb from *SiTPS9* and about 590 kb from *SiTPS8* (Figure 5b). To biochemically characterize CYP99A17 and CYP99A19, the corresponding genes were synthesized as codon‐optimized and N‐terminally truncated (removal of the predicted endoplasmic reticulum membrane anchor; Table S1). Since *SiTPS8* showed correlated gene expression with both *CYP99A17* and *CYP99A19*, combinatorial activity assays were performed for both P450s via *E. coli* co‐expression with SiTPS8 and an *ent*‐, *syn*‐ or (*+*)‐CPP synthase, thus encompassing all specialized labdane diterpene scaffolds identified in this study. No P450 products were observed for CYP99A19 with any of the tested diTPS combinations (Figure S10), whereas co‐expression of CYP99A17 showed product formation with all diTPS combinations to form several new products with mass fragments of *m*/*z* 288, indicating hydroxylated labdane diterpenoid scaffolds (Figure 5c). Combining HSQC, HMBC and NOESY analyses identified the purified products of the coupled activity of (+)‐CPP synthase, SiTPS8 and CYP99A17 as abietadien‐19‐ol as compared with previously reported NMR data (Lee *et al.*, 2001) (Figures 5d,g and S11), the presence of a hydroxyl group at C19 (CH_2_OH, ^13^C 64.99 p.p.m.) and NOESY correlation with C20 (CH_3_, ^13^C 14.69 p.p.m.). Low abundance of the product formed by SiTPS8 and CYP99A17 with *ent*‐CPP as the substrate prevented compound purification and NMR structural verification (Figure 5e). The product resulting from the co‐expression of SiTPS8 and CYP99A17 with a *syn*‐CPP synthase was tentatively identified via NMR analysis and comparison with reference spectra as *syn*‐pimara‐7,15‐dien‐19‐ol (Figure 5f,h and S12) (Wang *et al* *.*, 2011).

### Abundance of terpene‐metabolic genes in *S. italica*

**Figure 6:**
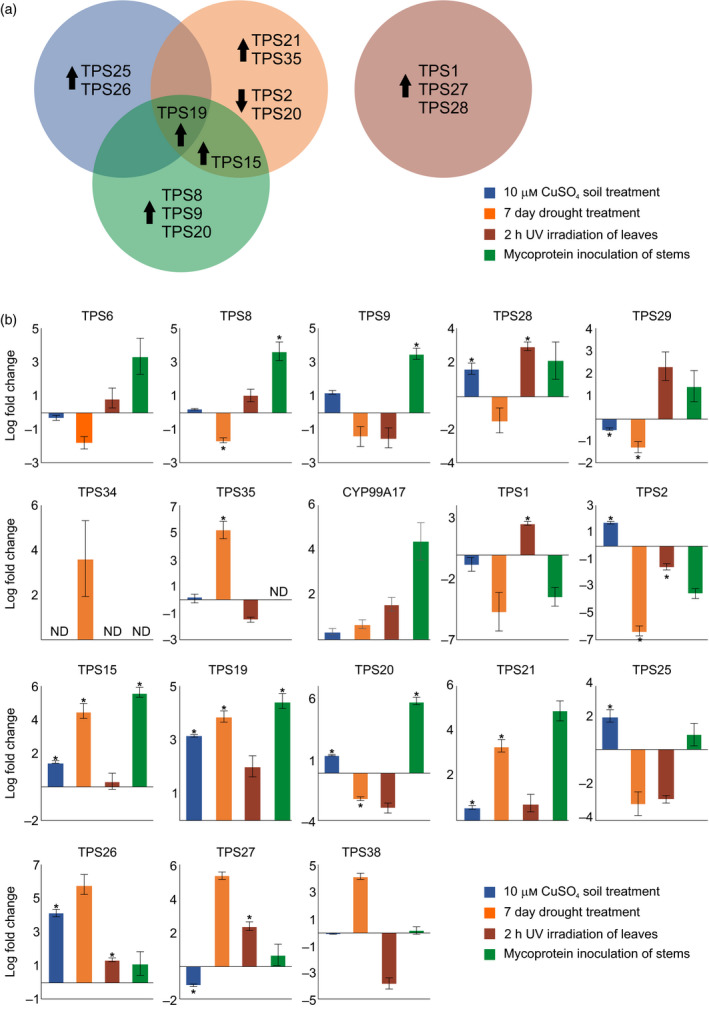
Abundance of terpene synthase genes and CYP99A17 in *Setaria italica*. (**a**) Venn diagram representation of *S. italica* genes significantly up‐ or downregulated by two‐fold or more in organs. Treatments included root oxidative stress (soil treatment with 10 mm CuSO_4_ for 48 h; blue), drought stress (seven consecutive days with relative water content = 30%; orange), leaf exposure to UV irradiation (254 nm) for 2 h (purple) and pathogen stress (approximated by stem inoculation with Quorn^TM^ mycoprotein from *Fusarium venenatum*; green). Transcript levels were measured by qPCR gene expression analysis with normalization to the internal reference gene *SiEF‐1α* (Kumar *et al.*, 2013) using log fold‐change (ΔΔCt) compared with untreated control samples (*n* = 3). Statistically significant differences between treated and control samples are based on the Welch two‐sample *t*‐test (*P* < 0.05; Figure S10). (b) Heatmap and dendrogram showing gene expression of *S. italica* terpene synthases (TPSs) based on publicly available transcript data (https://phytozome.jgi.doe.gov/phytomine) with red indicating high expression and blue representing low expression levels.

Numerous gene expression studies across poaceous crops have demonstrated stress‐inducible transcriptome accumulation of *TPSs* and *P450s* in response to biotic and/or abiotic stressors (reviewed in Schmelz *et al.*, 2014), suggesting a related defensive response in *S. italica*. To test this hypothesis, we performed quantitative (q)PCR‐based gene expression studies of the identified *TPS* and *P450* genes following different abiotic and biotic stress treatments. Abiotic stresses were applied to roots of 4‐week‐old *S. italica* plants in the form of oxidative stress by soil treatment with 10 mm CuSO_4_ for 2 days, in form of drought stress by lack of watering for seven consecutive days and by UV irradiation of leaves for 2 h. In addition, pathogen stress was approximated by treatment of stems of 10‐week‐old *S. italica* plants with mycoprotein derived from *Fusarium venenatum* (Quorn^TM^) (Figures 6 and S13). In addition, publicly available data on *S. italica* tissue‐ and treatment‐specific gene expression (https://phytozome.jgi.doe.gov) were queried. Although comparison of these experimental and public data has to be viewed with caution given the use of, for example, different growth conditions and plant ages at harvest, some notable trends could be observed. *SiTPS19* was the only transcript showing significantly induced expression in response to all stresses, except for UV irradiation (Figure 6), despite its apparently higher abundance in leaf and panicle (Figure S13). *SiTPS15* was also most abundant in panicles and showed inducible expression patterns upon drought stress and mycoprotein treatment (Figures 6 and S13). All other TPS genes showed significant changes in gene expression predominantly under only one of the applied treatments. *SiTPS26* showed the highest transcript changes in response to oxidative stress, although it was most abundant in leaves (Figures 6 and S13). Increases in gene expression in response to drought stress were highest for *SiTPS19* and *SiTPS35* (Figure 6). Ultraviolet irradiation caused increased transcript abundance most significantly for *SiTPS1*, *SiTPS27* and *SiTPS28*, consistent with the primary occurrence of these genes in leaf and panicle tissue (Figures 6 and S13). *SiTPS8*, *SiTPS9, SiTPS15*, *SiTPS19* and *SiTPS20* were also most abundant in aerial *S. italica* organs and showed the most significantly induced gene expression in response to mycoprotein treatment, indicating a possible function in defense against above‐ground pathogens (Figures 6 and S13).

### Occurrence of TPS and P450 products *in planta*

To examine the presence of enzyme products of stress‐inducible TPSs and CYP99A17 *in planta*, control and stress‐treated *S. italica* leaf, stem and root samples used for gene expression studies were subjected to organic solvent extraction and targeted metabolite analysis using ultra performance liquid chromatography (UPLC)–hydrophilic interaction liquid chromatography (HILIC)–tandem mass spectrometry (MS/MS) against the purified enzyme products as authentic standards. Of the 20 identified TPS products, germacrene‐d‐4‐ol and the diterpene alcohols abietadien‐19‐ol and *syn*‐pimara‐7,15‐dien‐19‐ol were detected in the tested plant tissues under both controlled and stress‐treated conditions (Figure S14). Germacrene‐d‐4‐ol was detected exclusively in roots with product identification based on a dominant mass fragment *m*/*z* 205.195076 corresponding to the dehydrated protonated adduct ([M‐H_2_O + H]^+^, parent mass ion [M]^+^ = 222.1984) (Figure S14). By contrast, abietadien‐19‐ol and/or *syn*‐pimara‐7,15‐dien‐19‐ol (parent ion [M]^+^ *m*/*z* 288.2453) were present in leaves, stems and roots as verified by characteristic mass ions *m*/*z* 289.2525 and 271.2419 corresponding to the protonated adducts ([M + H]^+^) and the protonated dehydrated adducts ([M‐H_2_O + H]^+^), respectively (Figure S14). However, overlapping retention times and a nearly identical fragmentation pattern of abietadien‐19‐ol and *syn*‐pimara‐7,15‐dien‐19‐ol prevented an unambiguous verification if both or only one compound occurs *in planta*. Furthermore, abietadien‐19‐ol and/or *syn*‐pimara‐7,15‐dien‐19‐ol were detected in both stress‐exposed and control plants with no apparent difference in abundance.

### Antifungal activity of terpenes from select *S. italica* TPSs

**Figure 7:**
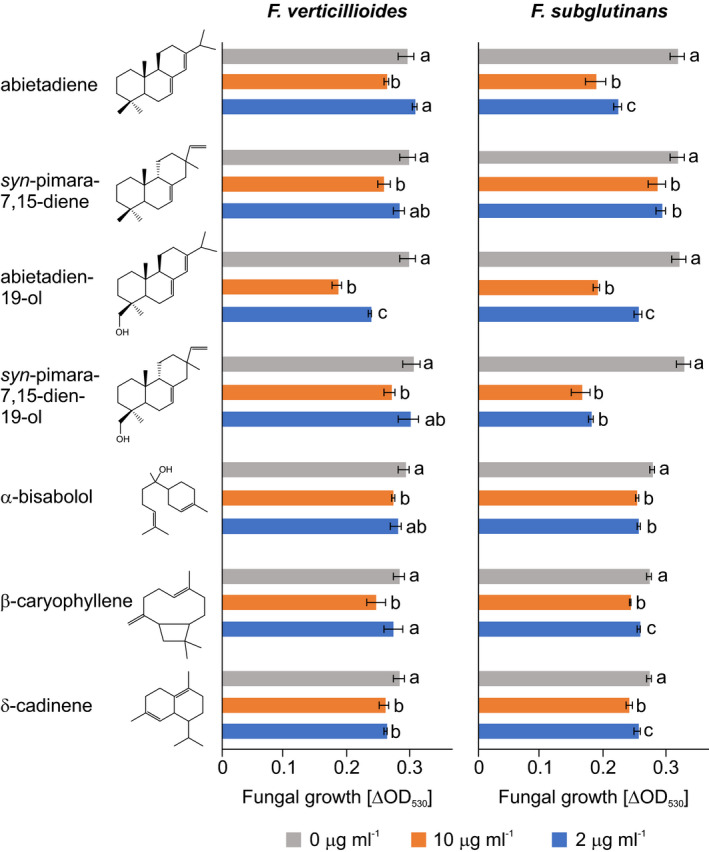
*In vitro* antifungal activity of selected terpenoid products***.*** Average growth (OD_530_) of *Fusarium verticillioides* and *Fusarium subglutinans* measured over a 48 h time course in defined minimal broth medium using a microtiter plate assay in the absence and presence of purified terpenoids at a concentration of 0 µg ml^−1^ (grey), 2 µg ml^−1^ (blue) and 10 µg ml^−1^ (orange). Error bars represent propagated SE values (*n* = 4) and letters represent significant differences at *P* < 0.05 as measured using analysis of variance and Tukey's honestly significant difference tests to correct for multiple comparisons between control and treatments.

Fungal‐elicited transcript accumulation of *SiTPS6*, *SiTPS8*, *SiTPS9*, *SiTPS15*, *SiTPS19*, *SiTPS20* and *SiCYP99A17* suggested possible antifungal bioactivities of the corresponding enzyme products (Figure 6). To investigate possible antifungal activity of the corresponding terpenoids, *in vitro* fungal growth inhibition assays were performed using commercial standards of α‐bisabolol, β‐caryophyllene and δ‐cadinene, and purified enzyme products of abietadiene, *syn*‐pimara‐7,15‐diene, abietadien‐19‐ol and *syn*‐pimara‐7,15‐dien‐19‐ol. The purified SiTPS21 product cubebol proved to be unstable at the assay temperature and was excluded from further analysis. The fungal pathogens *Fusarium verticillioides* and *Fusarium subglutinans* were chosen for bioactivity assays, as these species have been shown to cause pathogenic symptoms in *S. italica* (Nwanma *et al.*, 1991; Besharati fard *et al.*, 2014; Li *et al.*, 2015). Overall, at concentrations of 2 µg ml^−1^ and 10 µg ml^−1^ all tested metabolites showed no or moderate inhibitory activity on fungal growth within the 48 h assay duration (Figure 7). The strongest antifungal activity was observed for abietadien‐19‐ol, with a 40% reduction of *F. verticillioides* and *F. subglutinans* growth after 48 h at a metabolite concentration of 10 µg ml^−1^. Interestingly, the corresponding olefin precursor abietadiene exhibited a similar antifungal activity against *F. subglutinans* but was less effective in inhibiting the growth of *F. verticillioides* with only a 13% reduction in fungal growth at the same metabolite concentration (Figure 7). *syn*‐Pimara‐7,15‐dien‐19‐ol showed a comparable antifungal efficacy, inhibiting growth of *F. subglutinans* by about 40% at both tested metabolite concentrations, but showing less impact on *F. verticillioides* with only a 6% decrease in fungal growth after 48 h even at the higher metabolite concentration. Unlike abietadiene, the corresponding olefin precursor of *syn*‐pimara‐7,15‐dien‐19‐ol, *syn*‐pimara‐7,15‐diene, showed only minor antifungal activity. Regardless of the metabolite concentration tested, the targeted sesquiterpenoids, α‐bisabolol, β‐caryophyllene and δ‐cadinene, reduced fungal growth at lower levels of 10–13% compared with the diterpene alcohols.

## DISCUSSION

Species‐specific terpenoid networks serve as important chemical defense mechanisms in monocot crops against both biotic and abiotic stressors. In particular, the potent pest‐ and disease‐protective functions of terpenoids in maize and rice have been well established (reviewed in Schmelz *et al.*, 2014; Murphy and Zerbe, 2020). In addition, diverse terpenoid metabolic networks comprising both common and unique pathway branches and products have been described in wheat and switchgrass, suggesting similar physiological roles (Wu *et al.*, 2012; Zhou *et al.*, 2012; Pelot *et al.*, 2018; Muchlinski *et al.*, 2019). Continued investigation of the biosynthesis, diversity and function of terpenoid chemical defense mechanisms can enable new strategies for improving crop resilience in the face of projected increased harvest losses caused by shifting environmental pressures and associated biotic and abiotic stressors that can overwhelm the natural defense systems of plants (Chakraborty and Newton, 2011; de Sassi and Tylianakis, 2012; Leng and Hall, 2019). In this context, we report here the discovery and functional analysis of the TPS family of *S. italica* as a model food and bioenergy species and an important grain resource across Asia and Africa (Huang *et al.*, 2016; Pant *et al.*, 2016).

**Figure 8:**
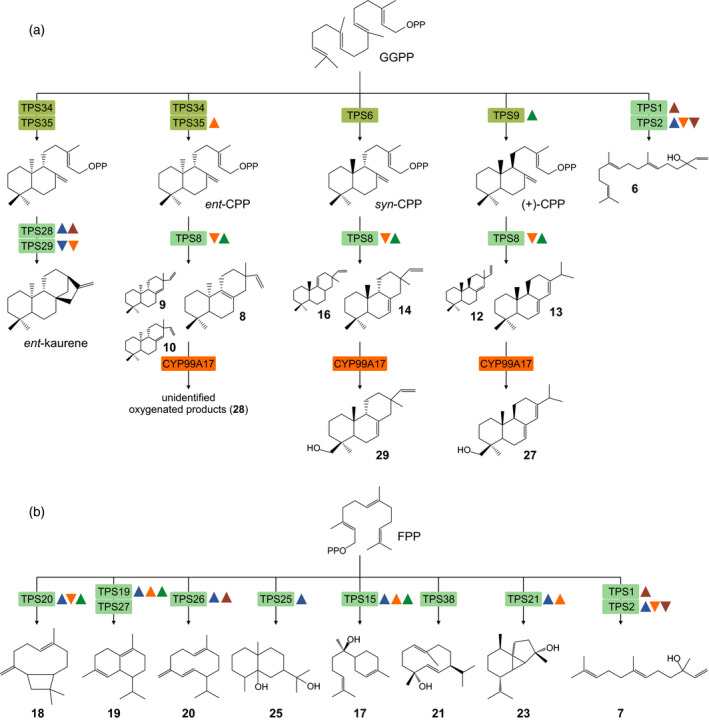
The *Setaria italica* terpenoid metabolic network. (a) Scheme of identified diterpenoid biosynthetic pathways in *S. italica*, involving monofunctional class II and class I diterpene synthases (diTPSs), as well as CYP99A17 that convert the central precursor C20 *E*,*E*,*E*‐geranylgeranyl pyrophosphate (GGPP) into a range of diterpenoid metabolites. (b) Scheme of sesquiterpenoid biosynthesis in *S. italica*, involving distinct class I TPSs converting the C15 precursor *E*,*E*‐farnesyl pyrophosphate (FPP) into distinct sesquiterpenoid metabolites. Triangles depict genes showing significant stress‐elicited expression in response to root oxidative stress with CuSO_4_ (blue), root drought stress (orange), leaf UV irradiation (brown), and stem inoculation with *Fusarium venenatum*‐derived mycoprotein (Quorn, green). 6, geranyllinalool; 7, *trans*‐nerolidol; 8, *ent*‐pimara‐8,15‐diene; 9, *ent*‐pimara‐8(14),15‐diene; 10, *ent*‐sandaracopimaradiene; 12, abietadiene; 13, sandaracopimaradiene; 14, *syn*‐pimara‐7,15‐diene; 16, *syn*‐pimara‐9(11),15‐diene; 17, α‐bisabolol; 18, β‐caryophyllene; 19, δ‐cadinene; 20, germacrene D; 21, germacrene‐D‐4‐ol; 23, cubebol; 25, eudesme‐2,11‐diol; 27, abietadien‐19‐ol; 29, *syn*‐pimara‐7,15‐dien‐19‐ol.

The *S. italica* TPS family represents a medium‐sized gene family of 32 members similar to those of related diploid grasses, including maize, rice and wheat (Schmelz *et al.*, 2014; Murphy and Zerbe, 2020). Diterpenoid biosynthesis in *S. italica* is controlled by four class II diTPSs, two of which, SiTPS34 and SiTPS35, represent catalytically redundant *ent*‐CPP synthases (Figure 8). Similar *ent*‐CPP synthase pairs are also present in maize (Bensen *et al.*, 1995; Harris *et al.*, 2005), rice (Prisic *et al.*, 2004) and switchgrass (Pelot *et al.*, 2018), and genetic studies showed these enzyme pairs to be functionally separate, with roles in GA biosynthesis and specialized metabolism, respectively (Bensen *et al.*, 1995; Prisic *et al.*, 2004; Sakamoto *et al.*, 2004; Vaughan *et al.*, 2015). Inducible gene expression of *SiTPS35*, but not *SiTPS34*, under drought stress and phylogenetic clustering of SiTPS34 with the generalized GA‐biosynthetic maize proteins ZmAn1 and SiTPS35 with the specialized *Zm*An2 indicate a similar functional separation in *S. italica*. Syntenic conservation of *SiTPS34* and the *ent*‐*kaurene synthase* *SiTPS29* with known or predicted *ent*‐*CPP synthase*/*ent*‐*kaurene* *synthase* pairs in all seven analyzed Poaceae genomes support this hypothesis. In contrast, only weak gene synteny was observed for the additional *ent*‐CPP *synthase*/*ent*‐*kaurene synthase* pair in *S. italica*, *SiTPS35* and *SiTPS28*, where *SiTPS35* lacks syntenic orthologs in multiple species and *SiTPS28* lacks a syntenic ortholog in rice. Although elucidation of *SiTPS34* and *SiTPS35* will require genetic analyses beyond the scope of this study, these combined results suggest that *SiTPS34* and *SiTPS29* function in GA biosynthesis, whereas *SiTPS35* and *SiTPS28* are more likely to be involved in specialized diterpenoid metabolism. Dual activity of SiTPS28 in forming *ent*‐kaurene and converting FPP into *trans*‐nerolidol also supports this conclusion.

The additional presence of a *syn*‐CPP synthase (SiTPS6) and a (+)‐CPP synthase (SiTPS9) sets the catalytic range of class II diTPSs in *S. italica* apart from all other poaceous crop species investigated so far, which, according to current knowledge, feature enzymes for producing only *syn*‐CPP (e.g. rice, switchgrass) or (+)‐CPP (e.g. maize, wheat) in addition to the ubiquitously occurring *ent*‐CPP (Schmelz *et al.*, 2014; Murphy *et al.*, 2018; Pelot *et al.*, 2018). Although syntenic orthologs for *SiTPS6* and *SiTPS9* were present in all analyzed genomes (except for *B. distachyon*), the respective maize, rice and switchgrass orthologs did not show functional conservation. For example, the *SiTPS6* orthologs of rice (OsCPS2) and maize (ZmCPS4) encode for an *ent*‐CPP synthase and 8,13‐CPP synthase, respectively, rather than *syn*‐CPP synthases (Prisic *et al.*, 2004; Murphy *et al.*, 2018). Likewise, of the identified maize and rice orthologs to the *(+)‐CPP synthase* *SiTPS9*, only *ZmCPS3* encodes for a (+)‐CPP synthase, whereas rice *OsCPS4* encodes a *syn*‐CPP synthase and maize ZmCPS4 forms 8,13‐CPP (Xu *et al.*, 2004; Otomo *et al.*, 2004; Murphy *et al.*, 2018). Close phylogenetic interrelations of SiTPS6 with ZmCPS4 and of SiTPS9 with ZmCPS3 and OsCPS4 are consistent with this functional divergence. Indeed, structure‐guided mutagenesis studies in several poaceous species highlight the ease with which neo‐functionalization of class II diTPSs may occur. For example, single active site residue substitutions are sufficient to alter the product specificity of the switchgrass 8,13‐CPP synthase PvCPS3 to the form (+)‐CPP, and similar active site mutations lead to a re‐programming of the rice OsCPS4 from *syn*‐CPP to 5,13‐halimadienyl pyrophosphate (HDP) as a major product (Potter *et al.*, 2016; Pelot *et al.*, 2018).

Next to the pair of *ent*‐kaurene synthases (SiTPS28 and SiTPS29), SiTPS8 showed expansive substrate promiscuity, converting all *S. italica* CPP stereoisomers into distinct pimaradiene, sandaracopimaradiene and abietadiene scaffolds (Figure 8). Several *ent*‐CPP‐ and *syn*‐CPP‐derived SiTPS8 products, including *syn*‐pimara‐7,15‐diene, *ent*‐pimara‐8,15‐diene, *ent*‐sandaracopimaradiene and (+)‐abietadiene, also occur in rice, switchgrass and presumably wheat (Xu *et al.*, 2007b; Zhou *et al.*, 2012; Pelot *et al.*, 2018). Additionally, SiTPS8 formed the previously unrecognized pimarane double bond isomer, *ent*‐pimara‐8,15‐diene, with the most closely related function described for the wheat class I diTPS, TaKSL4, that produces the corresponding (+)‐stereoisomer (Zhou *et al.*, 2012). Notably, in rice, *ent*‐sandaracopimaradiene and *syn*‐pimara‐7,15‐diene serve as precursors to oryzalexins and momilactones, respectively, with have demonstrated functions in pathogen defense (Atawong *et al.*, 2002; Hasegawa *et al.*, 2010; Toyomasu *et al.*, 2014). Abundance in above‐ground tissues and elicitation of *SiTPS8* gene expression in response to mycoprotein treatment may point toward similar functions in *S. italica*. However, unlike the upregulation of momilactone production in rice plants exposed to UV irradiation, *SiTPS8* expression was not elicited by UV light, indicating species‐specific differences. Consistent with this hypothesis, SiTPS8 seems to have evolved independently, since it is only distantly related to specialized βα‐domain enzymes of the TPS‐e/f clade (Morrone *et al.*, 2011; Zerbe and Bohlmann, 2015; Pelot *et al.*, 2018), and showed no gene synteny across the analyzed Poaceae genomes.

Downstream of diterpene scaffold formation, functional modifications typically initiated by P450 monooxygenases are key to the various bioactivities of the compound group (Banerjee and Hamberger, 2018). In rice, formation of momilactones from *syn*‐pimara‐7,15‐diene is catalyzed by members of the CYP99A family (Shimura *et al.*, 2007; Wang *et al.*, 2011). With CYP99A17, we also identified a functional CYP99 enzyme in *S. italica*, which is capable of converting several SiTPS8 products of *ent*‐, *syn*‐ and (+)‐stereochemistry (Figure 8). In addition to its more expansive substrate range, CYP99A17 differs from rice CYP99 enzymes in its catalytic sequence. Unlike rice CYP99A2/3, which oxygenate their respective substrates at C19 to generate the corresponding diterpene acids (Wang *et al.*, 2011), NMR analysis of selected CYP99A17 products showed that the enzyme catalyzed C19 hydroxylation reactions, as exemplified by the diterpene alcohol products *syn*‐pimara‐7,15‐dien‐19‐ol and abietadien‐19‐ol verified here. Mycoprotein‐elicited gene expression of *CYP99A17* in *S. italica* stems and, albeit moderate as compared with, for example, maize diterpenoids (Schmelz *et al.*, 2011; Mafu *et al.*, 2018), *in vitro* antifungal activity of abietadien‐19‐ol and *syn*‐pimara‐7,15‐dien‐19‐ol against *F. verticillioides* and *F. subglutinans* support possible roles of these pathway branches in disease resistance, although structurally distinct from those observed in rice. Considering the expansive functional decoration of many antimicrobial diterpenoids, it appears likely that the observed diterpene alcohols do not represent pathway end products but are further modified. The low abundance of abietadien‐19‐ol and/or *syn*‐pimara‐7,15‐dien‐19‐ol in *S. italica* tissues is consistent with this hypothesis.

Next to the diversity of diterpenoid products, several TPS enzymes were characterized that produce a range of sesquiterpenoids (Figure 8). Among these, the catalytically redundant SiTPS1 and SiTPS2 of the TPS‐g clade showed dual activity, converting FPP into *trans*‐nerolidol and GGPP into geranyllinalool. Related TPS‐g enzymes identified, for example, in Arabidopsis, switchgrass, tomato (*Solanum lycopersicum*) and maize function in forming the geranyllinalool‐derived 4,8,12‐trimethyltrideca‐1,3,7,11‐tetraene (TMTT) with antifeedant activity (Ament *et al.*, 2004; Herde *et al.*, 2008; Richter *et al.*, 2016; Muchlinski *et al.*, 2019). *SiTPS1* and *SiTPS2* are co‐located on chromosome 1 and show 82% protein sequence identity. Yet, unlike *SiTPS1*, *SiTPS2* lacks syntenic orthologs in several analyzed genomes and appears to be devoid of a plastidial transit peptide, supporting the emergence of *SiTPS2* through a more recent gene duplication event and possible alteration of compartmentalization and associated substrate restriction as also proposed for functionally redundant acyclic TPSs in switchgrass and snapdragon (*Antirrhinum majus*) (Nagegowda *et al.*, 2008; Muchlinski *et al.*, 2019). Similarly, two δ‐cadinene synthases, SiTPS19 and SiTPS27, showed catalytic redundancy, yet distinct patterns of pathway regulation. Here, *SiTPS19* expression was elicited in response to several stresses (except for UV irradiation) in roots and stems, whereas *SiTPS27* expression was most significantly increased in UV‐stressed leaves. The remaining characterized sesquiterpene synthases appear to occur as single‐copy genes in *S. italica*. Among these, identification of SiTPS20 as a β‐caryophyllene synthase is consistent with β‐caryophyllene production in rice and maize, where it is associated with volatile above‐ground herbivore defenses (Cheng *et al.*, 2007; Köllner *et al.*, 2008a). Beyond these commonly occurring sesquiterpenoids in monocot crops, several *S. italica* TPSs formed more unusual sesquiterpene alcohols, including germacrene‐d‐4‐ol, α‐bisabolol, cubebol and eudesme‐2,11‐diol, with as yet unknown biological functions. Production of cubebol and α‐bisabolol is broadly distributed across angiosperm and gymnosperm species, but this is less well described in Poaceous crops (Asadollahi *et al.*, 2010; Kamatou and Viljoen, 2010; Son *et al.*, 2014; Muchlinski *et al.*, 2019). Increased transcript abundance of the α‐bisabolol synthase, *SiTPS15*, and the cubebol synthase *SiTPS21* in response to drought stress and mycoprotein treatment may indicate functions in both biotic and abiotic stress responses. Similarly, detection of germacrene‐d‐4‐ol in extracts of *S. italica* roots (although at low abundance) and inducible expression of the corresponding biosynthetic gene, *SiTPS38*, in roots exposed to drought suggest a possible function in below‐ground stress responses. Biosynthesis of the dihydroxylated sesquiterpenoid eudesme‐2,11‐diol has only recently been described in monocots as a function of the maize ZmTPS16 (Liang *et al.*, 2018). The *S. italica* eudesme‐2,11‐diol synthase, SiTPS25, represents a syntenic ortholog of ZmTPS16, thus indicating a common ancestry. However, contrasting the pathogen‐inducible accumulation of eudesme‐2,11‐diol in maize roots, eudesme‐2,11‐diol was not detected in *S. italica* tissue extracts and no substantial changes in gene expression were observed under the tested stressors. Interestingly, although germacrene‐d‐4‐ol, α‐bisabolol, cubebol and eudesme‐2,11‐diol are less commonly observed in Poaceous species, the genomic regions encoding the corresponding TPS enzymes are syntenically conserved across the Poaceae species tested here (with the exception of *SiTPS15* that lacks an ortholog in *S. bicolor*). It can be speculated that species‐specific variation in the presence of these sesquiterpenoids is due to functional divergence of several such orthologs. Indeed, the switchgrass syntenic orthologs for *SiTPS15* and *SiTPS38* encode TPSs that produce structurally related terpenes to their *S. italica* counterparts, β‐bisabolene and germacrene d, respectively (Muchlinski *et al.*, 2019).

## EXPERIMENTAL PROCEDURES

### Plant material

Seeds of *S. italica* (Yugu1) were provided by Dr Katrien Devos (Department of Plant Biology, University of Georgia, USA). Seeds were germinated in Conviron TCR120 growth chambers (https://www.conviron.com/) under a photoperiod of 16 h, 60% relative humidity, 100 μmol m^−2^sec^−1^ light intensity and a day/night temperature cycle of 21ºC /18ºC. After 2 weeks, *S. italica* plants were either maintained in growth chambers for an additional 2 weeks (for drought, UV or CuSO_4_ treatments) or were grown in a greenhouse using nutrient water solution at an output of about 300 ml day^−1^ for 10 weeks under an ambient photoperiod and 22°C/17°C day/night temperatures (for mycoprotein assays). *Nicotiana benthamiana* plants were cultivated in growth chambers for a total of 5 weeks before use for *Agrobacterium*‐mediated co‐expression studies using *A. tumefaciens* GV3101.

### Gene discovery, synthesis and cloning

The TPS and P450 genes reported in this study were identified by querying the publicly available genomes of *S. italica* (v.2.2) and *S. viridis* (v.1.1) (https://phytozome.jgi.doe.gov/pz/portal.html) using Blast search against a manually curated plant TPS database (*E*‐value threshold of ≤ 1 × 10^–30^) (Zerbe *et al.*, 2013). For phylogenetic analysis, protein sequence alignments were generated using ClustalW2, curated with Gblocks, and maximum‐likelihood phylogenetic analyses were performed using PhyML‐aBayes v.3.0.1 beta with four rate substitution categories, the LG substitution model, a BIONJ starting tree and 1000 bootstrap repetitions (http://phylogeny.lirmm.fr). The phylogenetic tree was visualized using Interactive Tree of Life (https://itol.embl.de/).

Targeted TPS genes were synthesized as follows. For functional characterization of the encoded enzymes in *N. benthamiana*, full‐length native sequences were synthesized and inserted into the *Pac*I site of the pLIFE (pCAMBIA130035Su) expression vector. For co‐expression in *E. coli* N‐terminally truncated (lacking the predicted plastidial transit peptide) and codon‐optimized genes were generated and cloned into the pET‐DUET1 or pET28b(+) vectors (Novagen, https://www.emdmillipore.com/; Sigma, https://www.sigmaaldrich.com/). Gene synthesis was conducted by the US Department of Energy Joint Genome Institute, a DOE Office of Science User Facility, under contract no. De‐AC02‐05CH11231. The P450 genes *CYP99A17* and *CYP99A19*, were synthesized as codon‐optimized and N‐terminally modified (removal of the endoplasmic reticulum membrane anchor) genes and cloned into the pET‐DUET1 vector also containing the *Z. mays* cytochrome P450 reductase ZmCPR2 for expression in *E. coli*. (Table S1) (Wang *et al.*, 2011; Mafu *et al.*, 2018). Additional genes used for producing authentic standards, namely *G. arboreum* germacrene‐d‐4‐ol/δ‐cadinene synthase (NM_001330020) variant N403P/L405H and *C. puteana* cubebol synthase, Copu3 (XP_007765978), were synthesized for expression in *E. coli* and cloned into pET‐DUET1 according to published literature (Yoshikuni *et al.*, 2006; Mischko *et al.*, 2018).

### Synteny analysis

The latest coding sequence and gene annotations (GFF) for each species were obtained from the Phytozome database (https://phytozome.jgi.doe.gov/pz/portal.html). The GFF files were converted to BED files using the Python package JCVI (https://pypi.org/project/jcvi/). Candidate TPS genes were aligned to the candidate species using the MCscan workflow contained within JCVI. Genes were aligned using a ‘cscore’, the ratio of BLAST hit to the best BLAST hits to either the query or hit with a cutoff at 0.8. The best alignment for each *TPS* gene was considered a potential ortholog. If a tie occurred, the alignments of the 25 genes both upstream and downstream of the candidate gene were inspected to determine blocks of synteny, which were used to break the tie. All candidate *TPS* gene alignments were plotted using the graphics library of JCVI utility libraries | Zenodo (https://zenodo.org/record/31631#.XVX8_uhKjct.; accessed 3 August, 2019).

### Enzyme characterization in *E. coli*

For microbial expression assays, individual TPS constructs were transformed into *E. coli* BL21DE3‐C41 along with plasmids encoding for key genes of the MEP pathway, and the *A. grandis* GDPP synthase for diterpene production or a *Z. mays* FPP synthase (ZmFPPS) for sesquiterpene production, as described previously (Cyr *et al.*, 2007; Morrone *et al.*, 2010). Additionally, for coupled SiTPS8 and CYP99A17 (pET‐DUET1:CYP99A17/ZmCPR2C) co‐expression assays in *E. coli* we used, the *ent*‐CPP synthase from *Z. mays* (ZmAn2), the rice *syn*‐CPP synthase (OsCPS4) or the *A. grandis* abietadiene synthase variant D621A that produces CPP of (+)‐stereochemistry (Cyr *et al.*, 2007; Morrone *et al.*, 2010; Mafu *et al.*, 2018). Cultures were grown at 37°C and 180 r.p.m. in 50 ml of Terrific Broth (TB) medium to an OD_600_ of about 0.6 before cooling to 16°C and induction with 1 mm isopropyl‐β‐d‐1‐thiogalactopyranoside for 72 h with the addition of 25 mm sodium pyruvate. For co‐expression assays of P450s, 75 mg L^−1^ aminolevulinic acid and 5 mg L^−1^ riboflavin were added during the 72 h incubation. Products were extracted with 50 ml of hexane, air dried, and resuspended in 1 ml of *n*‐hexane for GC‐MS analysis.

### Transient co‐expression in *N. benthamiana*

The TPS candidates were expressed in *N. benthamiana* using the pLIFE (pCAMBIA130035Su) vector system in conjunction with the RNA‐silencing suppressor gene *p19* (Zerbe *et al.*, 2013). The TPS genes cloned into individual pLIFE expression vectors were transformed separately into *A. tumefaciens* strain GV3101. Bacterial cultures were grown at 28°C in Luria‐Bertani (LB) medium supplemented with 50 mg L^−1^ kanamycin, pelleted and resuspended to a final OD_600_ of 1.0 in 10 mm MES buffer with 10 mm MgCl_2_. Following incubation for 2 hours at 22°C and gentle shaking, cell suspensions were mixed and syringe‐infiltrated into the underside of the leaves of 5‐week‐old *N. benthamiana* plants. Transfected plants were maintained for 4 days before metabolites were extracted with 1.5 ml of hexane from a single transformed leaf and analyzed via GC‐MS.

### The GC‐MS analysis of enzyme products

Metabolite extracts from plant material or microbial cultures were freed from residual water by the addition of anhydrous Na_2_SO_4_, dried under a N_2_ stream and re‐dissolved in 1 ml of hexane. Extracts from microbial co‐expression assays with CYP99A17 and CYP99A19 were further derivatized using tetramethylsilane (TMS)‐diazomethane (Millipore, https://www.emdmillipore.com/; Sigma). A GC‐MS analysis was performed on an Agilent 7890B GC (https://www.agilent.com/) interfaced with a 5977 Extractor XL MS detector at 70 eV and 1.2 ml min^−1^ He flow, using a HP5‐ms column (30 m, 250 µm i.d., 0.25 µm film) and the following GC parameters for detection of diterpene products and sesquiterpene products of SiTPS15 and SiTPS20: 50°C for 1 min, 20°C min^−1^ to 300°C, hold 3 min with pulsed splitless injection at 250°C and 50°C oven temperature. The GC‐MS parameters for detection of sesquiterpene products of SiTPS19, SiTPS21, SiTPS26, SiTPS27 and SiTPS38 were: 50°C for 1 min, 10°C min^−1^ to 300°C, hold for 3 min with pulsed splitless injection at 250°C and 50°C oven temperature. The MS data from a 90–600 mass‐to‐charge ratio (*m*/*z*) were collected after a 9 min solvent delay. Products were identified through comparison of GC‐MS retention time and mass spectra with commercial standards, enzymatically produced authentic compounds or NMR analysis, as stated.

### Metabolite profiling via LC‐MS analysis

Plant tissue was ground under liquid N_2_ and extracted with hexane:ethyl acetate (70:30 v/v) for 12 h. Extracts were dried‐down and resuspended in methanol before UPLC‐HILIC‐MS/MS analysis. All measurements were carried out on a Q Exactive HF mass spectrometer coupled with a Vanquish LC system (Thermo Scientific, https://www.thermofisher.com/). A total injection volume of 2 μl was separated on a Waters Acquity UPLC BEH Amide column (150 × 2.1 mm; 1.7 μm) coupled to an Acquity UPLC BEH Amide VanGuard pre‐column (5 × 2.1 mm; 1.7 μm). The column was maintained at 45°C at a flow rate of 0.4 ml min^−1^. The mobile phases consisted of: (A) water with 10 mm ammonium formate and 0.125% formic acid and (B) acetonitrile:water (95:5, v/v) with 10 mm ammonium formate and 0.125% formic acid. A 17 min separation was conducted under the following gradient: 0 min 15% B; 0–2 min 100% B; 2–7.7 min 70% B; 7.7–9.5 min 40% B; 9.5–10.25 min 30% B; 10.25–12.75 min 100% B; 12.75–16.75 min 100% B. The Orbitrap MS instrument was operated in positive electrospray ionization (ESI) mode with the following parameters: mass range 60–900 *m*/*z*; spray voltage +3.6 kV; sheath gas (nitrogen) flow rate 60 units; auxiliary gas (nitrogen) flow rate 25 units; capillary temperature 320°C; full scan MS1 mass resolving power 30,000; data‐dependent MSMS (dd‐MSMS) three scans per cycle; dd‐MSMS mass resolving power 15,000. Thermo Xcalibur 4.0.27.19 was used for data acquisition and analysis. MS‐DIAL v.3.70 was used for batch data processing.

### Product purification and NMR analysis

For NMR analysis of enzyme products >1 mg of target compounds was extracted from 12 L *E. coli* cultures co‐expressing the desired enzyme combinations. Products were extracted using hexane and purified by silica chromatography and semi‐preparative HPLC as described previously (Murphy *et al.*, 2019). Purified compounds were resuspended in chloroform‐D spiked with 0.03% TMS as internal standard and used to collect 1D (^1^H, ^13^C) and 2D (COSY, HSQC, HMBC, selective HSQC, selective HMBC and NOESY) spectra on a Brucker Avance 800 MHz spectrometer (https://www.bruker.com/) equipped with a 5 mm CPTCI cryoprobe. The NMR reports were generated using MestReNova and ACDLabs.

### Elicitation of stress responses in *S. italica* tissues

Abiotic stress treatments were conducted using 4‐week‐old *S. italica* plants. To mimic below‐ground oxidative stress in *S. italica*, 10 mm CuSO_4_ in 300 ml of nutrient solution was added to the soil over a period of 48 h. Control plants were treated with nutrient solution only. For drought stress, watering was withheld for seven consecutive days, resulting in 30% relative water content at the time of harvest. Control plants were well watered to ≥ 80% relative water content at time of harvest. For UV irradiation, detached leaf blades were placed on wet paper towels, irradiated for 2 h under a 254 nm UV lamp situated 20 cm from the leaf surface, and then kept in the dark in a high‐humidity incubator at 30°C until sample collection. As a negative control, leaf blades were handled in the same manner, but without UV treatment. Fungal elicitor assays were performed using mycoprotein commercially produced from *F. venenatum* by Quorn^TM^ (https://www.quorn.us/mycoprotein). Here, stems of 10‐week‐old *S. italica* plants were cut lengthwise and incubated by wrapping a mycoprotein‐soaked cheesecloth around the incision, covering the incision with tape to retain moisture and incubating for 48 h (Schmelz *et al.*, 2011). Appropriate controls were treated in the same fashion, but with the cheesecloth soaked with water only.

### Gene expression analysis

The heatmaps and dendograms were generated using R‐statistical software with pheatmap 1.0.12 from CRAN (https://CRAN.R‐project.org/package=pheatmap) with gene expression data obtained from Phytomine (https://phytozome.jgi.doe.gov/phytomine). Quantitative real‐time PCR was performed with total RNA extracted from 100 mg of *S. italica* plant material used in stress assays (see above) using the Plant RNA Purification Kit (Qiagen, https://www.qiagen.com/). Equal amounts of RNA were used for cDNA synthesis with the iScript cDNA Synthesis Kit (Bio‐Rad, https://www.bio‐rad.com/) and oligo(dT) primers. The subsequent qPCR reaction was performed on a Bio‐Rad CFX96 real‐time system using the SsoFast kit and target‐specific oligonucleotides (Table S4). The log fold‐change in gene expression was calculated using the ΔΔC_t_ method with *S. italica* elongation factor 1α (*SiEF1α*) (XM_004984777) (Kumar *et al.*, 2013) as the reference gene and triplicate measurements with three biological replicates. Target specificity was confirmed by sequence verification of representative amplicons. Statistical significance for gene expression was analyzed using the Welch two‐sample *t*‐test (*P*‐value < 0.05).

### Fungal growth‐inhibition assays

Fungal stock cultures of *F. verticillioides* (GL1093) and *F. subglutinans* (M2236) were grown on potato‐dextrose agar plates for 2 weeks under constant light at room temperature (23ºC) before use in bioactivity assays. The antimicrobial efficacy of purified terpene products was assessed following the Clinical and Laboratory Standards Institute M38‐A2 guidelines, as described previously (Mafu *et al.*, 2018). Briefly, fungal growth at 30°C in RPMI 1640 (ThermoFisher, https://www.thermofisher.com/) medium was monitored using a SPECTRAmax 340 Microplate Reader (Molecular Devices, https://www.moleculardevices.com/) through periodic measurements of changes in OD_530_ over 48 h. Each well contained 200 µl of initial fungal inoculum (2.5 × 10^4^ conidia ml^−1^) with 0.4 µl of either pure DMSO or DMSO containing dilutions of HPLC‐purified terpene products, as mentioned above, to yield final concentrations of 2 µg ml^−1^ and 10 µg ml^−1^ per well. The statistical significance for difference in OD_530_ measurements between 0 and 48 h was analyzed using analysis of variance and Tukey honestly significant difference tests (*n* = 4, *P*‐value < 0.05).

## Accession numbers

Accession numbers for all TPS candidates described in this study are given in Table S1.

## Author contributions

PZ conceived the original research and oversaw data analysis; PSK performed most of the experiments; DB assisted with qRT‐PCR; SW assisted with *in vitro* antifungal assays; JD and JM performed synteny analysis; TS and OF assisted with LC‐MS analyses; PSK and PZ wrote the article with contributions from all authors. All authors read and approved the manuscript.

## Conflict of interest

The authors declare that they have no conflicts of interest in accordance with the journal policy.

## Supporting information


**Figure S1. **Protein sequence alignment of select class II diterpene synthases.
**Figure S2. **Protein sequence alignment of select class I diterpene synthases.
**Figure S3. **Sequence similarity matrix of terpene synthase candidates from *Setaria italica* and *Setaria viridis*.
**Figure S4. **Mass spectra of class II diterpene synthase products identified in this study.
**Figure S5. **Mass spectra of class I terpene synthase products identified in this study.
**Figure S6. **Mass spectra of products resulting from co‐expression assays of SiTPS5 and SiTPS13.
**Figure S7. **The NMR analysis of *ent*‐pimara‐8,15‐diene.
**Figure S8. **The NMR analysis of *syn*‐pimara‐7,15‐diene.
**Figure S9. **The NMR analysis of eudesme‐2,11‐diol.
**Figure S10. **Functional analysis of CYP99A17 and CYP99A19.
**Figure S11. **The NMR analysis of abietadien‐19‐ol.
**Figure S12. **The NMR analysis of *syn*‐pimara‐7,15‐dien‐19‐ol.
**Figure S13. **Gene expression analysis of characterized *Setaria italica* terpene synthase genes.
**Figure S14. **Occurrence of terpene synthase and CYP99A17 products in *Setaria italica*.

**Table S1.** Accession numbers and cloned constructs for terpene synthases used in this study.

**Table S2.** Chromosomal location of *Setaria italica* terpene synthase candidate genes.

**Table S3.** Gene synteny analysis of *Setaria italica* terpene synthase candidates.

**Table S4.** Oligonucleotides used for gene expression analysis in this study.
