# Supplementary material for: The foxtail millet (Setaria italica) terpene synthase gene family
Source: Plant J. 2020 May 3;103(2):781–800. doi: 10.1111/tpj.14771 (PMC7497057; doi:10.1111/tpj.14771)
Supplement: Supplementary file 1 — Figure S1. Protein sequence alignment of select class II diterpene synthases. Figure S2. Protein sequence alignment of select class I diterpene synthases. Figure S3. Sequence similarity matrix of terpene synthase candidates from Setaria italica and Setaria viridis. Figure S4. Mass spectra of class II diterpene synthase products identified in this study. Figure S5. Mass spectra of class I terpene synthase products identified in this study. Figure S6. Mass spectra of products resulting from co‐expression assays of SiTPS5 and SiTPS13. Figure S7. The NMR analysis of ent‐pimara‐8,15‐diene. Figure S8. The NMR analysis of syn‐pimara‐7,15‐diene. Figure S9. The NMR analysis of eudesme‐2,11‐diol. Figure S10. Functional analysis of CYP99A17 and CYP99A19. Figure S11. The NMR analysis of abietadien‐19‐ol. Figure S12. The NMR analysis of syn‐pimara‐7,15‐dien‐19‐ol. Figure S13. Gene expression analysis of characterized Setaria italica terpene synthase genes. Figure S14. Occurrence of terpene synthase and CYP99A17 products in Setaria italica. [file TPJ-103-781-s001.zip › tpj14771-sup-0002-FigS2.pdf]

Supplemental Fig. 2: Protein sequence alignment of select class I diterpene synthases.

```
                             20                            40
                              |                             |
SiTPS19    METS------ ---------- ---------- ---------I   5
SiTPS20    MATT------ ---------- ---------- ---------V   5
SiTPS21    MSTTG----- ---------- ---------- -------DPI   8
SiTPS22    M--------- ---------- ---------- ----------   1
SiTPS16    MAICS----- ---------- ---------- -------AAA   8
SiTPS18    MAICS----- ---------- ---------- -------AAA   8
SiTPS26    MAAAA----- ---------- ---------- -------SSL   8
SiTPS27    MAPAA----- ---------- ---------- -------ASL   8
SiTPS36    MQSSP----- ---------- ---------- -------RTI   8
SiTPS14    MPSSS----- ---------- ---------- -------PPA   8
SiTPS15    MASGH----- ---------- ---------- -------EDG   8
SiTPS17    MALT------ ---------- ---------- --------PA   6
SiTPS11    ME-------- ---------- ---------- --------TL   4
SiTPS12    MA-------- ---------- ---------- --------TL   4
SiTPS3     MQGSV----- ---------- ---------- -------MTT   8
SiTPS4     MQSTV----- ---------- ---------- -------MAT   8
SiTPS31    M--------- ---------- ---------- ----------   1
SiTPS32    MAARK----- ---------- ---------- -------AIG   8
SiTPS25    MAPSR----- ---------- ---------- -------VAA   8
SiTPS30    M--------- ---------- ---------- ----------   1
SiTPS38    MALIS----- ---------- ---------- -------AAT   8
SiTPS1     MAAAP----- ---------- ---------- -------ARI   8
SiTPS2     MAAAP----- ---------- ---------- -------ART   8
SiTPS5     M--------- ---------- ---------- ----------   1
SiTPS8     M--------- ---------- ---------- ----------   1
SiTPS13    MHRAV----- ---------- ---------- -------LAA   8
SiTPS28    MAKPV----- ---------- ---------- -------KLT   8
SiTPS29    MPN-I----- ---------- ---------- -------TAA   7
PvKSL13    MPLVT----- ---------- ---------- -------AAA   8
PvKSL15    MPRVT----- ---------- ---------- -------AAA   8
ZmKSL3     MPDVM----- ---------- ---------- -------AAA   8
OsKS1      MQ-------- ---------- ---------- ----------   2
ZmKSL5     MA-------- ---------- ---------- ----------   2
PpCPSKS    MASSTLIQNR SCGVTSSMSS FQIFRGQPLR FPGTRTPAAV  40
```

4.3bits
Sequence logo
0.0bits

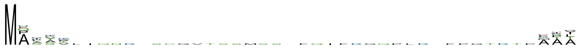

```
                             60                            80
                              |                             |
SiTPS19    LAASLQ---- ---------- ---------- ----------  11
SiTPS20    LATSQS---- ---------- ---------- ----------  11
SiTPS21    LQVSQA---- ---------- ---------- ----------  14
SiTPS22    ---------- ---------- ---------- ----------   1
SiTPS16    TAPATQ---- ---------- ---------- ----------  14
SiTPS18    TAPVTQ---- ---------- ---------- ----------  14
SiTPS26    GTAMQE---- ---------- ---------- ----------  14
SiTPS27    GTAVQA---- ---------- ---------- ----------  14
SiTPS36    NVVGAI---- ---------- ---------- ----------  14
SiTPS14    LLHSSD---- ---------- ---------- ----------  14
SiTPS15    PCSGHE---- ---------- ---------- ----------  14
SiTPS17    VCSVND---- ---------- ---------- ----------  12
SiTPS11    -SFKPQR--- -QST------ ---------- ----------  13
SiTPS12    ISFKPPQ--- -CIS------ ---------- ----------  14
SiTPS3     FSLRPRQ--- -CNG------ ---------- ----------  18
SiTPS4     FALKPGQ--- -CNG------ ---------- ----------  18
SiTPS31    ---------- ---------- ---------- ----------   1
SiTPS32    A--------- ---------- ---------- ----------   9
SiTPS25    VLDGQGM--- -AKA------ ---------- ----------  18
SiTPS30    ---------- ---------- ---------- ----------   1
SiTPS38    SCWKAAP--- ---------- ---------- ----------  15
SiTPS1     VFSFSVE--- -PLL------ ---------- ----------  18
SiTPS2     FSLFSVE--- -PLL------ ---------- ----------  18
SiTPS5     ---------- ---------- ---------- ----------   1
SiTPS8     ---------- ---------- ---------- ----------   1
SiTPS13    RSGDSGG--- -AAGREKLLE LEDRVRE--- ----------  31
SiTPS28    AAGSLPL--- -MKACLSSGG RHLGSLPCGM ATMMLTQ---  41
SiTPS29    APASCSP--- -CGAILASRA PRRGVVKAYP EKRLVAENVR  43
PvKSL13    PASGSPRGVI PLRSSRASCD SRPCAARAYA GKRLVAENTR  48
PvKSL15    PASCSPRGVI PLRSSRASCD SRPCAAGAYA GKRLIAENTR  48
ZmKSL3     LTGFSPRGVI PSRGRASL-- --PGVARAYA ERRLVAENTS  44
OsKS1      ---------- ---------- ---------- ----------   2
ZmKSL5     ---------- ---------- ---------- ----------   2
PpCPSKS    QCLKKRRCLR PTESVLESGS GSYRIVTGPS GINPSSNGHL  80
```

4.3bits
Sequence logo
0.0bits

		100		120	
SiTPS19	----------	----------	----------	----------	11
SiTPS20	----------	----------	----------	----------	11
SiTPS21	----------	----------	----------	----------	14
SiTPS22	----------	----------	----------	----------	1
SiTPS16	----------	----------	----------	----------	14
SiTPS18	----------	----------	----------	----------	14
SiTPS26	----------	----------	----------	----------	14
SiTPS27	----------	----------	----------	----------	14
SiTPS36	----------	----------	----------	----------	14
SiTPS14	----------	----------	----------	----------	14
SiTPS15	----------	----------	----------	----------	14
SiTPS17	----------	----------	----------	----------	12
SiTPS11	----------	----------	----------	----------	13
SiTPS12	----------	----------	----------	----------	14
SiTPS3	----------	----------	----------	----------	18
SiTPS4	----------	----------	----------	----------	18
SiTPS31	----------	----------	----------	----------	1
SiTPS32	----------	----------	----------	----------	9
SiTPS25	----------	----------	----------	----------	18
SiTPS30	----------	----------	----------	----------	1
SiTPS38	----------	----------	----------	----------	15
SiTPS1	----------	----------	----------	----------	18
SiTPS2	----------	----------	----------	----------	18
SiTPS5	----------	----------	----------	----------	1
SiTPS8	----------	----------	----------	----------	1
SiTPS13	----------	----ELLNAG	EPSP------	----------	41
SiTPS28	----------	---RGIASRP	----------	----------	48
SiTPS29	VQNMHRKELE	TRIRNQLQRP	ELPP------	----------	67
PvKSL13	VQNVHRMELE	TRIRNQLLRP	ELPP------	----------	72
PvKSL15	VQNVRRMELE	TRIRNQLLRP	ELPP------	----------	72
ZmKSL3	LPNMHKEQLE	TIIRNQLRKP	QLPP------	----------	68
OsKS1	----HRKELQ	ARTRDQLQTL	ELST------	----------	22
ZmKSL5	----------	----------	----------	----------	2
PpCPSKS	QEGSLTHRLP	IPMEKSIDNF	QSTLYVSDIW	SETLQRTECL	120

4.3bits
Sequence logo
0.0bits

		140		160	
SiTPS19	----------	----------	----------	----------	11
SiTPS20	----------	----------	----------	----------	11
SiTPS21	----------	----------	----------	----------	14
SiTPS22	----------	----------	----------	----------	1
SiTPS16	----------	----------	----------	----------	14
SiTPS18	----------	----------	----------	----------	14
SiTPS26	----------	----------	----------	----------	14
SiTPS27	----------	----------	----------	----------	14
SiTPS36	----------	----------	----------	----------	14
SiTPS14	----------	----------	----------	----------	14
SiTPS15	----------	----------	----------	----------	14
SiTPS17	----------	----------	----------	----------	12
SiTPS11	----------	----------	----------	----------	13
SiTPS12	----------	----------	----------	----------	14
SiTPS3	----------	----------	----------	----------	18
SiTPS4	----------	----------	----------	----------	18
SiTPS31	----------	----------	----------	----------	1
SiTPS32	----------	----------	----------	----------	9
SiTPS25	----------	----------	----------	----------	18
SiTPS30	----------	----------	----------	----------	1
SiTPS38	----------	----------	----------	----------	15
SiTPS1	----------	----------	----------	----------	18
SiTPS2	----------	----------	----------	----------	18
SiTPS5	----------	----------	----------	--------VH	3
SiTPS8	----------	----------	----------	--------MS	3
SiTPS13	----------	----------	--------SS	YDTAWTAMVP	53
SiTPS28	----------	----------	----------	----WVN---	51
SiTPS29	----------	----------	--------SS	YDTAWVSMVP	79
PvKSL13	----------	----------	--------SS	YDTAWVSMVP	84
PvKSL15	----------	----------	--------SS	YDTAWVSMVP	84
ZmKSL3	----------	----------	--------SS	YDTAWVSMVP	80
OsKS1	----------	----------	--------SL	YDTAWVAMVP	34
ZmKSL5	----------	----------	----------	----------	2
PpCPSKS	LQVTENVQMN	EWIEEIRMYF	RNMTLGEISM	YDTAWVARVP	160

4.3bits
Sequence logo
0.0bits

```
                                           180                                   200
                                            |                                     |
SiTPS19     ----------  ----------  ----------  ----------  11
SiTPS20     ----------  ----------  ----------  ----------  11
SiTPS21     ----------  ----------  ----------  ----------  14
SiTPS22     ----------  ----------  ----------  ----------  1
SiTPS16     ----------  ----------  ----------  ----------  14
SiTPS18     ----------  ----------  ----------  ----------  14
SiTPS26     ----------  ----------  ----------  ----------  14
SiTPS27     ----------  ----------  ----------  ----------  14
SiTPS36     ----------  ----------  ----------  ----------  14
SiTPS14     ----------  ----------  ----------  ----------  14
SiTPS15     ----------  ----------  ----------  ----------  14
SiTPS17     ----------  ----------  ----------  ----------  12
SiTPS11     ----------  ----------  ----------  ------S---  14
SiTPS12     ----------  ----------  ----------  ------SNSR  18
SiTPS3      ----------  ----------  ----------  ------SCLL  22
SiTPS4      ----------  ----------  ----------  ------S---  19
SiTPS31     ----------  ----------  ----------  ----------  1
SiTPS32     ----------  ----------  ----------  ----------  9
SiTPS25     ----------  ----------  ----------  ------TDSR  22
SiTPS30     ----------  ----------  ----------  ----------  1
SiTPS38     ----------  ----------  ----------  ----------  15
SiTPS1      ----------  ----------  ----------  ------LQA-  21
SiTPS2      ----------  ----------  ----------  ------LSA-  21
SiTPS5      AKGAPQAPRF  PLFVEWILQN  QHNDGSWG-L  GHLDPCSLGK  42
SiTPS8      TTASLPLARL  ----------  ----------  -------LGR  16
SiTPS13     APGSPLAPRF  PRCVDWILRN  QRADGSWGPA  GDSSEPSLGK  93
SiTPS28     -RDRQQHPSI  SSSSVYYSPP  ATALGS----  --SSSSSLAP  84
SiTPS29     LRGSHQSPCF  PQCVEWILQN  QEDDGSWGV-  -NQFDSSVNK  117
PvKSL13     LRGTHQSPCF  PQCVEWILQN  QQDDGSWGV-  -NQFDSSVNK  122
PvKSL15     LRGSHQSPCF  PQCVEWILQN  QQDDGSWGV-  -NPFDSSVNK  122
ZmKSL3      VRGSHQTPRF  PQCVEWILQN  QQDDGSWGV-  -SQSDSSVSK  118
OsKS1       LRGSRQHPCF  PQCVEWILQN  QQDDGSWGTR  G--FGVAVTR  72
ZmKSL5      ----------  ----------  ----------  ----------  2
PpCPSKS     ALDGSHGPQF  HRSLQWIIDN  QLPDGDWGEP  SLF----LGY  196
```

Sequence logo (4.3bits / 0.0bits)

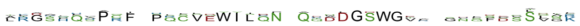

```
                                           220                                   240
                                            |                                     |
SiTPS19     ----------  ----------  ----------  -------SPE  14
SiTPS20     ----------  ----------  ----------  -------SPE  14
SiTPS21     ----------  ----------  ----------  ---------Q  15
SiTPS22     ----------  ----------  ----------  ----------  1
SiTPS16     ----------  ----------  ----------  -------SAA  17
SiTPS18     ----------  ----------  ----------  -------SAA  17
SiTPS26     ----------  ----------  ----------  -------EQR  17
SiTPS27     ----------  ----------  ----------  -------EQR  17
SiTPS36     ----------  ----------  ----------  -------NPV  17
SiTPS14     ----------  ----------  ----------  -------AEG  17
SiTPS15     ----------  ----------  ----------  -------KMK  17
SiTPS17     ----------  ----------  ----------  -------VQG  15
SiTPS11     ----APGSR-  LLGRCRSISG  RWRRRQKYTT  LQQ-CRCQSQ  48
SiTPS12     SCHIAASPRP  LIRRCPSVSR  R-RCLQRRTC  RLR-CG-QSQ  55
SiTPS3      AS--AASQRP  CIS-------  -WRRRQQKHT  LQR----QQM  48
SiTPS4      -CHLAIVLSQ  PIGKRPYRSW  MCRRRQQYDV  L-R-CRVTHQ  56
SiTPS31     ----------  ----------  ----------  ----------  1
SiTPS32     --------NK  ----------  ----------  ----------  11
SiTPS25     IDLVVTTGGV  ----------  ----------  ----------  32
SiTPS30     ----------  ----------  ----------  ----------  1
SiTPS38     ----------  ----------  ----------  ----------  15
SiTPS1      ----------  ----------  ----------  ----------  21
SiTPS2      ----------  ----------  ----------  ----------  21
SiTPS5      DAISSTLSCI  LALKTWNIGD  EHIRKGLCFI  EKN-SSYIMD  81
SiTPS8      AAET------  ---RPQRFQP  VRAQRRVC--  ----------  35
SiTPS13     AALSSTLACV  LALRTFGVGD  EHVGKGLRFI  GHN-SSFVAD  132
SiTPS28     ALLLA-----  VAVRR-----  ---RRAPS--  -----SLLSG  104
SiTPS29     DVLLSTLACV  IALKRWNVGR  ENIRRGLHFI  GRN-FSVAMD  156
PvKSL13     DVLLSTLACV  LALKRWNVGR  ENIWRGLHFI  GRN-FSVAMD  161
PvKSL15     DVLLSTLACV  LALKRWNVGR  ENIWRGLHFI  GRN-FSVAMD  161
ZmKSL3      DVLLSTLACV  LALKRWNVGR  ENIWRGLHFI  GRN-FSVAMD  157
OsKS1       DVLSSTLACV  LALKRWNVGQ  EHIRRGLDFI  GRN-FSIAMD  111
ZmKSL5      ----------  ----------  ----------  ----------  2
PpCPSKS     DRVCNTLACV  IALKTWGVGA  QNVERGIQFL  QSNIYKMEED  236
```

Sequence logo (4.3bits / 0.0bits)

		260		280	
SiTPS19	----------	--------HN	P--RPYTPSL	WGDFF-----	29
SiTPS20	----------	--------HN	P--RPYTPSP	WGDFF-----	29
SiTPS21	----------	--------RK	G--RSYTPSP	WGDFF-----	30
SiTPS22	----------	----------	----------	----------	1
SiTPS16	----------	--------EQ	DCRRQYAPSV	WGDFF-----	34
SiTPS18	----------	--------E-	DCRRQYGPSV	WGDFF-----	33
SiTPS26	----------	--------HK	P--RPYHPSP	WGDFF-----	32
SiTPS27	----------	--------HK	P--RPYHPSP	WGDFF-----	32
SiTPS36	RALLIGAMGT	TCDPVVVVEH	RKPQPYTPSP	WGDFF-----	52
SiTPS14	LRNYTKF---	----------	------HPSI	WGDFF-----	33
SiTPS15	KPATSTF---	----------	------HPTL	WGDFF-----	33
SiTPS17	LRNDRTF---	----------	-----FHPSL	WGDFF-----	32
SiTPS11	QDSSMERARH	HPDEADSRLS	RSTGIFHPSI	WRDFF-----	83
SiTPS12	QDCA-PRTGH	GLDEDDSRLS	QNAGIFHPSI	WGDFF-----	89
SiTPS3	QQRSVATVHH	DNLEVDDRLS	KNPCNFQPSI	WGDFF-----	83
SiTPS4	HPAG--GLHD	DPDEIDDRPR	KNPPRFHPSI	WGDFF-----	89
SiTPS31	----------	----------	----------	----------	1
SiTPS32	----------	----------	ETCGAFEPCL	WGDFF-----	26
SiTPS25	----------	----------	DDDEKFAPSV	WGDFF-----	47
SiTPS30	----------	----------	----------	----------	1
SiTPS38	----------	----------	----EVHPSV	WGDFF-----	26
SiTPS1	----------	----------	------SP-A	AGNGR-----	29
SiTPS2	----------	----------	------SPTA	AARNE-----	30
SiTPS5	EKCSAPLGFN	IIFPGMIRLG	IDLGLEFPLK	QSDVDGIFRL	121
SiTPS8	----------	---AKVVSGG	VDMALE-PKN	TREVEN--RI	59
SiTPS13	DKCDTPAGFN	VIFPGMLARG	LGMGLEIPLA	HGDIDSILRL	172
SiTPS28	RQPKFPRLMA	NTNAAANKLC	--GGRSMPPA	ACCRH-----	137
SiTPS29	EQTTAPIGFN	ITFAGMLRLA	IDMGLEFPIR	QTDVHGILHL	196
PvKSL13	EQTTAPIGFN	ITFATMLSLA	IDMGLEFPIR	QTDVHGILEL	201
PvKSL15	KQTTAPIGFN	ITFATMLSLA	IDMGLEFPLR	QTDVHGILHL	201
ZmKSL3	EQFTSPIGFN	FTFPGLLSLG	IDMGLEFPVR	QIDVCGILHR	197
OsKS1	EQIAAPVGFN	ITFPGMLSLA	MGMDLEFPVR	QTDVDRLLHL	151
ZmKSL5	----------	----------	------KPLN	LTPAFLSLMA	16
PpCPSKS	DANHMPIGFE	IVFPAMMEDA	KALGLDLPYD	ATILQQISAE	276

Sequence logo 4.3bits 0.0bits

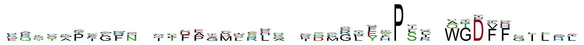

		300		320	
SiTPS19	----------	----------	----------	----------	29
SiTPS20	----------	----------	----------	----------	29
SiTPS21	----------	----------	----------	----------	30
SiTPS22	----------	----------	----------	----------	1
SiTPS16	----------	----------	----------	----------	34
SiTPS18	----------	----------	----------	----------	33
SiTPS26	----------	----------	----------	----------	32
SiTPS27	----------	----------	----------	----------	32
SiTPS36	----------	----------	----------	----------	52
SiTPS14	----------	----------	----------	----------	33
SiTPS15	----------	----------	----------	----------	33
SiTPS17	----------	----------	----------	----------	32
SiTPS11	----------	----------	----------	----------	83
SiTPS12	----------	----------	----------	----------	89
SiTPS3	----------	----------	----------	----------	83
SiTPS4	----------	----------	----------	----------	89
SiTPS31	----------	----------	----------	----------	1
SiTPS32	----------	----------	----------	----------	26
SiTPS25	----------	----------	----------	----------	47
SiTPS30	----------	----------	----------	----------	1
SiTPS38	----------	----------	----------	----------	26
SiTPS1	----------	----------	----------	----------	29
SiTPS2	----------	----------	----------	----------	30
SiTPS5	REIELRQS--	-MALGRKAYM	AYIAEGLADV	QDWDGVLTYQ	158
SiTPS8	RQ-ELLDP--	-KQL------	----------	----------	69
SiTPS13	RDTELK-S--	-MGSGSKTFM	AYVAEGLGSL	LDWDQARAYQ	208
SiTPS28	----------	--SGGQRF--	------LSSL	PCW--MMPST	155
SiTPS29	REMELKRQAV	DSSYGRKAYM	AYIAEGLGNM	LDWDEIMKFQ	236
PvKSL13	REMELKRQAV	YGSYGRKAYM	AYITEGLGNR	LDWDEVMKFQ	241
PvKSL15	REMELKRQAV	YGSCGRKTYM	AYIAEGLGNM	MDWDEVMKFQ	241
ZmKSL3	REMELKRLAV	DSSFGRKAYM	AFIPEGFGNM	LDWDQVMKFQ	237
OsKS1	REIELEREAG	DHSYGRKAYM	AYVTEGLGNL	LEWDEIMMFQ	191
ZmKSL5	TSCRFSSGGH	AHRFASSLPC	GRTTP-----	----------	41
PpCPSKS	REKKMKKIPM	AMVYKYPTTL	LHSLEGLHRE	VDWNKLLQLQ	316

Sequence logo 4.3bits 0.0bits

		340		360	
SiTPS19	----------	LTHQ---PCT	PAELLSMKEK	ARGKEEEVRR	56
SiTPS20	----------	LTHQ---LCT	PAELLSMKEK	AQVKEEEVRR	56
SiTPS21	----------	LHHV---PYT	PSQLLSLKQR	AQIKEEEVRQ	57
SiTPS22	----------	----------	-------KET	ARIKEEEVRK	14
SiTPS16	----------	ISYE---PCT	TEELLSMQEK	ARALKEEVRR	61
SiTPS18	----------	ISYE---PCT	PEELLSMQEK	ARALKEEVRR	60
SiTPS26	----------	LGHQ---PCT	PAELLAMKEE	ARTKEEELRR	59
SiTPS27	----------	LGHQ---PCT	PAELLAMMEE	ARTKEEELRR	59
SiTPS36	----------	LSHQ---PCT	PSQLLSMKER	ARVKEEEVKQ	79
SiTPS14	----------	LTYQ---PPT	APQSESMKER	AGVLREKVRE	60
SiTPS15	----------	LSHK---PPT	SPQETHMRER	AGVLREEVRK	60
SiTPS17	----------	LTYQ---PPT	APKRAYMTER	AEVLKEEVRK	59
SiTPS11	----------	LGYSNPDNSF	QQLQAWMD-R	ADKLKGEVAQ	112
SiTPS12	----------	LGYSNPAASS	QQQQTQMEER	ADKLKEEVAE	119
SiTPS3	----------	LRYSDPAASS	KQ-QIWMAER	ADKMKEAVAK	112
SiTPS4	----------	LHYSNTVACS	PHQQVRMAER	ADKLKEEVAD	119
SiTPS31	----------	----------	----------	----------	1
SiTPS32	----------	VNYT--PTSS	QRSEEWMRER	SEQLKGEVCR	54
SiTPS25	----------	ITYV--PPMS	QRSEEWMRER	AELLKGQVHQ	75
SiTPS30	----------	----------	----------	-DALKEKVRL	10
SiTPS38	----------	INYI--PEPL	QISDEEMLDR	VNQLKGEVSG	54
SiTPS1	----------	RGHRHRSGSI	RSSLA-----	-----YKTPQ	49
SiTPS2	----------	HGGRPRGGSI	RPLVAASKAL	LPISDFDLQQ	60
SiTPS5	SKNGSLFNSP	SA--TAALAI	HGHNANALKY	LEFLVNKLGS	196
SiTPS8	---------P	SSYDTA----	----------	--WLGNKFVS	84
SiTPS13	RKNGSFFNSP	AT--TAAAAI	HNHNGRALDY	LDSLISKFGS	246
SiTPS28	QLAGDLRRRP	AKV-TRVAQV	HK-TLEVEEP	ANSSAKNFSG	193
SiTPS29	RKNGSLFSCP	S-T-TAAALI	HKYNDQALQY	LNLLVSEFGS	274
PvKSL13	RKNGSLFSCP	ST--TAVALI	HKYNDQAHQY	LNSLVSEFGS	279
PvKSL15	RENGSLFCCP	ST--TAGVLI	RKYNDQAHQY	LNSLVSEFGS	279
ZmKSL3	RKNGSLFSTP	ST--TAVALI	HKYNDQALQY	LNLLVNEFGS	275
OsKS1	RKNGSFFNCP	ST--TAATLV	NHYNDKALQY	LNCLVSKFGS	229
ZmKSL5	----------	-TPRTTSSST	RPAAQVSPDR	SKQRDDEAAS	70
PpCPSKS	SENGSF----	LYASTACALM	YTKDVKCFDY	LNQLLIKFDH	352

4.3bits
Sequence logo
0.0bits

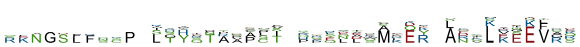

		380		400	
SiTPS19	IMLDAAT---	---TRGLVRK	LDLVDALQRL	GVDYHYKKEI	90
SiTPS20	ILLATAA---	---SPDLVSK	LDLVDVLQRL	GVDYHYKKEI	90
SiTPS21	IILATIA---	---SSDLARK	MELVDTLQTI	GVDYHYKKEI	91
SiTPS22	IIMEIIA---	---SSNLAQK	LELVDTLQRI	GVDYHYK-EI	47
SiTPS16	IVLAA-----	-SNDDDLVRK	LELVDALQRL	GVDYHFKKEI	95
SiTPS18	IVLAAAAAAA	ASDDHDLVRK	LELVDALQRL	GVDYHFKKEI	100
SiTPS26	AVLAAAA---	---SVDLAAK	LELVDALQRI	GVDYRFGEEI	93
SiTPS27	AVLAAAA---	---SPDLAVK	LELVDALQRI	GVDYRFGEEI	93
SiTPS36	IVRDTFAS--	---SSDMALK	LELVDTLQRI	GVGYHYGEEI	114
SiTPS14	ILKS------	---PKELPET	LNLIITLQRL	GLDSYYESEI	91
SiTPS15	IIKG------	---SNDLPEL	LDLIITLQRL	SLDYNYEDEI	91
SiTPS17	MLKG------	---ANEIPKI	LDLIITLERL	GLDNHYENEM	90
SiTPS11	MIVAS-----	-STTGDLHGR	LLLVDVLERL	CLDHLFEEEI	146
SiTPS12	-IMAS-----	-STFSGLHER	LHLIDTLERL	CLDHLFEEEI	152
SiTPS3	MI-AS-----	-SVAWDLHPR	LQLIDALERL	CLVHLFEDDI	145
SiTPS4	MIERR-----	-SSGYSLLQR	LHLIHVLQRL	CLDHLFEDEI	153
SiTPS31	----------	-----NVADT	VRLVDTLERL	GIDNPFVKEV	26
SiTPS32	KFEVG-----	-K-AMSVADT	VRLVDTLERL	GIENHFVKEV	87
SiTPS25	VFNAR-----	-MDAMGVADL	VTYVDTLERL	GLDNHFPEVI	109
SiTPS30	LFE-------	--DSKDIIEQ	MNLVDTIQHL	GIGHHFEKEI	41
SiTPS38	LFE-------	--ACKNVVEK	MDLVDVLQHL	GIDHHFKEQI	85
SiTPS1	KSIVTVQKTL	HGYRKSGRET	MAVVDNLKRL	CIDHYFEEEI	89
SiTPS2	EGLTSIQKIL	QQRRSSAREM	ITTIDNLKRL	CIDHYFEEEI	100
SiTPS5	SAPTIYPSN-	------VHSQ	LCMIDVLQNM	GISYYFAYEK	229
SiTPS8	SA-----LD-	------INSQ	LCLVDTLENM	GISSHFSCEI	112
SiTPS13	SVPTVYPRN-	------MYSQ	LRVVDTLEKM	GISPSFSSEI	279
SiTPS28	GIISTQHNKG	TSSESTIRKQ	LQQVDVLQNM	GISRHFAAEI	233
SiTPS29	AVPAVYPSK-	------IHWQ	LLMVDALEKM	GISQRFVSEI	307
PvKSL13	AVPAVYP---	----SKLHCQ	LLMVDALERM	GISQHFVNEI	312
PvKSL15	AVPAVYP---	----SKLHCQ	LLMVDALERM	GISQHFVNEI	312
ZmKSL3	AVPAMYP---	----SRVHCQ	LSMVDALEKM	GISQRFVSEI	308
OsKS1	AVPTVYPL--	-----NIYCQ	LSWVDALEKM	GISQYFVSEI	262
ZmKSL5	----------	---ETTIRQQ	LQQVDVLENT	GISRHFAGEI	97
PpCPSKS	ACPNVYPV--	-----DLFER	LWMVDRLQRL	GISRYFEREI	385

4.3bits
Sequence logo
0.0bits

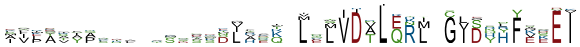

		420		440	
SiTPS19	DELLRAIYDD	-------ED-	G-GSDDLYVT	SLRFYLLRKH	121
SiTPS20	DELLRAIYDD	-------ED-	G-GSDDLYVT	SLRFYLLRKH	121
SiTPS21	DELLCSVYDD	-------KD-	DIGSEDLYIT	SLRFYLLRKQ	123
SiTPS22	ADLLCSIYND	-------KD-	R-GSNDLYIT	SLRFYLLRKH	78
SiTPS16	DDLLLAVYGD	-------ED-	G-GSNDLYVA	SLRFYLLRKH	126
SiTPS18	DVLLLAVYGD	-------ED-	G-GSNDLYVA	SLRFYLLRKH	131
SiTPS26	NGLLRAVVHD	-------ADE	DDDGGDLYLT	SLRFYLLRKN	126
SiTPS27	NGLLGAVVHD	-------ADE	DDGGGDLYLT	SLRFYLLRKH	126
SiTPS36	DDLLRAVHRD	VQA--LHQEG	GCDDDGLYVT	SLRFYLLRKH	152
SiTPS14	DELLHGVYNS	DCY-------	--DEKDLNLV	SLRFYLLRKN	122
SiTPS15	NEILHVVYNS	N-H-------	--SDGDLNIV	SRRFYLLRRS	121
SiTPS17	EEQLRFVYDS	D-Y-------	--NDRDLNLV	SLRFYLLRKN	120
SiTPS11	NAELAQI---	-------EAA	DVRDCDLGTV	ALWFYLLRKH	176
SiTPS12	NAALPQM---	-------QTA	DVSDYDLGTV	ALWFCLLRKH	182
SiTPS3	NAALAQI---	-------KTA	NVTGCDLHTV	ALWFYLLRKH	175
SiTPS4	NGSFTQI---	-------KSA	DLSGCDLQTV	ALWFYLLRNH	183
SiTPS31	EKAIDRVHDE	-------ELD	FGSSNDLHVV	ALRFRLHRQH	59
SiTPS32	EKALEGVHDE	-------ELD	FGSSNDLHVV	ALRFRLLRQH	120
SiTPS25	EAALNRIRTE	-------EPE	PDVFNSLHIV	SLRFRLLRQH	142
SiTPS30	ANTLCNIQHM	-------EFN	---TSSLHEV	SLRFRLLREL	71
SiTPS38	ATTLSSIHRG	-------EFN	---SSSLHEV	ALRFRLLRQH	115
SiTPS1	ESAMGACMDL	----------	-VHSDDLFDA	TLAFRLMREA	118
SiTPS2	ESAMGACMDL	----------	-IHSDDLFDA	TLALRLMREA	129
SiTPS5	NNILDMTYRS	WLQ--YDEEI	---IMDMETC	AMAFRLLRMH	264
SiTPS8	NSTLDMAYRS	LLQ--NDGNI	---TMDMETC	AMAFRLLRMH	147
SiTPS13	NNILEMIYSS	WLA--NDEEI	---MLDMTTC	AMAFRLLRLH	314
SiTPS28	KCILDMTYSC	WLQ--RDEEI	---TLDVETC	AMAFRILRMN	268
SiTPS29	KSILDMTFSR	WLQ--KDEEI	---MMDIATC	AMAFRLLRMN	342
PvKSL13	KSILDMAFSH	WLQ--KDEEI	---MMDIATC	AMTFRLLRMN	347
PvKSL15	KNILDMAFSH	WLQ--KDEEI	---MMDIATC	AMAFRLLRMN	347
ZmKSL3	ESILDMAYNC	WLQ--NDEEL	---MMDIATF	AMAFRLLRMN	343
OsKS1	KSILDTTYVS	WLE--RDEEI	---MLDITTC	AMAFRLLRMN	297
ZmKSL5	KCILDRTYRC	WLE--RHEEI	---MQDTTTC	AMAFRILRMN	132
PpCPSKS	RDCLQYVYRY	WKDCGIGWAS	NSSVQDVDDT	AMAFRLLRTH	425

Sequence logo 4.3bits 0.0bits

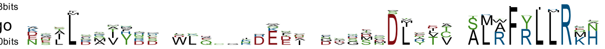

		460		480	
SiTPS19	GYN-VSSD-V	FLKFRDEQ-G	NI-TS--DDV	NCLMTLYDAA	155
SiTPS20	GYN-VSSD-I	FLKFRDEQ-G	NI-TS--DDV	NCLMTLYDAA	155
SiTPS21	GHT-VSSD-V	FQKFRDEQ-G	NI-SS--DDV	TCLLMLYNAA	157
SiTPS22	GYT-VSAD-V	FEKFRDKQ-G	NI-SS--DDV	SCLLMLYDAA	112
SiTPS16	GYT-VSSD-V	FLKFRDEQ-G	HI-SS--DDV	GTLTTLYDAA	160
SiTPS18	GYI-VSSD-V	FLKFRDEQ-G	HI-SS--DDV	GTLTTLYDAA	165
SiTPS26	GFN-VTSD-V	FAKFRDEE-G	NF-AAGDD-V	KCLLMLFDAA	161
SiTPS27	GFN-VTSD-V	FAKFRDEE-G	NF-ATGDDDV	NCLLMLFDAA	162
SiTPS36	GYR-VSSD-V	FVKFKDEQ-G	NF-AS-TDDV	SCFLMLYDAA	187
SiTPS14	GYN-VSSD-V	FLKFITKD-G	NF-VD--ADT	RSLLSLYNAA	156
SiTPS15	GYN-VSSD-V	FLKFKDKH-G	NF-VN--ADT	RSLLSLYNAA	155
SiTPS17	GYD-VPSD-V	FESFKDKE-G	NF-VA--DDT	KSLLSLYNAA	154
SiTPS11	RYC-VSPD-V	FVRFKTVE-G	GF-LG--NNP	IDLLNLYNAA	210
SiTPS12	GYK-VSPD-V	LARFKDED-G	GF-LA--DNP	ADLLSLYNAA	216
SiTPS3	GYR-VSPD-I	FVRFKDQD-G	SF-FA--NNP	VELLSLYNAA	209
SiTPS4	GCR-VSQD-V	FIKFKDEE-G	NF-ES--NSS	EDLLSLYNAA	217
SiTPS31	GF-WVSAD-V	FDKFRDDDTG	SFNINLSNDL	RGLLSLYNAA	97
SiTPS32	GF-WVSAD-V	FDKFRDD-TG	SFNVNLSNDP	RGLLSLYNAA	157
SiTPS25	GI-WVSAD-V	FDKFRDE-AG	SFSTGICSDP	RGLLSLYNAA	179
SiTPS30	VFGYLQVY-E	FNRFKDTN-G	SFNMEMTNDP	RGLLSLYNAA	109
SiTPS38	GF-WVSAG-E	FNKFKHED-G	SFINDITNDP	KGLLSLYNAA	152
SiTPS1	GHDVSAADDV	LRRFTD-DTG	EFKLALSNDI	RGLLSLHDMS	157
SiTPS2	GHDVSAADDV	LRRFTD-DTG	EFKIALSNDI	RGLLSLHDMS	168
SiTPS5	GYD-ISSDAM	SHFADESRFQ	ESLHGHINDT	KTLLKLYKAS	303
SiTPS8	GYD-ISSDVL	SHFAEESRFH	DSVEAHLNDS	KALLELYRAS	186
SiTPS13	GYD-IASEWL	AQFSEESSFH	ESVQGHLNDT	EALLELYKAS	353
SiTPS28	GYN-VSADEL	YDVAQASWFH	PSLEGYLSDT	RSLLELHKAS	307
SiTPS29	GYD-VSSDEL	SHVAEASTFC	DSLQGYLNDT	KSLLELYKAS	381
PvKSL13	GYD-VSSDEL	SHVAEASNFC	DSLQGYLNDT	KSLLELYKAS	386
PvKSL15	GYD-VSSDEL	SHVAEASTFS	DSLQGYLNDT	KSLLELYKAS	386
ZmKSL3	GYD-VSSDEL	SHVAGASTFH	DSLQGYLNDT	KSLLELYKTS	382
OsKS1	GYHVSSVELV	---AEASSFR	ESLQGYLNDK	KSLIELYKAS	334
ZmKSL5	GYD-VSCDLL	YHVVETSGIH	DPLRGHLNDT	RTLLELHKAS	171
PpCPSKS	GFD-VKEDCF	RQFFKDGEFF	CFAGQSSQAV	TGMFNLSRAS	464

Sequence logo 4.3bits 0.0bits

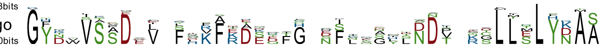

```
                                              500                               520
                                               |                                 |
SiTPS19    HMRT-HGEEI  LDSIIPFNKS  HLQSV-MET-  DLE-----PE  187
SiTPS20    HMRT-HGEKI  LDSIITFNKT  HLQSV-MET-  DLQ-----PE  187
SiTPS21    HLRT-HGEEI  LDSIITFNKI  RLQSL-MTK-  KLE-----PE  189
SiTPS22    YMRT-HGEEI  LDDIITFNKS  RLQFL-MMT-  NLE-----PD  144
SiTPS16    HMRV-HGEDI  LDNIIAFNKS  RLQSLLMKA-  NLD-----PA  193
SiTPS18    HMRV-HGEDI  LDNIIAFNKS  RLQSLLMKA-  NLD-----PA  198
SiTPS26    HLRV-RREEV  LDSAIAFARS  RLESLM--K-  SLE-----PE  192
SiTPS27    HLRV-RGEEV  LDGAIAFARS  RLESLM--K-  SLE-----PE  193
SiTPS36    HLRT-RGEEI  LDSAIAFTKI  RLQSVM--D-  SLE-----PE  218
SiTPS14    YLRT-RDEKV  LDEAISYTTC  RLQDAL--Q-  YLK-----SP  187
SiTPS15    YLPT-HGEAL  LDEAISFTGR  CLQSRL--E-  NLE-----SP  186
SiTPS17    HLRT-HGEEV  LDEAIIFTRS  QLEAVL--D-  SLG-----ST  185
SiTPS11    HLRT-HGEII  LDEAILFTRR  HLEIILPFV-  E-G-----PL  242
SiTPS12    HMRT-HGEII  LDEAILFTRS  CLETTLPNM-  E-E-----SL  248
SiTPS3     HLGT-HGETI  LDEAIVFTRT  HLETIFPSL-  E-G-----SL  241
SiTPS4     YLGT-HGETI  LDDAVSFSKK  CLETTMPHL-  DPE-----GL  250
SiTPS31    HMAV-PGETI  LDDAIVFTRR  HLEAA--KG-  KLT-----SP  128
SiTPS32    HMAV-PGETI  LDDAIAFTRR  HLEAA--KG-  KLT-----SP  188
SiTPS25    HMAV-PGEVA  LDDTIAFARG  HLQAI--KG-  KVR-----SP  210
SiTPS30    YMFI-HDE-A  LEEAISFARL  HLESM--RY-  NLK-----YP  139
SiTPS38    NLLT-HDEGA  LEEALLFARR  HLELM--KS-  SLK-----SP  183
SiTPS1     HLDM-GDEAS  LSKAKEFSSK  HLESAI--R-  YLE-----PG  188
SiTPS2     HLDM-GDEAS  LNKAKEFTRK  HLASAI--R-  YLE-----PG  199
SiTPS5     QVCIYEDECN  LQNIGSWSGK  LLHEQLSSN-  RLSI----PI  338
SiTPS8     LVRILEDEGT  LEKIGAWSGK  LLKQQLCSN-  RLSR----SI  221
SiTPS13    QVQIMEDEAI  LENIGSWSAE  LLKQQQCSN-  KISR----SV  388
SiTPS28    KVSISEDESI  LDTIGSWSGW  LLKEQLRSG-  ALQS----TP  342
SiTPS29    KVSLSENDLI  LDSIGSWSGN  LLNDKLYSN-  RAQK----TP  416
PvKSL13    KVSLSGNDLI  LDSIGSWSGN  LLKDKLCSS-  RVQK----NL  421
PvKSL15    KVSLSGNDSS  LDCVGLWTGN  LLKDKLCSS-  TLQK----TP  421
ZmKSL3     KVTLSENDLI  LDRIGSWSGN  LLKDKMCCS-  RVQK----DS  417
OsKS1      KVSKSENESI  LDSIGSWSGS  LLKESVCSN-  GVKK----AP  369
ZmKSL5     TVSISEDESI  LDSIGLRSST  LLREQMESSG  ALQN----PP  207
PpCPSKS    Q-TLFPGESL  LKKARTFSRN  FLRTKHENNE  CFDKWIITKD  503
```

Sequence logo (4.3bits / 0.0bits)

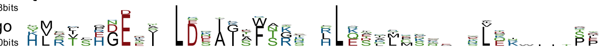

```
                                              540                               560
                                               |                                 |
SiTPS19    LAEEVRFTLE  TTRF-RRVER  VEARHYMSVY  EKKA------  220
SiTPS20    LAEEVRFTLE  TTRF-RQVKR  VEARRYIPVY  EKKA------  220
SiTPS21    LAEEVQCTLE  TPRF-RRVKR  VEARRYISVY  EKKA------  222
SiTPS22    LAEEVRCTLE  TPRF-RRVER  VEARRYISVY  EKKA------  177
SiTPS16    LLEEVRVTLE  TTRF-RRVER  VEARRFISVY  EKKA------  226
SiTPS18    LLEEVRVTLE  TTRF-RRVER  VEARRFISVY  EKKA------  231
SiTPS26    VAEEVRYTLE  TPSF-RRVER  VEARRFISVY  EKKA------  225
SiTPS27    VAEEVRYTLE  TPSF-RRVER  VEARRFISVY  EKKA------  226
SiTPS36    LAREVQCTLE  TPRY-RRVER  VEARRYISVY  ERKA------  251
SiTPS14    LATEVSSSLD  IPLF-KKVGI  IEARNYIPIY  EKES------  220
SiTPS15    LAEEVSCALD  TPLF-RRVGI  LETRNYIPIY  EKEA------  219
SiTPS17    LADEVSLALQ  TPLF-RRIRI  LETRNYIPMY  EKEA------  218
SiTPS11    -AREIKSTLE  IPLP-RRVRI  YESKYYISTY  EKDA------  274
SiTPS12    LAREIKYALE  IPLP-RRVRI  YESKYYISAY  EKDA------  281
SiTPS3     -AHEVKCALE  IPLP-RRVRI  YESKYYISTY  EKEA------  273
SiTPS4     LAREITSALE  IPLP-RRVKI  YELKHYISMY  ETEA------  283
SiTPS31    IKEQVSHALD  IPLP-RFMRQ  LETMHYITEY  EKEE------  161
SiTPS32    IKEQVSRALE  IPLP-RFMRQ  LETMHYLTEY  EKEE------  221
SiTPS25    IAEQISRALD  IALP-RFTRR  LETMHYIAEY  EHEE------  243
SiTPS30    LSEQVNRALH  IPLP-RTVRR  VETLHYMSEY  KHER------  172
SiTPS38    LAEQVERALK  IPLP-RTLKR  VEAVSYIPEY  SGEQ------  216
SiTPS1     LARYVRQSLD  HPYH-LSLMQ  YKARHHLSYL  QTLP------  221
SiTPS2     LACYVRQSLD  HPYH-LSLMQ  YKARHHLSYL  QTLP------  232
SiTPS5     MRHEVEGALK  FPFHLATVGP  LEHKRNIEHF  NTKGIRMQKS  378
SiTPS8     SPKEVEYALK  TPFYSATLEP  LQHKMNIERF  NTKGIQMQKS  261
SiTPS13    DPAEVEHVLK  FPFY-ATLDR  LEHRWNIEHF  KAGGFQMLKS  427
SiTPS28    LFREVEHALE  CPFY-TTLDR  LDHRRNIENF  DATGHQMLKT  381
SiTPS29    IFGEMEYVVK  FPFY-ATLER  LEHKRNIEHF  DAWGSLMVST  455
PvKSL13    IFGEIDYAVK  FPFY-ATLER  LEHKRNIEHF  DAWGSLMLTT  460
PvKSL15    IFGEIEYALK  FPFH-ATLER  LEHKRNIEYF  DAWGSLMLTT  460
ZmKSL3     IFGEIEYAVN  FPLY-STLER  LEHKRNIEHF  DAWGSLMLTT  456
OsKS1      IFEEMKYALK  FPFY-TTLDR  LDHKRNIERF  DAKDSQMLKT  408
ZmKSL5     LFREVEHALY  HPFY-ATLDR  LTHRWNIENF  NTTEHQMLDT  246
PpCPSKS    LAGEVEYNLT  FPWY-ASLPR  LEHRTYLDQY  GIDDIWIGKS  542
```

Sequence logo (4.3bits / 0.0bits)

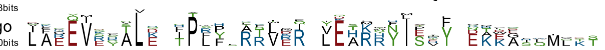

		580		600	
SiTPS19	---T--R-NE	AILEFAKLDY	NILQALYCEE	LKELTIWWKD	254
SiTPS20	---T--R-NE	AILEFAKLDY	NILQVLYCEE	LKELTIWWMD	254
SiTPS21	---T--R-DE	TILEFAKMDY	NILQAIYCDE	LKELTIWWKD	256
SiTPS22	---V--Q-HK	TLLDFAKLDY	NILQAIYCDE	LKELTIWWKD	211
SiTPS16	---M--R-DD	TILEFAKLDY	NIVQVVYGNE	LKELTIWWKD	260
SiTPS18	---V--R-DD	TILEFAKLDY	NIVQVVYCNE	LKELTIWWKD	265
SiTPS26	---A--R-DE	TMLEFAKLDY	NILQTIYCEE	LKALTMWWKN	259
SiTPS27	---T--R-DE	TLLEFAKLDY	NILQTIYCEE	LKALTMWWKN	260
SiTPS36	---APSR-ND	TILEFAKLDY	NILQALYCEE	LKALTIWWKD	287
SiTPS14	---T--R-NE	VVLKFAKLNF	NLQQLDFCQE	IKECMMWWKD	254
SiTPS15	---T--R-NE	AILEFAKLNF	NLLQLIYCEE	LKEVTMWWKK	253
SiTPS17	---A--R-NE	VILEFAKLNF	NLLQLLYCEE	LKTVTLWWKQ	252
SiTPS11	---TL---HD	KVLQLAKLNS	DIMQLHHQQE	LSILTRWWED	308
SiTPS12	---TV---HE	TILQLAKLNS	NIMQLHHQRE	LITITRWWKD	315
SiTPS3	---TV---HE	SVLQLAKLNS	NIMQLHHQQE	LELITRWWKD	307
SiTPS4	---TV---HE	TILELAKLNS	NLMQLHHQRE	LKIITRWWKN	317
SiTPS31	-----PH-DI	MMLELAKINL	NLLRSVHLKE	LKDLSLWWRD	195
SiTPS32	-----PH-DI	MMLELAKINL	NLLRSVHLKE	LKDLSLWWRD	255
SiTPS25	-----PH-DS	LLLELARLNY	NLVRTLHLKE	LKDLSLWWRD	277
SiTPS30	-----EY-NP	NILELAKIDF	NNLQQLHIKE	IEAISKWWKN	206
SiTPS38	-----RY-NP	AILELAKLDF	NLLQRLHQKE	LKTISQWWKD	250
SiTPS1	---T--R-NT	AMEELAISEF	QLNKRLHQKE	MQEVKRWWTD	255
SiTPS2	---T--R-DT	AMEELAISEF	QLNKRLHQKE	MQEVKGWWMD	266
SiTPS5	AFLACQA-AE	DILALATQKF	HISQSLYKKE	LGCIERWAKE	417
SiTPS8	SYLACYA-TE	DILALATEDF	HAAQSIYQQQ	LHHIETWAKE	300
SiTPS13	AYRAYHA-DE	EIVALAADEF	HSSQALYQQE	L----QTSRE	462
SiTPS28	SYLSCYN-NN	DILALGVRDF	SASQFTYQEE	LRHIDSWVKE	420
SiTPS29	KCSSFRV-NQ	EFLALAVEDF	SFSQSVYQDE	LQHLDSWVKE	494
PvKSL13	KGSSFHV-NQ	EFLALAVNDF	SLSQSVYQDE	LQHLDSWVKE	499
PvKSL15	KCSSFHV-NQ	EFLALAVKDF	SFSQSVYQDE	LQHLDSWVKE	499
ZmKSL3	KSSSFRI-NQ	EFLALAVEDF	SFSQRVYRDE	LRHLDSWVKE	495
OsKS1	EYLLPHA-NQ	DILALAVEDF	SSSQSIYQDE	LNYLECWVKD	447
ZmKSL5	PYLSSRYTSR	DILALGIRDF	NSSQLTYQQE	LQHLKSWVKE	286
PpCPSKS	LYKMPAVTNE	VFLKLAKADF	NMCQALHKKE	LEQVIKWNAS	582

Sequence logo

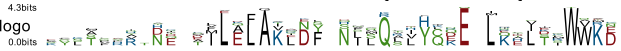

		620		640	
SiTPS19	FKSRTDLRFA	RDRLVEMYFW	MMGVVYE---	--------PY	283
SiTPS20	FKSRTDLRFA	RDRLVEMYFW	MMGVVYE---	--------PY	283
SiTPS21	FQLGTDLRFT	RDRMVELHFW	MMGVVYE---	--------PY	285
SiTPS22	FQSRTDLSFA	RDSMVEMHFW	ILGALYE---	--------PY	240
SiTPS16	LRSRVDLTFS	RDRLVEMHFC	MMGIVYE---	--------PY	289
SiTPS18	LRSRVDLTFS	RDRLVEMHFW	MMGIVYE---	--------PH	294
SiTPS26	FQSVTDLGFA	RDRLAEVHFW	MTGVFYE---	--------PY	288
SiTPS27	FQSVTDLGFA	RDRMLEMHFW	MMGVCYE---	--------PY	289
SiTPS36	LRSRADLRFA	RERVVEMYFW	MLGVVHE---	--------PQ	316
SiTPS14	IHAKSKLSFV	RDRIVELYFW	MNNACYY---	--------PT	283
SiTPS15	LNVEANFHFV	RNRIVEMYFW	MNGACHE---	--------PQ	282
SiTPS17	LNAEANCSFI	RDRIVEMHFW	MTGACSE---	--------AQ	281
SiTPS11	IRMESMIPFA	RDRIVECFLW	ILGVYFE---	--------PC	337
SiTPS12	LHIESRLPFA	RDRVVECYLW	MLGVYFE---	--------PC	344
SiTPS3	LEIESKLPFA	RDRVVECYFW	ILGVYFE---	--------PC	336
SiTPS4	IQLQSNLSFA	RDRIVECYFW	MAGAYFE---	--------PC	346
SiTPS31	LYDSVKLTYC	RDRIVESYFY	SFGVFHG---	--------EE	224
SiTPS32	LYDSVKLTYC	RDRIVESYFY	SFGVFHG---	--------EE	284
SiTPS25	LYDTVKLPYA	RDRMVEIYFW	TCGMLHE---	--------EE	306
SiTPS30	LYAEVALSFA	RNRIVQLYLW	TYAVYYE---	--------HE	235
SiTPS38	LSEDIKLEYV	RDRIVECYFW	AYSVYYE---	--------QE	279
SiTPS1	LGLSREIPVV	RDQVLKWYMW	PMTTFQG---	--------PS	284
SiTPS2	LGLFHEIPVV	RDQVLKWYMW	TTATFQGYSL	SSFQKRKGSS	306
SiTPS5	AGLN-RFKFT	RAVSLDLPIF	MASTIF----	-------PPE	445
SiTPS8	FGLD-KLKYA	RVMSWDVFVF	MASTVF----	-------PPE	328
SiTPS13	VRLD-ELEFA	RIMPLHTLFS	AASTMF----	-------AAG	490
SiTPS28	SRLD-QLPFA	RQKLAYFYLS	AAGTIF----	-------TPE	448
SiTPS29	NKLD-QLQFA	RQKLTYCYLS	AAATIF----	-------PSE	522
PvKSL13	NKLD-QLQFA	RQKLTYCYLS	AAATIF----	-------PSE	527
PvKSL15	NKLD-QLQFA	RQKLTYCYLS	AAATIF----	-------PSE	527
ZmKSL3	NKLD-QLQFA	RQKLTYCYLS	AAATVFS---	--------SE	523
OsKS1	EKLD-QLPFA	RQKLTYCYLS	AAATIF----	-------PRE	475
ZmKSL5	CRLD-QLPFV	RQNLAYFLLS	AAGCMYS---	--------PE	314
PpCPSKS	CQFR-DLEFA	RQKSVECYFA	GAATMFE---	--------PE	610

Sequence logo

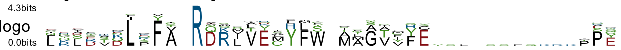

		660		680	
SiTPS19	YSYSRIMLTK	LVMSVSLFDD	LYDNYSTTEE	SQIFTTAME-	322
SiTPS20	YSYSRIMLTK	LVMSVSLLDD	LYDNYSTTEE	SQIFTTAME-	322
SiTPS21	YSYSRIMLTK	FTIYATMFDD	LYDNYGTTKE	SNIFTTAME-	324
SiTPS22	YSYSRTMLTK	FTLLASLLDD	LYDNYSTTEE	SNVFTTAME-	279
SiTPS16	YSYARIMLTK	QVLFLVLLDD	IYDNYSSTEE	SNMFTSALE-	328
SiTPS18	YSYARIMLTK	QVLFMALLDD	IYDNYSSTEE	SNIFTSALE-	333
SiTPS26	YSYSRIMLTK	LVMLASLFDD	FYDNYSTTEE	SNVFTAALE-	327
SiTPS27	YSYSRIMLTK	LITLASLFDD	FYDNYSTTEE	SNVFTAALE-	328
SiTPS36	YSYSRILLTK	FFKLVSLIDD	FCDSYSTTEE	SEEFTMAIEK	356
SiTPS14	CSLSRIVLTK	TTAIVTILDD	IFDTYGTNEE	CMQIAEAI--	321
SiTPS15	YSHSRIILAK	IMGFITILDD	FIDTYATTEE	SMQLGEAI--	320
SiTPS17	YSLSRVITTK	MTAYITILDD	IMDTYSTTEE	AMLLAEAI--	319
SiTPS11	HSRARIILTM	IIAIVTLLDD	IYDSYGSPKE	CDILTNCIE-	376
SiTPS12	YSRSRIIVTM	IIAIVTLLDD	IYDSYATPEE	CELLTKCIE-	383
SiTPS3	YSRGRIILTM	VISIATIFDD	MFDSYGTVEE	CELLTNCIE-	375
SiTPS4	YSRARIILTQ	VMAIISIVDD	IYDVYGTSEE	CELFTRCIE-	385
SiTPS31	ISAARIILTK	VFGLLVLIDD	TFDVRATFEE	SQMLDDALQ-	263
SiTPS32	SSAARIILTK	VFGLLVLIDD	TFDVRATFEE	SQMLDDALQ-	323
SiTPS25	YSRSRMLFAK	TFGMVSLLDD	TFDVHATLDE	CHKLNEAMQ-	345
SiTPS30	YSCARIILTK	LFAVITMMDD	IYDTRATLEE	SQKLNEAIK-	274
SiTPS38	YARARMILAR	LFVLTSLLDD	TYDDHATLEE	CRELTKAIE-	318
SiTPS1	SSRYRVETTK	IISLVYVVDD	IFDLVGTPEE	LSLFTEAIKM	324
SiTPS2	LSRYRIETTK	IIALIYVVDD	IFDLVGTPQE	LSLFTEAIKI	346
SiTPS5	LYHASIAWIQ	NSILTTIVDD	FFDGGGSTEE	LENLVTLIE-	484
SiTPS8	QYDASIAWIQ	NSILTVIADD	FFDDGGSIEE	LKNFIDLIE-	367
SiTPS13	LSEARMAWSK	MCVLTTAMDD	LFDTVGSKEE	LENLVTLID-	529
SiTPS28	LSDARILWAK	NGVLTTVVDD	FFDVGGSTEE	LENLVTLVE-	487
SiTPS29	LSDARISWAK	NGVLTTVVDD	FFDVGGSKEE	LENLIALVE-	561
PvKSL13	LSDARISWAK	NGVLTTVVDD	FFDVGGSKAE	LENLIELVE-	566
PvKSL15	LSDARISWAK	NGVLTTVVDD	FFDVGGSKEE	LENLIELVE-	566
ZmKSL3	LSDARISWAK	NGVLTTVVDD	FFDVGGSKEE	LENLIALVE-	562
OsKS1	LSEARIAWAK	NGVLTTVVDD	FFDLGGSKEE	LENLIALVE-	514
ZmKSL5	LSEARTLCAK	NGALITVVDD	FFDVGGSKEE	LENLVMLVEL	354
PpCPSKS	MVQARLVWAR	CCVLTTVLDD	YFDHGTPVEE	LRVFVQAVR-	649

Sequence logo 4.3bits 0.0bits
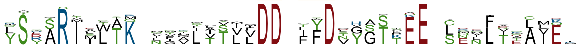

		700		720	
SiTPS19	----------	----------	----------	--KWDEKAAE	330
SiTPS20	----------	----------	----------	--RWDEKAAE	330
SiTPS21	----------	----------	----------	--RWDEQITE	332
SiTPS22	----------	----------	----------	--RWDGQTTE	287
SiTPS16	----------	----------	----------	--RWDEKAAE	336
SiTPS18	----------	----------	----------	--RWDEKAAE	341
SiTPS26	----------	----------	----------	--RWDEQATD	335
SiTPS27	----------	----------	----------	--RWDEQATE	336
SiTPS36	LALTLILETA	CAMHKKLTII	LTHLHLLLQI	DGRWNEEVAE	396
SiTPS14	----------	----------	----------	-YRWDESAVH	330
SiTPS15	----------	----------	----------	-FRWDKNATT	329
SiTPS17	----------	----------	----------	-YRCEENAAE	328
SiTPS11	----------	----------	----------	--RWDPKAAY	384
SiTPS12	----------	----------	----------	--SWDAKAAH	391
SiTPS3	----------	----------	----------	--RWDRKLAD	383
SiTPS4	----------	----------	----------	--SWDTKVGQ	393
SiTPS31	----------	----------	----------	--RWDESMVS	271
SiTPS32	----------	----------	----------	--RWDESTVS	331
SiTPS25	----------	----------	----------	--RWDESEVS	353
SiTPS30	----------	----------	----------	--RWDESAVS	282
SiTPS38	----------	----------	----------	--RWDESDIS	326
SiTPS1	----------	----------	----------	---WNTVAAD	331
SiTPS2	----------	----------	----------	---WNTEAAD	353
SiTPS5	----------	----------	----------	--KWDAHAGI	492
SiTPS8	----------	----------	----------	--RWDADAGI	375
SiTPS13	----------	----------	----------	--MWDAHEEA	537
SiTPS28	----------	----------	----------	--MWDEHHKI	495
SiTPS29	----------	----------	----------	--KWHEHHAD	569
PvKSL13	----------	----------	----------	--KWHEHHAD	574
PvKSL15	----------	----------	----------	--KWHEHHAD	574
ZmKSL3	----------	----------	----------	--KWHGHHAV	570
OsKS1	----------	----------	----------	--KWDGHQ-E	521
ZmKSL5	----------	----------	----------	---WDEHRKI	361
PpCPSKS	----------	----------	----------	--TWNPELIN	657

Sequence logo 4.3bits 0.0bits
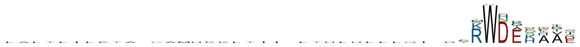

		740		760	
SiTPS19	KF-PAYLRAL	YKNILGTTDE	IVEELKLQNN	KH-AELVKKL	368
SiTPS20	KF-PSYLRAL	YKNIIGTTDE	IVEELKLQNN	KH-AELVRKL	368
SiTPS21	QL-PAHLKAL	FINILNSTNK	MEEELKFQKN	KH-AELIKEL	370
SiTPS22	KF-PAHMKAL	LINILNTTNK	IEDELKLQKN	RH-AELVKKL	325
SiTPS16	QI-PEYLRPF	FTNVIRCTDK	VIGELKLQNN	KH-AEVVKEM	374
SiTPS18	QI-PEYLRPF	FTNVIRCTDK	VTGELKLQNN	KH-AEVVKEM	379
SiTPS26	QL-PEHLREF	YLNTLSSTNG	IEEDLKFQNN	KH-AELVKEL	373
SiTPS27	QL-PAYLREF	YLNTLSSTNG	IEEDLKFQNN	KH-AELVKEL	374
SiTPS36	QI-PAHMKAL	YINILDNIND	IIHELKLGEN	SH-AELAKEL	434
SiTPS14	LL-PEYVKGF	YLCLLETFIS	FEDELGPEKS	YL-VFYLKER	368
SiTPS15	LL-PEYTRDF	YLYLLNTFCS	FEEELGTGKS	YR-VFYLKEA	367
SiTPS17	LL-PEYMKNF	YFHLLKTFDS	FENELGPNKS	FR-VFYLKEL	366
SiTPS11	DL-PECMRIA	LGKILDSYET	IENMLHQEEK	HR-ILYLRYF	422
SiTPS12	DL-PECLKFA	LGKMLDSFQT	IANMLHQEEK	YR-MSYLRYF	429
SiTPS3	DL-PECMKHA	LGKILDSYET	MDNELAQEEK	YR-MPYLKNF	421
SiTPS4	DL-PANLKII	LECIFDTYKD	IEHELETEQK	YR-LSYLKFV	431
SiTPS31	LL-PEYLRMF	YIKTLSNFNE	IEDTLEPYEK	YR-MAYVKKE	309
SiTPS32	LL-PQYLRMF	YIKTLSNFNE	IEDTLEPHEK	YR-MDYVKKE	369
SiTPS25	IL-PEYLRLL	YIKTLSNFKE	FEDSLERDQK	YR-MSYAKKA	391
SiTPS30	IL-PEYLKKF	YLRILRTFKE	IQDMLAPNEQ	YK-VSYAQKS	320
SiTPS38	LL-PEYIKKL	FLKVIRNFKE	FEDELEAHEK	YR-IAYARKG	364
SiTPS1	SL-PSGIRSC	YKAIYTTTNE	IADMVEEERG	FNPINHLRHA	370
SiTPS2	LL-PSGIRSC	YKTIYTSTNE	IANMVEEEHG	FNPVNHLKNS	392
SiTPS5	GFCSEDVEIM	FYAIYNTNNQ	IGAKAAEVQN	RRVIGHIAKV	532
SiTPS8	EFCSEDVEIL	FRAVYDTNNQ	IAAKGAVVQN	RRVVDHIAQV	415
SiTPS13	GFCSERVEIV	FRAIYETSNQ	VGAQAAVVHN	RSVIGHITEL	577
SiTPS28	EFYSEHVEIV	FSAIYTSVNE	LGEKASLLQD	RDVTKHLVEI	535
SiTPS29	KFYSEQVKIV	FSAIYATTNQ	LGAKASAAQG	RDVTKHLAKI	609
PvKSL13	KYYSEQVRII	FSAIYATTNQ	LGAKASAAQD	RDVTKHLAEI	614
PvKSL15	KYYSEQVRTV	FSAIYATTNQ	LGAKASAAQG	RDVTKHLAEI	614
ZmKSL3	EFYSEQVKIV	FSAIYTTVNH	LGAMASAAQG	RDLTNHLVEI	610
OsKS1	EFYSEQVRIV	FSAIYTTVNQ	LGAKASALQG	RDVTKHLTEI	561
ZmKSL5	EFYSEHVEII	FSSIYNSVNQ	YGAKASLLQD	RNVTKHLVQI	401
PpCPSKS	GL-PEQAKIL	FMGLYKTVNT	IAEEAFMAQK	RDVHHHLKHY	696

Sequence logo (4.3bits / 0.0bits)

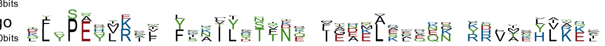

		780		800	
SiTPS19	VIDIAKCYHA	EVKWRDENYV	PTNIDEHLQI	SVGTSACMHI	408
SiTPS20	IIDVAKCYHA	ELKWRDENYV	PTNVDEHLQI	SVCSSACMHV	408
SiTPS21	VIHTAKSYHA	EVKWRDEHYI	PTNVEEHLQI	SMSSSVCMQI	410
SiTPS22	VICTAKFYHA	EVNWRDQRYV	PTSVDEHLQI	SMRSSVCMQI	365
SiTPS16	ALHVTKSYHA	EVTWRDEHYV	PADVDEHLQI	SLGSVTAMQV	414
SiTPS18	ALHVAKSYHA	EVTWRDEHYV	PADVDEHLQI	SLGSIAAMQT	419
SiTPS26	VIDLAKNYRA	EVKWRDEHYV	PTKVEEHLQL	SVPSSGCMQI	413
SiTPS27	AIDLAKNYNA	EVKWRDERYV	PTKVEEHLQL	SVPSSGCMQI	414
SiTPS36	LIDIAQCYHA	EVKWRDEHYV	PAKVEEHLQL	SAPSSACMHI	474
SiTPS14	MKHLVHLYCK	ELKWREENYV	P-SMSEHLQV	TMESVGSAAL	407
SiTPS15	LKQLVQAYID	ELKWRDENYI	PETLSEHLGL	SMRSSGGSPI	407
SiTPS17	LKILVRGGSQ	EVKWRDEHYV	PKTINEHLEM	SRATVGGFQV	406
SiTPS11	TEDLVRSFNM	EVKMLQEGYI	PKSVEEHLKV	SLRTGGCPIL	462
SiTPS12	IEDLVRSFSM	EVKMLEEGYI	PESVEEHLQV	SIRTGGCPLL	469
SiTPS3	TIDLVRGYNA	EVKMREEGYI	PRTVNEHLQV	SLRTGACHLL	461
SiTPS4	TIDWVRAYTT	EVKWRDQRYV	PATVEEHLQL	SVRSGACHLL	471
SiTPS31	YPLQSKNSIQ	QAKWFNENCT	P-SFKEHLDV	SLMATGLPLL	348
SiTPS32	YKLQSKNSIQ	QAKWFNENCT	P-SFKEHLDV	SLMATGLPLL	408
SiTPS25	YKLSSKNYLR	EAIWSSQKYQ	P-SFKEHEEV	SIMSSGLPML	430
SiTPS30	FQTLSNYYLQ	GAEWFHCKKT	P-TFKERVEA	SLMDSGGSFS	359
SiTPS38	FQLISKSYLQ	EAEWSHHNYI	P-SFNDHVNV	STISAGAQLL	403
SiTPS1	WAVLFDGFMV	EARWLATDQV	P-TAEDYLRN	GAVTSGVPLT	409
SiTPS2	WAVLFDGFMI	EARWLATDQV	P-TAEDYLRN	GAVTSGVPLT	431
SiTPS5	WVNAVRANMT	EAEWTRKRYV	P-TMQEYMPV	AEVTMVLGPI	571
SiTPS8	WLALVRAYMV	EADWARTRHV	P-TMEEYMAV	AEVSIALGPV	454
SiTPS13	WAEAATAMMA	EAEWRMTGHV	P-SMEEYMAV	ALPSFALGPI	616
SiTPS28	WLDLLRSMMT	EVEWRTSSYV	P-TAEEYITN	AALTFALGPI	574
SiTPS29	WLDLLRSMMT	EAEWQRSQHV	T-TVEEYMTN	AVVSFALGPI	648
PvKSL13	WLDVLRSMMM	EAEWQRSQHV	P-TVEEYMTN	AVVSFALGPI	653
PvKSL15	WLDLLRSMMM	EAEWQRSQHV	P-TVEEYMTN	AVVSFALGPI	653
ZmKSL3	WLDLLRSMMV	EAEWQRCQYV	P-TVEEYMTN	AVVSFALGPI	649
OsKS1	WLCLMRANMT	EAEWQRTKYV	P-TMEEYMAN	AVVSFALGPI	600
ZmKSL5	WLDLLKSMMT	EVEWRLNKYV	P-TEEEYMTN	ASLTFALGPI	440
PpCPSKS	WDKLITSALK	EAEWAESGYV	P-TFDEYMEV	AEISVALEPI	735

Sequence logo (4.3bits / 0.0bits)

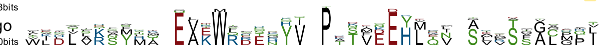

```
                                    820                    840
                                     |                      |
SiTPS19   TNLAFISLGD MTT--KEAIE WAFTYPKIIR GVCIVGRIGN 446
SiTPS20   TNLAFISLGD RAS--KEAVE WAFTYPKIIR GVCIVARIVN 446
SiTPS21   TNLVLISLGD VTT--REDVN WAFTFPKIIR GACIVGRVGN 448
SiTPS22   INLVLISLRE ETTTREEDVD WVFTFPKIVR GVSIVGRIGN 405
SiTPS16   VVLTFVSLGD VTS--REAID WALTYPKIVR GVTVIARIMN 452
SiTPS18   VVLTFVSLGD VTT--REAID WALTYTKIVR GVTVIARIMN 457
SiTPS26   ASFALISMGD VAT--SEAIR WTRTYPKIVR GLCIIGRVMN 451
SiTPS27   TAFALISMGE VAT--SEAIE WTRTYPKIVR GVCIIGRVMN 452
SiTPS36   TNLTFISLGD VTT--REDIQ WVSSYPKIIR GVCTIARISN 512
SiTPS14   TCAAFVGMGD IIT--KEVFE WILSYPQFFK SFAIFVRLAN 445
SiTPS15   LCASLVGMGE IVT--RETLD WFLSYPHLVR SFDTFVRLSD 445
SiTPS17   ACSSFIGMGD IIT--KEILD WLLTYPELLK CFSTIARLAN 444
SiTPS11   SCASFVGMHD IAT--TDFFN WVSSVPKMVQ ALSIILRLVD 500
SiTPS12   SCASFIGMND VAT--KDCFN WVSSVPNMVK ALSRILRLLD 507
SiTPS3    ACASFVGMDE IAT--KDSFD WVSTMPKIVK ALCIILRLLD 499
SiTPS4    SCASFVGMGD IAT--RESFE WVSSMPEIVH SLCIILRLLD 509
SiTPS31   FFTALMSAGQ VIN--NEAFE WALDMPDMFC ANIE------ 380
SiTPS32   FFTALMSAGQ VIS--NEAFE WALDMPDMFY ANIEVGRFLN 446
SiTPS25   TIITLMGYGD VAT--QEVFE WVDAVPEMVR AGSQVTRFLN 468
SiTPS30   CVALLVGMGD IAS--KEALD WALGCTGAVR ACGLITRYMN 397
SiTPS38   CVGLLVGMGD VAT--KEAFE WTIGNNDAIR ACAEVFRFMD 441
SiTPS1    LALVFAMLG- -YDQSNEAAE LADHIPSIIS CPAKILRLWD 447
SiTPS2    LAHIFAMLG- -YDQSNEAAE LADHIPSIIS CPAKILRLWD 469
SiTPS5    VSPSLYLVGP ELS---EDIV RGSEYKDLLR HASICCRLLN 608
SiTPS8    VAPSIYLVGP EIS---EDMV RGPEYKDLLR HMSITIRLLN 491
SiTPS13   VPVSAYLVGP ELP---EAAV RCPEYGELFR HMSVCGRLVN 653
SiTPS28   VLPALYFVGP KIP---EPVV EDPEYNELFR LMSTCGRLLN 611
SiTPS29   VLPALYFVGE ELS---EHAV KDQEYNKLFR LMSTCGRLLN 685
PvKSL13   VLPALYFVGQ ELL---EHAV KDQEYDKLFR LMSSCGRLLN 690
PvKSL15   VLPALYFVGE ELL---EHAV KDQEYEKLFR LMSTCGRLLN 690
ZmKSL3    VLPALYFVGQ ELL---EHAV KDEEYDKLFR LVSTCGRLLN 686
OsKS1     VLPTLYFVGP KL---QEDVV RDHEYNELFR LMSTCGRLLN 637
ZmKSL5    VLPALYFVGP KIP---ESIV KDPEYDELFK LMSTCGRLLN 477
PpCPSKS   VCSTLFFAGH RLD---EDVL DSYDYHLVMH LVNRVGRILN 772
```

Sequence logo 4.3bits 0.0bits

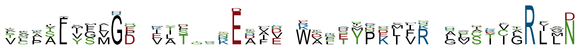

```
                                    860                    880
                                     |                      |
SiTPS19   DIVSHER-EQ ASDHVASTVQ TCIKQYG--- --VTVQEANE 480
SiTPS20   DIMSHER-EQ ASNHVASTVQ TCIKQYG--- --VTVQEANE 480
SiTPS21   DIVSHER-EQ ASEHVVSTVQ TCMNQYG--- --VTMEEANE 482
SiTPS22   DIVSHERREQ ASIHVVSTVQ TCMKQYG--- --VTAEQTKE 440
SiTPS16   DIMSHER-EQ ASDHMTSTVQ TCMKQYG--- --VTVEEAIE 486
SiTPS18   DIMSHER-EQ ASDHMASTVQ TCMKQYG--- --VTVEEAIE 491
SiTPS26   DIVSHER-EQ ASDHVASTVQ TCMKEYG--- --FTVSQANK 485
SiTPS27   DIVSHER-EQ TSDHVVSTVQ TCMKEYG--- --FTVAQANK 486
SiTPS36   DIMSHER-EQ ASKHVVSTVQ TCMKEYG--- --FAPEQAKG 546
SiTPS14   DLVSTQR-EQ TGDHSPSSIQ CYMKEHG--- --TTMQDAYK 479
SiTPS15   DMASTER-EQ KGDHNVSTVQ CYMKEHG--- --TTMHESCK 479
SiTPS17   DIKSTER-EQ IRAHHASTVQ CYMLEHS--- --TTMCDACE 478
SiTPS11   DLQSYER-EQ LIPHVASTID SYMKEHN--- --ASIEMARE 534
SiTPS12   DLQSYER-EQ LIPHVASTID SYMKEHN--- --VSIEVARE 541
SiTPS3    DLQTYER-EQ LHPHVASTIK SYMKEHN--- --VSIEIARK 533
SiTPS4    DPKSYER-EQ MALHVASTID SCMKEHN--- --MSMELALK 543
SiTPS31   ------QGKC EKD-VASTVE CYMREYG--- --TTGEEAVA 408
SiTPS32   DIASYKQGKC EKD-VASTVE CYMREYG--- --TTGEEAVA 480
SiTPS25   DLSSYKLGKH KKD-MPSAVE TYMVENG--- --LKGEEAVA 502
SiTPS30   DITALKHGNR EQD-AANSVE CYIAEYN--- --VASEVAIA 431
SiTPS38   DMADFKRGRN KMD-VATSVE CYIKEHN--- --VSAEVALA 475
SiTPS1    DLGSAEDEAQ EGLDGSYRDF YLMENPS--- --CTQADAEE 482
SiTPS2    DLGSAEDEAQ EGLDGSYRDF YMMENLS--- --CTQADAEE 504
SiTPS5    DIQTYDK--E KSQGIINSVL LQVLGHGA-- --SSSEAAKR 642
SiTPS8    DIRTHKK--E MSEGCINSIR MCALRDGPEV SPASIEAAER 529
SiTPS13   DLRSYER--E AAQGKVNSVL L--LAADPPR SGGDLEASKR 689
SiTPS28   DAQTYER--E YKEGKVNIVS ILVHQSG--- GSMSIADARR 646
SiTPS29   DIQGFER--E GSEGKLNSVS LLVH-SG--- SSVSIEAAKK 719
PvKSL13   DSKSFER--E GSQGKLNSIS LLVLHSG--- NSVSTEAAKK 725
PvKSL15   DSKGFER--E GSQGKLNSIS LLVLHSG--- NSMSTEAAKK 725
ZmKSL3    DYQSLER--E GNQGKLNSVS LLVLHSG--- GSMSIEAAKK 721
OsKS1     DSQGFER--E SLEGKLNSVS LLVHHSG--- GSISIDEAKM 672
ZmKSL5    DVQSFER--E YKEGKLNIVS LLVLHGG--- -SMSISDARR 511
PpCPSKS   DIQGMKR--E ASQGKISSVQ IYMEEHPSVP ---SEAMAIA 807
```

Sequence logo 4.3bits 0.0bits

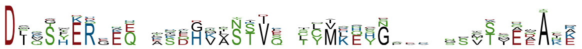

			900	920	
SiTPS19	KLRIIIEKAW	MDIVEECLD-	QK--QSMELL	EKAVNLGRTM	517
SiTPS20	KLRIIIEKAW	MDIVEECLD-	QK--QSMELL	EKAVNLGRTM	517
SiTPS21	KLRVIIEQAW	MDIIEECLE-	RK--RPMTLL	EIPVDLARTM	519
SiTPS22	KLRVIIEEAW	MDIVQEYLD-	QK--RPMELD	Q---------	468
SiTPS16	KLKVRLERAW	MDMVQGCLD-	QK--YPMVLL	EKVVSFAQSI	523
SiTPS18	KLKEILERAW	MDMVQECLD-	QK--YPMALL	EKVVSFAQSI	528
SiTPS26	KIREIVEEAW	MDMVQECLE-	QK--HPTAVL	EKVVNIARTM	522
SiTPS27	KLGETVEEAW	MDIVQECLD-	QK--HPMAVL	EKAVNLARTM	523
SiTPS36	KLGMLIDEAW	MDIVKGCLD-	GK--QPMH--	----------	571
SiTPS14	KIKQLSEDSW	KDMIQGSLVL	ED--QPKVVP	RTVLDLSRTA	517
SiTPS15	RIKELTEDLW	KDMLQHHLAR	TE--QTMIVS	HMVLNLARTG	517
SiTPS17	KIKELIEDKW	KDMMKLSLRP	TE--QPKLIA	KKVVDFARTA	516
SiTPS11	KILILKEESW	KDFNSEWLNP	NN-VYPKQLL	ERIFNLTRTM	573
SiTPS12	KIHTLKEESW	KDFNNEWLNS	DN-TIPKQLL	ERIFNLTRTM	580
SiTPS3	KIEELKEETW	KYFNGEWLNP	DN-AVPSSLL	ERIFNLTRTM	572
SiTPS4	KIKELTEESW	KSLNEEWLKP	NK-AQPKELL	ERIFNLTRSM	582
SiTPS31	AIAGMVEHAW	RRINKACMEV	KP-AV-EPVV	QCLLNTTRVL	446
SiTPS32	AITGMVEHAW	RRINKACIEV	KP-AV-EPVV	RCLLNTTRVL	518
SiTPS25	AIATLLENRW	RILNQASMKI	DR-TL-LPAV	QVVVNMARTN	540
SiTPS30	NIYRMVEDAW	KTANKDLLEL	G--SL-RPAV	RRVIDATASL	468
SiTPS38	KFGSFVDDAW	KTLNHAIFEH	H--AL-LPVL	QRVTNLAMSM	512
SiTPS1	HMRGLIAREW	EELNRECFS-	RR-TFASRFT	QVSLNAARMV	520
SiTPS2	HMRGLIAREW	EELNREGFS-	RR-TFASRFT	QVSLNAARMV	542
SiTPS5	EILGLVADSR	RELLRLVLSK	EA-TIPRSCR	DIFWNTYKLG	681
SiTPS8	EIRGVIADSR	RELLRLVVSE	AG-VLPRPCR	DIFWNSHKIA	568
SiTPS13	EMRSAIEASR	RELLRLVVGE	DGGAVPRPCR	RLFWNMSKVV	729
SiTPS28	EIQKPIDTCR	RDLLRLVLSK	EG-AIPRPCK	ELFWKMCKVC	685
SiTPS29	VIQKSIDTSR	RDLLRLVLRK	ES-IVPRPCK	ELFWKMCKIL	758
PvKSL13	VIQKSIDTSR	RDLLRLVLRK	ES-VVPRPCK	ELFWKMCKIL	764
PvKSL15	VIQKSIDKSR	RDLLRLVLRK	ES-VVPRPCK	ELFWKMCKIL	764
ZmKSL3	AMQKSIDVSR	RDLLRLVLRK	ES-AVPRPCK	ELFWKMCKIL	760
OsKS1	KAQKSIDTSR	RNLLRLVLG-	EQGAVPRPCK	QLFWKMCKIV	711
ZmKSL5	KLQKPIDTCR	RDLLRLVLRE	ES-VVPRPCK	ELFWKMCKVC	550
PpCPSKS	HLQELVDNSM	QQLTYEVLRF	T--AVPKSCK	RIHLNMAKIM	845

Sequence logo 4.3bits 0.0bits

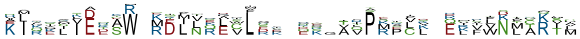

			940	960	
SiTPS19	DFMYKRE-DA	YTLSFSLKDI	IASMYVNFV-	-X--------	546
SiTPS20	DFMYKRE-DA	YTLSFSLKDI	IASMYVNFV-	-X--------	546
SiTPS21	DFVYKRD-DA	FTLSFSLKDV	IASMYVNSV-	-X--------	548
SiTPS22	----------	----------	----------	-L--------	469
SiTPS16	DFLYKSE-DL	YTLPCNLKET	LTSMYAKFV-	-X--------	552
SiTPS18	DFFYKSE-DL	YTLPCNLKET	LTSMYAKFV-	-X--------	557
SiTPS26	DFVYKRE-DA	YTLPHILKDV	MTSLYVNFV-	-X--------	551
SiTPS27	DFIYKRE-DA	YTLSYSLKDI	MTSLYVNFV-	-X--------	552
SiTPS36	----------	----------	--GIVGKGG-	-X--------	579
SiTPS14	VYMY-KQCDA	FTTSEILQEM	IKLLFVEPI-	-PEX------	548
SiTPS15	NYMYHNNVDK	FTSSHTIKDA	IKRLFVEPI-	-PMX------	549
SiTPS17	DYIY-KETDA	FTFSHTIKDM	IAMLYVEPA-	-X--------	545
SiTPS11	EFMYNQE-DN	FTNCTNLKDT	IHSLFAEPYT	KLIX------	606
SiTPS12	EFMYNQE-DN	FTNSTNLKDT	IHSLFVEPYT	KLIX------	613
SiTPS3	EFIYNLD-DN	FTNCQNLRDT	IHLLFVEPFP	-ISX------	604
SiTPS4	EFFYKQE-DA	YTNSCNIKDT	VKSLFVDSY-	KVFX------	614
SiTPS31	EAYYLHGRDG	LTYGRDLKEL	ITFLFLKDVM	SKFVICICRT	486
SiTPS32	EAYYLHGRDG	LTYGRDLKEL	ITFLFLKDV-	----------	547
SiTPS25	EIIYLHGRDG	YTFGDDLKDL	IITLFLKQV-	----------	569
SiTPS30	TLMYGDKKDT	FTFGDDLESL	ITRLFFSPI-	----------	497
SiTPS38	MLVFLDQRDG	YTNSKELKET	LKSQFVKHV-	----------	541
SiTPS1	GVMYSYDKEQ	RLL--VLEDY	ARMLLLX---	----------	545
SiTPS2	GVMYSYDKDG	RLL--VLEDY	ARMLLLX---	----------	567
SiTPS5	HLFYS-QGDG	FSMSQELAVA	VNAVVHEPL-	---QVTP---	713
SiTPS8	HHFYA-ERDG	FSRPKNLIAA	VNAVVHEPL-	---RGTP---	600
SiTPS13	HLFYM-EGDG	YASPKEMMRA	ANAVVLDPL-	---RRLPGKM	764
SiTPS28	YFFYS-QGDA	FSSPEAKARE	VDAVVNLPL-	---QL----K	716
SiTPS29	HLFYF-QTDG	FSSPKEMVSA	VNAVINEPL-	---KI----Q	789
PvKSL13	HLFYY-QTDG	FSSPKEMVGA	VNAVINEPL-	---------K	793
PvKSL15	HLFYF-QTDG	FSSPKEMVGA	VNAVINEPL-	---------K	793
ZmKSL3	HLFYS-QNDG	FSSPKEMVSA	VNAVINEPLK	----------	789
OsKS1	HMFYS-RTDG	FS--KEMVSA	VNAVVKEPL-	---------K	738
ZmKSL5	YFFYS-RTDG	FRSPVERAKE	VNAVINEPLK	LQENHTHMSV	589
PpCPSKS	HAFYK-DTDG	FSSLTAMTGF	VKKVLFEPVP	----------	874

Sequence logo 4.3bits 0.0bits

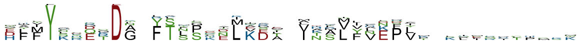

SiTPS19	- - - - - - - - - -	- - - -	546
SiTPS20	- - - - - - - - - -	- - - -	546
SiTPS21	- - - - - - - - - -	- - - -	548
SiTPS22	- - - - - - - - - -	- - - -	469
SiTPS16	- - - - - - - - - -	- - - -	552
SiTPS18	- - - - - - - - - -	- - - -	557
SiTPS26	- - - - - - - - - -	- - - -	551
SiTPS27	- - - - - - - - - -	- - - -	552
SiTPS36	- - - - - - - - - -	- - - -	579
SiTPS14	- - - - - - - - - -	- - - -	548
SiTPS15	- - - - - - - - - -	- - - -	549
SiTPS17	- - - - - - - - - -	- - - -	545
SiTPS11	- - - - - - - - - -	- - - -	606
SiTPS12	- - - - - - - - - -	- - - -	613
SiTPS3	- - - - - - - - - -	- - - -	604
SiTPS4	- - - - - - - - - -	- - - -	614
SiTPS31	Y T V L H I Y A C V	E K K X	500
SiTPS32	- - - - H V X - - -	- - - -	550
SiTPS25	- - - - - - - - - -	- P L X	572
SiTPS30	- - - - - - - - - -	- T I X	500
SiTPS38	- - - - - - - - - -	- P L X	544
SiTPS1	- - - - - - - - - -	- - - -	545
SiTPS2	- - - - - - - - - -	- - - -	567
SiTPS5	- - - - - - P S R L	K R G X	721
SiTPS8	- - - - - - - - - -	- - S X	602
SiTPS13	R I D S R G T S S F	L N F X	778
SiTPS28	G S N A N K L P V L	W E X -	729
SiTPS29	L D D S S - L N I L	S E K X	802
PvKSL13	I Q M S D A S L F I	S S E K	807
PvKSL15	I Q M S D A S L F I	S S E K	807
ZmKSL3	- V Q N S T T F L S	S S S R	802
OsKS1	L K V S D P Y G S I	L S G N	752
ZmKSL5	L - - - - - - - - -	- - - -	590
PpCPSKS	- - - - - - - - - -	- - - E	875

4.3bits

Sequence logo

0.0bits
