# Supplementary material for: The foxtail millet (Setaria italica) terpene synthase gene family
Source: Plant J. 2020 May 3;103(2):781–800. doi: 10.1111/tpj.14771 (PMC7497057; doi:10.1111/tpj.14771)
Supplement: Supplementary file 1 — Figure S1. Protein sequence alignment of select class II diterpene synthases. Figure S2. Protein sequence alignment of select class I diterpene synthases. Figure S3. Sequence similarity matrix of terpene synthase candidates from Setaria italica and Setaria viridis. Figure S4. Mass spectra of class II diterpene synthase products identified in this study. Figure S5. Mass spectra of class I terpene synthase products identified in this study. Figure S6. Mass spectra of products resulting from co‐expression assays of SiTPS5 and SiTPS13. Figure S7. The NMR analysis of ent‐pimara‐8,15‐diene. Figure S8. The NMR analysis of syn‐pimara‐7,15‐diene. Figure S9. The NMR analysis of eudesme‐2,11‐diol. Figure S10. Functional analysis of CYP99A17 and CYP99A19. Figure S11. The NMR analysis of abietadien‐19‐ol. Figure S12. The NMR analysis of syn‐pimara‐7,15‐dien‐19‐ol. Figure S13. Gene expression analysis of characterized Setaria italica terpene synthase genes. Figure S14. Occurrence of terpene synthase and CYP99A17 products in Setaria italica. [file TPJ-103-781-s001.zip › tpj14771-sup-0003-FigS3.pdf]

		1	2	3	4	5	6	7	8	9	10	11	12	13	14	15	16	17
SiTPS19	1		98.18	90.66	91.21	70.67	71.04	60.29	68.55	65.52	65.16	66.49	65.89	67.33	67.15	65.34	55.28	52.61
SvTPS17	2	98.18		90.15	90.69	70.67	71.04	60.07	68.36	65.70	65.34	66.85	66.25	67.51	67.33	65.52	55.28	52.61
SiTPS20	3	90.66	90.15		98.72	69.58	70.13	58.47	67.27	65.52	65.16	66.13	65.54	65.52	65.34	63.36	53.98	51.47
SvTPS18	4	91.21	90.69	98.72		69.95	70.49	59.02	67.82	65.52	65.16	66.13	65.54	65.88	65.52	63.72	54.15	51.63
SiTPS21	5	70.67	70.67	69.58	69.95		98.36	64.61	74.59	60.00	60.00	60.29	59.54	64.80	64.80	63.90	53.98	51.14
SvTPS19	6	71.04	71.04	70.13	70.49	98.36		64.43	74.23	60.72	60.72	61.00	60.25	64.98	64.98	63.72	54.15	51.30
SiTPS22	7	60.29	60.07	58.47	59.02	64.61	64.43		81.67	53.32	53.50	53.39	53.02	54.04	53.86	52.24	51.45	47.53
SvTPS20	8	68.55	68.36	67.27	67.82	74.59	74.23	81.67		60.61	60.79	60.54	60.14	62.23	62.05	60.61	52.84	49.84
SiTPS16	9	65.52	65.70	65.52	65.52	60.00	60.72	53.32	60.61		99.46	93.91	93.04	59.68	60.04	58.60	48.78	46.92
SvTPS14	10	65.16	65.34	65.16	65.16	60.00	60.72	53.50	60.79	99.46		93.37	92.50	59.32	59.68	58.96	48.95	46.75
SiTPS18	11	66.49	66.85	66.13	66.13	60.29	61.00	53.39	60.54	93.91	93.37		96.97	60.68	61.03	59.96	49.03	47.50
SvTPS16	12	65.89	66.25	65.54	65.54	59.54	60.25	53.02	60.14	93.04	92.50	96.97		60.04	60.39	59.50	49.03	47.33
SiTPS27	13	67.33	67.51	65.52	65.88	64.80	64.98	54.04	62.23	59.68	59.32	60.68	60.04		99.09	90.76	53.75	51.55
SvTPS22	14	67.15	67.33	65.34	65.52	64.80	64.98	53.86	62.05	60.04	59.68	61.03	60.39	99.09		92.03	53.91	51.71
SiTPS26	15	65.34	65.52	63.36	63.72	63.90	63.72	52.24	60.61	58.60	58.96	59.96	59.50	90.76	92.03		52.44	50.24
SiTPS36	16	55.28	55.28	53.98	54.15	53.98	54.15	51.45	52.84	48.78	48.95	49.03	49.03	53.75	53.91	52.44		91.24
SvTPS28	17	52.61	52.61	51.47	51.63	51.14	51.30	47.53	49.84	46.92	46.75	47.50	47.33	51.55	51.71	50.24	91.24	
SiTPS14	18	38.39	38.75	38.57	38.39	37.86	37.68	31.25	35.77	35.71	35.54	35.16	34.86	38.43	38.79	38.26	32.20	31.22
SvTPS12	19	38.39	38.75	38.75	38.39	37.86	37.68	31.25	35.77	35.71	35.54	35.16	34.86	38.43	38.79	38.26	32.20	31.22
SvTPS30	20	36.27	36.27	35.91	35.91	35.01	35.01	28.60	34.17	33.09	32.92	33.45	33.16	36.43	36.79	37.57	29.43	28.46
SiTPS15	21	40.00	40.18	39.82	39.82	41.25	41.25	34.94	38.79	37.50	37.32	37.63	37.32	41.81	42.17	41.10	35.88	34.42
SvTPS13	22	39.82	40.00	39.64	39.64	41.25	41.25	34.76	38.61	37.32	37.14	37.46	37.15	42.17	42.53	41.46	35.71	34.25
SiTPS17	23	41.83	41.83	40.93	40.75	40.04	40.39	33.75	38.82	39.43	39.43	39.54	39.22	41.61	42.32	42.68	36.05	34.58
SvTPS15	24	41.65	41.65	40.75	40.57	39.86	40.22	33.57	38.64	39.25	39.25	39.36	39.05	41.43	42.14	42.50	36.05	34.58
SiTPS11	25	31.13	31.08	31.77	31.45	31.03	31.35	26.21	30.34	30.23	30.23	30.57	31.15	31.84	32.00	31.84	28.29	26.93
SvTPS32	26	31.45	31.40	32.10	31.77	31.35	31.67	26.37	30.66	30.23	30.23	30.57	31.15	31.84	32.00	31.84	28.14	26.78
SvTPS9	27	27.58	27.54	28.39	28.06	27.17	27.49	22.35	26.65	26.21	26.21	26.59	27.16	28.16	28.32	28.53	23.90	22.54
SiTPS12	28	33.23	33.23	33.23	32.91	32.38	33.01	27.55	32.48	30.78	30.78	30.96	31.38	33.65	33.81	33.81	29.99	28.79
SvTPS10	29	33.07	33.07	33.07	32.75	32.22	32.70	27.42	32.33	30.63	30.63	30.82	31.23	33.49	33.65	33.65	29.85	28.66
SiTPS3	30	32.52	32.36	32.36	32.20	32.31	32.15	27.42	31.94	29.38	29.38	29.90	30.32	33.01	33.17	32.85	30.23	28.85
SvTPS3	31	32.69	32.52	32.52	32.36	32.47	32.31	27.58	32.10	29.55	29.55	30.06	30.48	33.17	33.33	33.01	30.38	29.01
SiTPS4	32	35.26	35.10	34.62	34.62	33.92	34.56	29.19	34.03	31.83	31.83	32.64	32.75	35.89	35.73	35.41	31.98	30.62
SvTPS4	33	35.26	35.10	34.62	34.62	33.92	34.56	29.19	34.03	31.83	31.83	32.64	32.75	35.89	35.73	35.41	31.98	30.62
SiTPS30	34	27.45	27.53	27.27	27.45	25.75	25.93	25.29	24.78	27.21	27.03	27.67	27.23	26.86	27.39	27.56	22.63	21.70
SvTPS25	35	30.30	30.20	29.95	30.12	28.42	28.60	27.97	27.43	29.86	29.68	30.12	29.67	29.15	29.51	29.68	25.20	24.12
SvTPS33	36	28.52	28.42	28.35	28.52	26.49	26.67	25.46	25.70	27.92	27.75	28.32	27.88	27.40	27.75	27.92	23.49	22.73
SiTPS38	37	30.88	30.94	32.11	32.28	29.37	29.02	25.85	28.75	30.78	30.61	30.81	30.53	30.09	30.43	30.09	25.47	24.56
SvTPS29	38	30.88	30.94	32.11	32.28	29.20	29.02	25.85	28.92	30.78	30.61	30.98	30.70	30.09	30.43	29.91	25.47	24.56
SiTPS32	39	31.59	31.65	30.89	31.06	29.62	29.62	26.06	29.64	32.17	32.00	31.03	30.70	33.16	33.51	32.99	25.83	23.97
SvTPS34	40	31.59	31.65	31.06	31.24	29.79	29.79	26.60	29.98	32.35	32.17	31.21	30.87	33.33	33.68	33.16	25.83	23.97
SiTPS31	41	27.44	27.52	26.93	27.10	26.20	26.20	25.05	25.38	27.67	27.50	26.77	26.51	29.47	29.98	29.47	22.17	20.34
SiTPS25	42	30.05	30.39	29.88	30.05	29.42	29.59	26.28	30.46	30.61	30.44	30.69	30.87	31.86	32.03	30.85	25.58	24.53
SvTPS21	43	30.10	30.27	29.76	29.93	28.28	28.45	25.43	29.83	29.81	29.64	29.90	30.08	30.56	30.90	29.71	27.17	26.28
SiTPS1	44	22.26	22.22	22.26	22.09	20.31	20.48	17.02	18.97	19.15	18.97	19.32	19.49	22.26	22.43	21.40	18.85	17.89
SvTPS1	45	22.60	22.56	22.60	22.43	20.65	20.82	17.37	19.32	19.49	19.32	19.66	19.83	22.60	22.77	21.75	19.17	18.05
SiTPS2	46	20.67	20.64	20.50	20.34	19.26	19.43	16.27	17.79	17.62	17.79	17.79	17.95	21.34	21.51	21.01	17.11	16.33
SvTPS2	47	20.84	20.81	20.67	20.50	19.43	19.60	16.27	17.79	17.79	17.95	17.95	18.12	21.51	21.68	21.18	17.27	16.48
SvTPS31	48	15.20	15.17	15.03	14.85	13.43	13.60	12.77	13.92	12.98	12.80	13.10	12.93	15.89	16.23	15.72	13.57	12.93
SiTPS8	49	13.20	13.16	13.20	13.05	13.74	14.04	14.24	14.16	14.58	14.58	14.49	14.51	14.58	14.72	14.72	13.93	13.42
SvTPS7	50	13.20	13.16	13.20	13.05	13.74	14.04	14.24	14.16	14.58	14.58	14.49	14.51	14.58	14.72	14.72	13.93	13.42
SiTPS5	51	12.89	13.03	12.63	12.50	13.80	14.06	12.72	13.40	13.35	13.35	13.22	13.22	13.65	13.78	13.39	13.65	13.04
SvTPS5	52	12.63	12.76	12.37	12.24	13.80	14.06	12.58	13.40	13.61	13.61	13.48	13.48	13.65	13.78	13.65	13.78	13.17
SiTPS13	53	11.45	11.21	11.45	11.21	11.56	11.92	11.02	11.92	11.65	11.65	11.65	11.65	11.66	11.79	11.54	12.20	11.88
SvTPS11	54	11.77	11.65	11.65	11.40	12.13	12.50	11.71	12.50	12.10	12.10	12.10	12.10	12.36	12.48	12.24	12.77	12.32
SiTPS29	55	11.72	11.72	12.08	11.96	12.43	12.43	11.23	12.07	12.17	12.17	12.29	12.41	12.43	12.66	12.90	12.35	11.92
SvTPS24	56	11.72	11.72	12.08	11.96	12.43	12.43	11.23	12.07	12.17	12.17	12.29	12.41	12.43	12.66	12.90	12.35	11.92
SiTPS28	57	13.86	13.86	13.73	13.59	13.71	13.84	12.89	13.97	13.33	13.33	13.59	13.73	13.71	13.97	14.10	14.36	13.89
SvTPS23	58	13.86	13.86	13.73	13.59	13.71	13.84	12.89	13.97	13.33	13.33	13.59	13.73	13.58	13.84	13.97	14.36	13.89
SiTPS34	59	9.37	9.71	9.37	9.26	9.04	9.04	8.98	9.47	8.56	8.56	8.56	8.68	8.80	8.91	8.69	9.77	9.23
SvTPS26	60	9.37	9.71	9.37	9.26	9.04	9.04	8.98	9.47	8.56	8.56	8.56	8.68	8.80	8.91	8.69	9.77	9.23
SiTPS35	61	9.85	10.08	10.08	9.97	9.52	9.52	9.09	9.84	9.38	9.38	9.50	9.73	9.51	9.62	9.62	10.46	9.92
SvTPS27	62	9.67	9.90	9.90	9.79	9.35	9.35	8.93	9.66	9.21	9.21	9.33	9.55	9.34	9.45	9.45	10.27	9.75
SiTPS6	63	9.95	10.07	9.59	9.59	8.76	8.88	8.67	9.70	9.82	9.82	9.82	9.70	9.35	9.47	8.99	11.46	10.95
SvTPS6	64	10.02	10.15	9.65	9.65	8.80	8.92	8.71	9.77	9.89	9.89	9.89	9.77	9.41	9.53	9.03	10.86	10.46
SiTPS9	65	10.02	10.14	10.26	10.14	9.56	9.68	8.64	9.54	9.66	9.66	9.89	9.89	9.91	10.02	9.32	10.06	9.21
SvTPS8	66	10.02	10.14	10.26	10.14	9.56	9.68	8.64	9.54	9.66	9.66	9.89	9.89	9.91	10.02	9.32	10.06	9.21

		18	19	20	21	22	23	24	25	26	27	28	29	30	31	32	33
SiTPS19	1	38.39	38.39	36.27	40.00	39.82	41.83	41.65	31.13	31.45	27.58	33.23	33.07	32.52	32.69	35.26	35.26
SvTPS17	2	38.75	38.75	36.27	40.18	40.00	41.83	41.65	31.08	31.40	27.54	33.23	33.07	32.36	32.52	35.10	35.10
SiTPS20	3	38.57	38.75	35.91	39.82	39.64	40.93	40.75	31.77	32.10	28.39	33.23	33.07	32.36	32.52	34.62	34.62
SvTPS18	4	38.39	38.39	35.91	39.82	39.64	40.75	40.57	31.45	31.77	28.06	32.91	32.75	32.20	32.36	34.62	34.62
SiTPS21	5	37.86	37.86	35.01	41.25	41.25	40.04	39.86	31.03	31.35	27.17	32.38	32.22	32.31	32.47	33.92	33.92
SvTPS19	6	37.68	37.68	35.01	41.25	41.25	40.39	40.22	31.35	31.67	27.49	33.01	32.70	32.15	32.31	34.56	34.56
SiTPS22	7	31.25	31.25	28.60	34.94	34.76	33.75	33.57	26.21	26.37	22.35	27.55	27.42	27.42	27.58	29.19	29.19
SvTPS20	8	35.77	35.77	34.17	38.79	38.61	38.82	38.64	30.34	30.66	26.65	32.48	32.33	31.94	32.10	34.03	34.03
SiTPS16	9	35.71	35.71	33.09	37.50	37.32	39.43	39.25	30.23	30.23	26.21	30.78	30.63	29.38	29.55	31.83	31.83
SvTPS14	10	35.54	35.54	32.92	37.32	37.14	39.43	39.25	30.23	30.23	26.21	30.78	30.63	29.38	29.55	31.83	31.83
SiTPS18	11	35.16	35.16	33.45	37.63	37.46	39.54	39.36	30.57	30.57	26.59	30.96	30.82	29.90	30.06	32.64	32.64
SvTPS16	12	34.86	34.86	33.16	37.32	37.15	39.22	39.05	31.15	31.15	27.16	31.38	31.23	30.32	30.48	32.75	32.75
SiTPS27	13	38.43	38.43	36.43	41.81	42.17	41.61	41.43	31.84	31.84	28.16	33.65	33.49	33.01	33.17	35.89	35.89
SvTPS22	14	38.79	38.79	36.79	42.17	42.53	42.32	42.14	32.00	32.00	28.32	33.81	33.65	33.17	33.33	35.73	35.73
SiTPS26	15	38.26	38.26	37.57	41.10	41.46	42.68	42.50	31.84	31.84	28.53	33.81	33.65	32.85	33.01	35.41	35.41
SiTPS36	16	32.20	32.20	29.43	35.88	35.71	36.05	36.05	28.29	28.14	23.90	29.99	29.85	30.23	30.38	31.98	31.98
SvTPS28	17	31.22	31.22	28.46	34.42	34.25	34.58	34.58	26.93	26.78	22.54	28.79	28.66	28.85	29.01	30.62	30.62
SiTPS14	18		99.45	63.02	58.91	58.73	57.45	57.27	34.19	34.03	29.70	36.15	35.97	35.49	35.66	36.60	36.60
SvTPS12	19	99.45		62.84	58.91	58.73	57.45	57.27	34.19	34.03	29.70	36.31	36.13	35.49	35.66	36.60	36.60
SvTPS30	20	63.02	62.84		53.55	53.55	53.56	53.38	30.42	30.26	31.72	32.74	32.59	32.36	32.52	32.53	32.53
SiTPS15	21	58.91	58.91	53.55		99.45	59.82	59.64	33.44	33.44	29.10	36.52	36.19	35.71	35.88	38.42	38.42
SvTPS13	22	58.73	58.73	53.55	99.45		60.18	60.00	33.76	33.76	29.42	36.84	36.51	36.04	36.20	38.59	38.59
SiTPS17	23	57.45	57.45	53.56	59.82	60.18		99.82	35.00	34.68	30.00	38.08	37.90	37.18	37.34	36.66	36.66
SvTPS15	24	57.27	57.27	53.38	59.64	60.00	99.82		35.00	34.68	30.00	38.08	37.90	37.18	37.34	36.66	36.66
SiTPS11	25	34.19	34.19	30.42	33.44	33.76	35.00	35.00		98.84	89.77	73.02	73.34	64.89	65.05	53.67	53.67
SvTPS32	26	34.03	34.03	30.26	33.44	33.76	34.68	34.68	98.84		90.43	73.02	73.34	64.56	64.72	53.83	53.83
SvTPS9	27	29.70	29.70	31.72	29.10	29.42	30.00	30.00	89.77	90.43		66.72	67.04	58.90	59.06	49.36	49.36
SiTPS12	28	36.15	36.31	32.74	36.52	36.84	38.08	38.08	73.02	73.02	66.72		99.03	65.92	66.08	56.32	56.32
SvTPS10	29	35.97	36.13	32.59	36.19	36.51	37.90	37.90	73.34	73.34	67.04	99.03		66.08	66.24	56.32	56.32
SiTPS3	30	35.49	35.49	32.36	35.71	36.04	37.18	37.18	64.89	64.56	58.90	65.92	66.08		99.83	59.55	59.55
SvTPS3	31	35.66	35.66	32.52	35.88	36.20	37.34	37.34	65.05	64.72	59.06	66.08	66.24	99.83		59.55	59.55
SiTPS4	32	36.60	36.60	32.53	38.42	38.59	36.66	36.66	53.67	53.83	49.36	56.32	56.32	59.55	59.55		100.00
SvTPS4	33	36.60	36.60	32.53	38.42	38.59	36.66	36.66	53.67	53.83	49.36	56.32	56.32	59.55	59.55	100.00	
SiTPS30	34	31.85	31.85	27.06	32.68	33.04	31.79	31.61	25.20	25.20	21.86	26.83	26.70	26.85	26.85	25.28	25.28
SvTPS25	35	34.52	34.52	29.39	34.99	35.35	34.29	34.11	27.56	27.56	23.63	29.21	29.07	29.42	29.42	27.98	27.98
SvTPS33	36	32.63	32.63	27.74	33.27	33.63	32.45	32.28	26.16	25.84	21.88	28.21	28.08	27.84	27.84	26.27	26.27
SiTPS38	37	33.22	33.22	28.47	32.81	33.16	30.93	30.93	29.94	30.10	26.32	32.91	32.76	31.10	31.10	32.27	32.27
SvTPS29	38	33.22	33.22	28.47	32.81	33.16	30.93	30.93	29.94	30.10	26.32	32.91	32.76	31.10	31.10	32.43	32.43
SiTPS32	39	32.64	32.64	29.25	33.62	33.97	33.10	33.10	28.48	28.48	24.29	30.09	29.95	30.25	30.25	28.16	28.16
SvTPS34	40	32.64	32.64	29.07	33.97	34.32	33.45	33.45	28.80	28.80	24.60	30.25	30.11	30.25	30.25	28.48	28.48
SiTPS31	41	29.16	29.16	25.34	29.62	29.97	28.13	28.13	24.61	24.61	20.59	25.77	25.65	26.37	26.37	24.19	24.19
SiTPS25	42	32.37	32.37	27.30	32.99	33.16	32.42	32.42	28.86	28.86	25.04	32.24	32.09	29.68	29.68	29.11	29.11
SvTPS21	43	32.88	32.88	28.67	33.67	34.18	33.79	33.62	29.17	29.17	25.35	32.81	32.66	29.64	29.64	29.86	29.86
SiTPS1	44	21.71	21.54	18.52	20.34	20.51	20.93	20.93	20.94	20.94	18.74	21.68	21.58	20.00	20.00	20.28	20.28
SvTPS1	45	22.05	21.88	18.87	20.68	20.85	21.27	21.27	21.26	21.26	18.90	22.00	21.89	20.32	20.32	20.60	20.60
SiTPS2	46	21.14	20.97	17.51	20.30	20.47	21.21	21.21	20.40	20.40	18.39	21.32	21.22	20.44	20.44	19.78	19.78
SvTPS2	47	21.14	20.97	17.68	20.30	20.47	21.21	21.21	20.40	20.40	18.39	21.32	21.22	20.44	20.44	19.78	19.78
SvTPS31	48	16.09	15.92	13.72	15.92	15.92	14.93	14.93	14.90	14.90	12.84	15.57	15.49	16.03	16.03	14.72	14.72
SiTPS8	49	14.41	14.41	14.26	14.41	14.26	14.75	14.75	14.71	14.43	12.78	15.27	15.41	15.13	15.13	15.78	15.78
SvTPS7	50	14.41	14.41	14.26	14.56	14.41	14.75	14.75	14.71	14.43	12.78	15.27	15.41	15.13	15.13	15.78	15.78
SiTPS5	51	13.95	13.95	13.94	15.39	15.26	14.13	14.13	14.40	14.27	12.88	14.94	15.07	15.67	15.67	16.43	16.43
SvTPS5	52	13.95	13.95	13.94	15.39	15.26	14.13	14.13	14.40	14.27	12.88	14.81	14.94	15.67	15.67	16.30	16.30
SiTPS13	53	11.83	11.83	10.79	11.59	11.59	12.24	12.24	11.62	11.38	10.21	11.52	11.64	11.86	11.86	12.20	12.20
SvTPS11	54	12.16	12.16	11.24	12.03	12.03	12.70	12.70	12.19	11.95	10.75	11.96	12.08	12.44	12.44	12.65	12.65
SiTPS29	55	12.47	12.47	11.31	12.23	12.11	12.24	12.24	12.86	12.98	11.47	13.10	13.33	12.74	12.86	12.86	12.86
SvTPS24	56	12.47	12.47	11.19	12.11	11.99	12.12	12.12	12.86	12.98	11.47	13.10	13.33	12.62	12.74	12.74	12.74
SiTPS28	57	14.17	14.17	13.58	14.83	14.70	14.45	14.45	13.54	13.29	11.82	13.85	13.80	14.36	14.49	14.58	14.58
SvTPS23	58	13.91	13.91	13.45	14.83	14.70	14.32	14.32	13.54	13.29	11.82	13.85	13.80	14.49	14.61	14.70	14.70
SiTPS34	59	10.26	10.26	8.37	9.91	9.91	9.59	9.59	10.09	9.98	8.54	10.20	10.43	10.44	10.44	10.22	10.22
SvTPS26	60	10.15	10.15	8.37	9.91	9.91	9.59	9.59	10.09	9.98	8.54	10.20	10.43	10.44	10.44	10.22	10.22
SiTPS35	61	10.06	10.06	8.74	10.73	10.73	9.73	9.73	10.23	10.11	8.68	10.11	10.34	10.47	10.47	10.58	10.58
SvTPS27	62	9.88	9.88	8.58	10.54	10.54	9.55	9.55	10.04	9.93	8.52	9.93	10.16	10.28	10.28	10.39	10.39
SiTPS6	63	11.02	11.02	8.61	11.12	11.12	10.67	10.67	10.11	10.11	8.44	10.11	10.46	9.88	9.88	9.88	9.88
SvTPS6	64	11.23	11.23	8.64	11.22	11.22	10.88	10.88	10.31	10.31	8.59	10.31	10.67	10.07	10.07	10.07	10.07
SiTPS9	65	11.08	11.08	8.70	10.84	11.07	10.15	10.15	9.36	9.47	7.87	10.06	10.41	10.42	10.42	10.30	10.30
SvTPS8	66	11.08	11.08	8.70	10.84	11.07	10.15	10.15	9.36	9.47	7.87	10.06	10.41	10.42	10.42	10.30	10.30

		34	35	36	37	38	39	40	41	42	43	44	45	46	47	48	49	50
SiTPS19	1	27.45	30.30	28.52	30.88	30.88	31.59	31.59	27.44	30.05	30.10	22.26	22.60	20.67	20.84	15.20	13.20	13.20
SvTPS17	2	27.53	30.20	28.42	30.94	30.94	31.65	31.65	27.52	30.39	30.27	22.22	22.56	20.64	20.81	15.17	13.16	13.16
SiTPS20	3	27.27	29.95	28.35	32.11	32.11	30.89	31.06	26.93	29.88	29.76	22.26	22.60	20.50	20.67	15.03	13.20	13.20
SvTPS18	4	27.45	30.12	28.52	32.28	32.28	31.06	31.24	27.10	30.05	29.93	22.09	22.43	20.34	20.50	14.85	13.05	13.05
SiTPS21	5	25.75	28.42	26.49	29.37	29.20	29.62	29.79	26.20	29.42	28.28	20.31	20.65	19.26	19.43	13.43	13.74	13.74
SvTPS19	6	25.93	28.60	26.67	29.02	29.02	29.62	29.79	26.20	29.59	28.45	20.48	20.82	19.43	19.60	13.60	14.04	14.04
SiTPS22	7	25.29	27.97	25.46	25.85	25.85	26.06	26.60	25.05	26.28	25.43	17.02	17.37	16.27	16.27	12.77	14.24	14.24
SvTPS20	8	24.78	27.43	25.70	28.75	28.92	29.64	29.98	25.38	30.46	29.83	18.97	19.32	17.79	17.79	13.92	14.16	14.16
SiTPS16	9	27.21	29.86	27.92	30.78	30.78	32.17	32.35	27.67	30.61	29.81	19.15	19.49	17.62	17.79	12.98	14.58	14.58
SvTPS14	10	27.03	29.68	27.75	30.61	30.61	32.00	32.17	27.50	30.44	29.64	18.97	19.32	17.79	17.95	12.80	14.58	14.58
SiTPS18	11	27.67	30.12	28.32	30.81	30.98	31.03	31.21	26.77	30.69	29.90	19.32	19.66	17.79	17.95	13.10	14.49	14.49
SvTPS16	12	27.23	29.67	27.88	30.53	30.70	30.70	30.87	26.51	30.87	30.08	19.49	19.83	17.95	18.12	12.93	14.51	14.51
SiTPS27	13	26.86	29.15	27.40	30.09	30.09	33.16	33.33	29.47	31.86	30.56	22.26	22.60	21.34	21.51	15.89	14.58	14.58
SvTPS22	14	27.39	29.51	27.75	30.43	30.43	33.51	33.68	29.98	32.03	30.90	22.43	22.77	21.51	21.68	16.23	14.72	14.72
SiTPS26	15	27.56	29.68	27.92	30.09	29.91	32.99	33.16	29.47	30.85	29.71	21.40	21.75	21.01	21.18	15.72	14.72	14.72
SiTPS36	16	22.63	25.20	23.49	25.47	25.47	25.83	25.83	22.17	25.58	27.17	18.85	19.17	17.11	17.27	13.57	13.93	13.93
SvTPS28	17	21.70	24.12	22.73	24.56	24.56	23.97	23.97	20.34	24.53	26.28	17.89	18.05	16.33	16.48	12.93	13.42	13.42
SiTPS14	18	31.85	34.52	32.63	33.22	33.22	32.64	32.64	29.16	32.37	32.88	21.71	22.05	21.14	21.14	16.09	14.41	14.41
SvTPS12	19	31.85	34.52	32.63	33.22	33.22	32.64	32.64	29.16	32.37	32.88	21.54	21.88	20.97	20.97	15.92	14.41	14.41
SvTPS30	20	27.06	29.39	27.74	28.47	28.47	29.25	29.07	25.34	27.30	28.67	18.52	18.87	17.51	17.68	13.72	14.26	14.26
SiTPS15	21	32.68	34.99	33.27	32.81	32.81	33.62	33.97	29.62	32.99	33.67	20.34	20.68	20.30	20.30	15.92	14.41	14.56
SvTPS13	22	33.04	35.35	33.63	33.16	33.16	33.97	34.32	29.97	33.16	34.18	20.51	20.85	20.47	20.47	15.92	14.26	14.41
SiTPS17	23	31.79	34.29	32.45	30.93	30.93	33.10	33.45	28.13	32.42	33.79	20.93	21.27	21.21	21.21	14.93	14.75	14.75
SvTPS15	24	31.61	34.11	32.28	30.93	30.93	33.10	33.45	28.13	32.42	33.62	20.93	21.27	21.21	21.21	14.93	14.75	14.75
SiTPS11	25	25.20	27.56	26.16	29.94	29.94	28.48	28.80	24.61	28.86	29.17	20.94	21.26	20.40	20.40	14.90	14.71	14.71
SvTPS32	26	25.20	27.56	25.84	30.10	30.10	28.48	28.80	24.61	28.86	29.17	20.94	21.26	20.40	20.40	14.90	14.43	14.43
SvTPS9	27	21.86	23.63	21.88	26.32	26.32	24.29	24.60	20.59	25.04	25.35	18.74	18.90	18.39	18.39	12.84	12.78	12.78
SiTPS12	28	26.83	29.21	28.21	32.91	32.91	30.09	30.25	25.77	32.24	32.81	21.68	22.00	21.32	21.32	15.57	15.27	15.27
SvTPS10	29	26.70	29.07	28.08	32.76	32.76	29.95	30.11	25.65	32.09	32.66	21.58	21.89	21.22	21.22	15.49	15.41	15.41
SiTPS3	30	26.85	29.42	27.84	31.10	31.10	30.25	30.25	26.37	29.68	29.64	20.00	20.32	20.44	20.44	16.03	15.13	15.13
SvTPS3	31	26.85	29.42	27.84	31.10	31.10	30.25	30.25	26.37	29.68	29.64	20.00	20.32	20.44	20.44	16.03	15.13	15.13
SiTPS4	32	25.28	27.98	26.27	32.27	32.43	28.16	28.48	24.19	29.11	29.86	20.28	20.60	19.78	19.78	14.72	15.78	15.78
SvTPS4	33	25.28	27.98	26.27	32.27	32.43	28.16	28.48	24.19	29.11	29.86	20.28	20.60	19.78	19.78	14.72	15.78	15.78
SiTPS30	34		95.83	88.31	46.97	46.97	39.10	38.92	38.26	35.71	36.17	20.32	20.67	19.83	19.83	16.30	14.24	14.08
SvTPS25	35	95.83		91.76	49.82	49.82	41.70	41.52	40.49	38.57	39.20	22.46	22.81	21.88	21.88	17.27	16.95	16.80
SvTPS33	36	88.31	91.76		48.44	48.44	39.78	39.78	37.27	36.52	36.87	20.00	20.34	19.56	19.56	15.90	14.98	14.83
SiTPS38	37	46.97	49.82	48.44		99.45	43.81	44.17	37.83	44.25	44.95	20.96	21.31	19.56	19.56	15.16	15.92	15.77
SvTPS29	38	46.97	49.82	48.44	99.45		43.63	43.99	37.65	44.08	44.77	20.96	21.31	19.39	19.39	15.16	15.77	15.62
SiTPS32	39	39.10	41.70	39.78	43.81	43.63		98.91	78.88	55.56	54.69	24.05	24.40	22.43	22.60	16.84	14.52	14.52
SvTPS34	40	38.92	41.52	39.78	44.17	43.99	98.91		79.06	56.08	55.21	23.88	24.23	22.77	22.60	16.49	14.52	14.52
SiTPS31	41	38.26	40.49	37.27	37.83	37.65	78.88	79.06		47.56	47.15	21.94	22.28	20.98	21.15	16.76	13.56	13.56
SiTPS25	42	35.71	38.57	36.52	44.25	44.08	55.56	56.08	47.56		86.06	20.00	20.33	19.48	19.64	14.98	12.36	12.36
SvTPS21	43	36.17	39.20	36.87	44.95	44.77	54.69	55.21	47.15	86.06		20.17	20.50	19.64	19.80	15.15	13.34	13.34
SiTPS1	44	20.32	22.46	20.00	20.96	20.96	24.05	23.88	21.94	20.00	20.17		100.00	82.36	82.54	53.28	13.95	13.95
SvTPS1	45	20.67	22.81	20.34	21.31	21.31	24.40	24.23	22.28	20.33	20.50	100.00		82.54	82.54	53.28	14.24	14.24
SiTPS2	46	19.83	21.88	19.56	19.56	19.39	22.43	22.77	20.98	19.48	19.64	82.36	82.54		99.12	47.10	12.79	12.79
SvTPS2	47	19.83	21.88	19.56	19.56	19.39	22.60	22.60	21.15	19.64	19.80	82.54	82.54	99.12		46.92	12.79	12.79
SvTPS31	48	16.30	17.27	15.90	15.16	15.16	16.84	16.49	16.76	14.98	15.15	53.28	53.28	47.10	46.92		9.70	9.70
SiTPS8	49	14.24	16.95	14.98	15.92	15.77	14.52	14.52	13.56	12.36	13.34	13.95	14.24	12.79	12.79	9.70		99.83
SvTPS7	50	14.08	16.80	14.83	15.77	15.62	14.52	14.52	13.56	12.36	13.34	13.95	14.24	12.79	12.79	9.70	99.83	
SiTPS5	51	13.25	15.63	14.32	14.29	14.15	12.88	12.88	11.42	11.63	12.53	12.35	12.61	11.92	11.92	9.02	49.66	49.79
SvTPS5	52	13.11	15.50	14.19	14.02	13.89	12.75	12.75	11.29	11.50	12.40	12.35	12.61	11.92	11.92	8.89	50.07	50.21
SiTPS13	53	10.82	12.79	11.33	12.78	12.65	11.60	11.60	10.18	11.07	11.79	12.06	12.18	11.42	11.42	9.10	38.45	38.58
SvTPS11	54	11.10	13.09	11.61	12.95	12.83	11.63	11.63	10.31	11.10	11.82	12.27	12.39	11.61	11.61	9.39	38.92	39.05
SiTPS29	55	10.92	12.97	11.42	12.47	12.35	11.56	11.68	10.48	10.74	11.68	10.75	10.87	10.50	10.61	7.96	34.03	34.16
SvTPS24	56	10.80	12.85	11.30	12.35	12.23	11.56	11.68	10.48	10.74	11.68	10.63	10.75	10.38	10.50	7.96	33.91	34.03
SiTPS28	57	12.17	14.55	13.08	13.68	13.82	13.12	13.12	11.11	12.20	13.48	14.62	14.76	13.36	13.49	10.64	33.64	33.77
SvTPS23	58	12.17	14.55	13.08	13.55	13.68	13.12	13.12	11.11	12.20	13.48	14.62	14.76	13.36	13.49	10.64	33.38	33.51
SiTPS34	59	8.23	9.94	8.70	10.15	10.03	8.67	8.55	8.29	8.65	9.44	9.23	9.34	8.44	8.44	8.62	12.79	12.79
SvTPS26	60	8.23	9.94	8.70	10.15	10.03	8.67	8.55	8.29	8.65	9.44	9.23	9.34	8.44	8.44	8.62	12.79	12.79
SiTPS35	61	9.40	11.35	9.99	11.21	11.10	9.92	9.69	9.44	9.73	10.07	10.16	10.27	9.36	9.36	9.33	12.92	12.92
SvTPS27	62	9.23	11.15	9.81	11.01	10.90	9.74	9.52	9.27	9.55	9.89	9.98	10.09	9.19	9.19	9.16	12.69	12.69
SiTPS6	63	8.38	10.18	9.35	9.93	9.81	9.67	9.55	8.08	10.04	10.51	7.99	8.10	7.65	7.65	7.08	13.57	13.57
SvTPS6	64	8.53	10.38	9.53	10.12	10.00	9.85	9.73	8.21	10.23	10.73	8.12	8.24	7.89	7.89	7.32	13.68	13.68
SiTPS9	65	9.45	11.22	9.93	10.48	10.37	9.85	9.61	8.65	10.82	11.29	8.20	8.31	7.51	7.51	7.57	13.39	13.39
SvTPS8	66	9.45	11.22	9.93	10.48	10.37	9.73	9.50	8.54	10.71	11.29	8.20	8.31	7.51	7.51	7.57	13.39	13.39

		51	52	53	54	55	56	57	58	59	60	61	62	63	64	65	66
SiTPS19	1	12.89	12.63	11.45	11.77	11.72	11.72	13.86	13.86	9.37	9.37	9.85	9.67	9.95	10.02	10.02	10.02
SvTPS17	2	13.03	12.76	11.21	11.65	11.72	11.72	13.86	13.86	9.71	9.71	10.08	9.90	10.07	10.15	10.14	10.14
SiTPS20	3	12.63	12.37	11.45	11.65	12.08	12.08	13.73	13.73	9.37	9.37	10.08	9.90	9.59	9.65	10.26	10.26
SvTPS18	4	12.50	12.24	11.21	11.40	11.96	11.96	13.59	13.59	9.26	9.26	9.97	9.79	9.59	9.65	10.14	10.14
SiTPS21	5	13.80	13.80	11.56	12.13	12.43	12.43	13.71	13.71	9.04	9.04	9.52	9.35	8.76	8.80	9.56	9.56
SvTPS19	6	14.06	14.06	11.92	12.50	12.43	12.43	13.84	13.84	9.04	9.04	9.52	9.35	8.88	8.92	9.68	9.68
SiTPS22	7	12.72	12.58	11.02	11.71	11.23	11.23	12.89	12.89	8.98	8.98	9.09	8.93	8.67	8.71	8.64	8.64
SvTPS20	8	13.40	13.40	11.92	12.50	12.07	12.07	13.97	13.97	9.47	9.47	9.84	9.66	9.70	9.77	9.54	9.54
SiTPS16	9	13.35	13.61	11.65	12.10	12.17	12.17	13.33	13.33	8.56	8.56	9.38	9.21	9.82	9.89	9.66	9.66
SvTPS14	10	13.35	13.61	11.65	12.10	12.17	12.17	13.33	13.33	8.56	8.56	9.38	9.21	9.82	9.89	9.66	9.66
SiTPS18	11	13.22	13.48	11.65	12.10	12.29	12.29	13.59	13.59	8.56	8.56	9.50	9.33	9.82	9.89	9.89	9.89
SvTPS16	12	13.22	13.48	11.65	12.10	12.41	12.41	13.73	13.73	8.68	8.68	9.73	9.55	9.70	9.77	9.89	9.89
SiTPS27	13	13.65	13.65	11.66	12.36	12.43	12.43	13.71	13.58	8.80	8.80	9.51	9.34	9.35	9.41	9.91	9.91
SvTPS22	14	13.78	13.78	11.79	12.48	12.66	12.66	13.97	13.84	8.91	8.91	9.62	9.45	9.47	9.53	10.02	10.02
SiTPS26	15	13.39	13.65	11.54	12.24	12.90	12.90	14.10	13.97	8.69	8.69	9.62	9.45	8.99	9.03	9.32	9.32
SiTPS36	16	13.65	13.78	12.20	12.77	12.35	12.35	14.36	14.36	9.77	9.77	10.46	10.27	11.46	10.86	10.06	10.06
SvTPS28	17	13.04	13.17	11.88	12.32	11.92	11.92	13.89	13.89	9.23	9.23	9.92	9.75	10.95	10.46	9.21	9.21
SiTPS14	18	13.95	13.95	11.83	12.16	12.47	12.47	14.17	13.91	10.26	10.15	10.06	9.88	11.02	11.23	11.08	11.08
SvTPS12	19	13.95	13.95	11.83	12.16	12.47	12.47	14.17	13.91	10.26	10.15	10.06	9.88	11.02	11.23	11.08	11.08
SvTPS30	20	13.94	13.94	10.79	11.24	11.31	11.19	13.58	13.45	8.37	8.37	8.74	8.58	8.61	8.64	8.70	8.70
SiTPS15	21	15.39	15.39	11.59	12.03	12.23	12.11	14.83	14.83	9.91	9.91	10.73	10.54	11.12	11.22	10.84	10.84
SvTPS13	22	15.26	15.26	11.59	12.03	12.11	11.99	14.70	14.70	9.91	9.91	10.73	10.54	11.12	11.22	11.07	11.07
SiTPS17	23	14.13	14.13	12.24	12.70	12.24	12.12	14.45	14.32	9.59	9.59	9.73	9.55	10.67	10.88	10.15	10.15
SvTPS15	24	14.13	14.13	12.24	12.70	12.24	12.12	14.45	14.32	9.59	9.59	9.73	9.55	10.67	10.88	10.15	10.15
SiTPS11	25	14.40	14.40	11.62	12.19	12.86	12.86	13.54	13.54	10.09	10.09	10.23	10.04	10.11	10.31	9.36	9.36
SvTPS32	26	14.27	14.27	11.38	11.95	12.98	12.98	13.29	13.29	9.98	9.98	10.11	9.93	10.11	10.31	9.47	9.47
SvTPS9	27	12.88	12.88	10.21	10.75	11.47	11.47	11.82	11.82	8.54	8.54	8.68	8.52	8.44	8.59	7.87	7.87
SiTPS12	28	14.94	14.81	11.52	11.96	13.10	13.10	13.85	13.85	10.20	10.20	10.11	9.93	10.11	10.31	10.06	10.06
SvTPS10	29	15.07	14.94	11.64	12.08	13.33	13.33	13.80	13.80	10.43	10.43	10.34	10.16	10.46	10.67	10.41	10.41
SiTPS3	30	15.67	15.67	11.86	12.44	12.74	12.62	14.36	14.49	10.44	10.44	10.47	10.28	9.88	10.07	10.42	10.42
SvTPS3	31	15.67	15.67	11.86	12.44	12.86	12.74	14.49	14.61	10.44	10.44	10.47	10.28	9.88	10.07	10.42	10.42
SiTPS4	32	16.43	16.30	12.20	12.65	12.86	12.74	14.58	14.70	10.22	10.22	10.58	10.39	9.88	10.07	10.30	10.30
SvTPS4	33	16.43	16.30	12.20	12.65	12.86	12.74	14.58	14.70	10.22	10.22	10.58	10.39	9.88	10.07	10.30	10.30
SiTPS30	34	13.25	13.11	10.82	11.10	10.92	10.80	12.17	12.17	8.23	8.23	9.40	9.23	8.38	8.53	9.45	9.45
SvTPS25	35	15.63	15.50	12.79	13.09	12.97	12.85	14.55	14.55	9.94	9.94	11.35	11.15	10.18	10.38	11.22	11.22
SvTPS33	36	14.32	14.19	11.33	11.61	11.42	11.30	13.08	13.08	8.70	8.70	9.99	9.81	9.35	9.53	9.93	9.93
SiTPS38	37	14.29	14.02	12.78	12.95	12.47	12.35	13.68	13.55	10.15	10.15	11.21	11.01	9.93	10.12	10.48	10.48
SvTPS29	38	14.15	13.89	12.65	12.83	12.35	12.23	13.82	13.68	10.03	10.03	11.10	10.90	9.81	10.00	10.37	10.37
SiTPS32	39	12.88	12.75	11.60	11.63	11.56	11.56	13.12	13.12	8.67	8.67	9.92	9.74	9.67	9.85	9.85	9.73
SvTPS34	40	12.88	12.75	11.60	11.63	11.68	11.68	13.12	13.12	8.55	8.55	9.69	9.52	9.55	9.73	9.61	9.50
SiTPS31	41	11.42	11.29	10.18	10.31	10.48	10.48	11.11	11.11	8.29	8.29	9.44	9.27	8.08	8.21	8.65	8.54
SiTPS25	42	11.63	11.50	11.07	11.10	10.74	10.74	12.20	12.20	8.65	8.65	9.73	9.55	10.04	10.23	10.82	10.71
SvTPS21	43	12.53	12.40	11.79	11.82	11.68	11.68	13.48	13.48	9.44	9.44	10.07	9.89	10.51	10.73	11.29	11.29
SiTPS1	44	12.35	12.35	12.06	12.27	10.75	10.63	14.62	14.62	9.23	9.23	10.16	9.98	7.99	8.12	8.20	8.20
SvTPS1	45	12.61	12.61	12.18	12.39	10.87	10.75	14.76	14.76	9.34	9.34	10.27	10.09	8.10	8.24	8.31	8.31
SiTPS2	46	11.92	11.92	11.42	11.61	10.50	10.38	13.36	13.36	8.44	8.44	9.36	9.19	7.65	7.89	7.51	7.51
SvTPS2	47	11.92	11.92	11.42	11.61	10.61	10.50	13.49	13.49	8.44	8.44	9.36	9.19	7.65	7.89	7.51	7.51
SvTPS31	48	9.02	8.89	9.10	9.39	7.96	7.96	10.64	10.64	8.62	8.62	9.33	9.16	7.08	7.32	7.57	7.57
SiTPS8	49	49.66	50.07	38.45	38.92	34.03	33.91	33.64	33.38	12.79	12.79	12.92	12.69	13.57	13.68	13.39	13.39
SvTPS7	50	49.79	50.21	38.58	39.05	34.16	34.03	33.77	33.51	12.79	12.79	12.92	12.69	13.57	13.68	13.39	13.39
SiTPS5	51		99.31	49.87	50.97	47.10	46.73	34.11	33.74	17.95	17.95	18.53	18.20	18.56	18.63	19.54	19.42
SvTPS5	52	99.31		50.25	51.35	47.10	46.73	34.36	33.99	17.95	17.95	18.64	18.31	18.68	18.75	19.65	19.54
SiTPS13	53	49.87	50.25		95.14	46.81	46.69	35.21	35.46	19.34	19.34	19.89	19.55	20.97	21.20	20.57	20.57
SvTPS11	54	50.97	51.35	95.14		47.61	47.48	35.38	35.63	19.84	19.84	20.22	19.87	21.39	21.62	20.69	20.69
SiTPS29	55	47.10	47.10	46.81	47.61		99.13	47.73	47.85	20.22	20.22	20.27	19.91	19.61	19.81	22.58	22.58
SvTPS24	56	46.73	46.73	46.69	47.48	99.13		47.61	47.73	20.33	20.33	20.38	20.02	19.72	19.93	22.69	22.69
SiTPS28	57	34.11	34.36	35.21	35.38	47.73	47.61		98.77	14.47	14.47	14.49	14.24	14.24	14.24	14.97	14.97
SvTPS23	58	33.74	33.99	35.46	35.63	47.85	47.73	98.77		14.58	14.58	14.49	14.24	14.47	14.47	14.97	14.97
SiTPS34	59	17.95	17.95	19.34	19.84	20.22	20.33	14.47	14.58		99.28	67.02	65.90	43.49	44.71	44.16	44.04
SvTPS26	60	17.95	17.95	19.34	19.84	20.22	20.33	14.47	14.58	99.28		66.78	65.67	43.26	44.47	44.04	43.93
SiTPS35	61	18.53	18.64	19.89	20.22	20.27	20.38	14.49	14.49	67.02	66.78		98.00	42.31	43.24	43.04	42.92
SvTPS27	62	18.20	18.31	19.55	19.87	19.91	20.02	14.24	14.24	65.90	65.67	98.00		41.56	42.45	42.26	42.14
SiTPS6	63	18.56	18.68	20.97	21.39	19.61	19.72	14.24	14.47	43.49	43.26	42.31	41.56		95.71	56.93	56.93
SvTPS6	64	18.63	18.75	21.20	21.62	19.81	19.93	14.24	14.47	44.71	44.47	43.24	42.45	95.71		57.88	57.88
SiTPS9	65	19.54	19.65	20.57	20.69	22.58	22.69	14.97	14.97	44.16	44.04	43.04	42.26	56.93	57.88		99.74
SvTPS8	66	19.42	19.54	20.57	20.69	22.58	22.69	14.97	14.97	44.04	43.93	42.92	42.14	56.93	57.88	99.74	

Supplemental Fig. 3: Sequence similarity matrix of TPS candidates from S. italica and S. viridis
