# Supplementary material for: The foxtail millet (Setaria italica) terpene synthase gene family
Source: Plant J. 2020 May 3;103(2):781–800. doi: 10.1111/tpj.14771 (PMC7497057; doi:10.1111/tpj.14771)
Supplement: Supplementary file 1 — Figure S1. Protein sequence alignment of select class II diterpene synthases. Figure S2. Protein sequence alignment of select class I diterpene synthases. Figure S3. Sequence similarity matrix of terpene synthase candidates from Setaria italica and Setaria viridis. Figure S4. Mass spectra of class II diterpene synthase products identified in this study. Figure S5. Mass spectra of class I terpene synthase products identified in this study. Figure S6. Mass spectra of products resulting from co‐expression assays of SiTPS5 and SiTPS13. Figure S7. The NMR analysis of ent‐pimara‐8,15‐diene. Figure S8. The NMR analysis of syn‐pimara‐7,15‐diene. Figure S9. The NMR analysis of eudesme‐2,11‐diol. Figure S10. Functional analysis of CYP99A17 and CYP99A19. Figure S11. The NMR analysis of abietadien‐19‐ol. Figure S12. The NMR analysis of syn‐pimara‐7,15‐dien‐19‐ol. Figure S13. Gene expression analysis of characterized Setaria italica terpene synthase genes. Figure S14. Occurrence of terpene synthase and CYP99A17 products in Setaria italica. [file TPJ-103-781-s001.zip › tpj14771-sup-0005-FigS5.pdf]

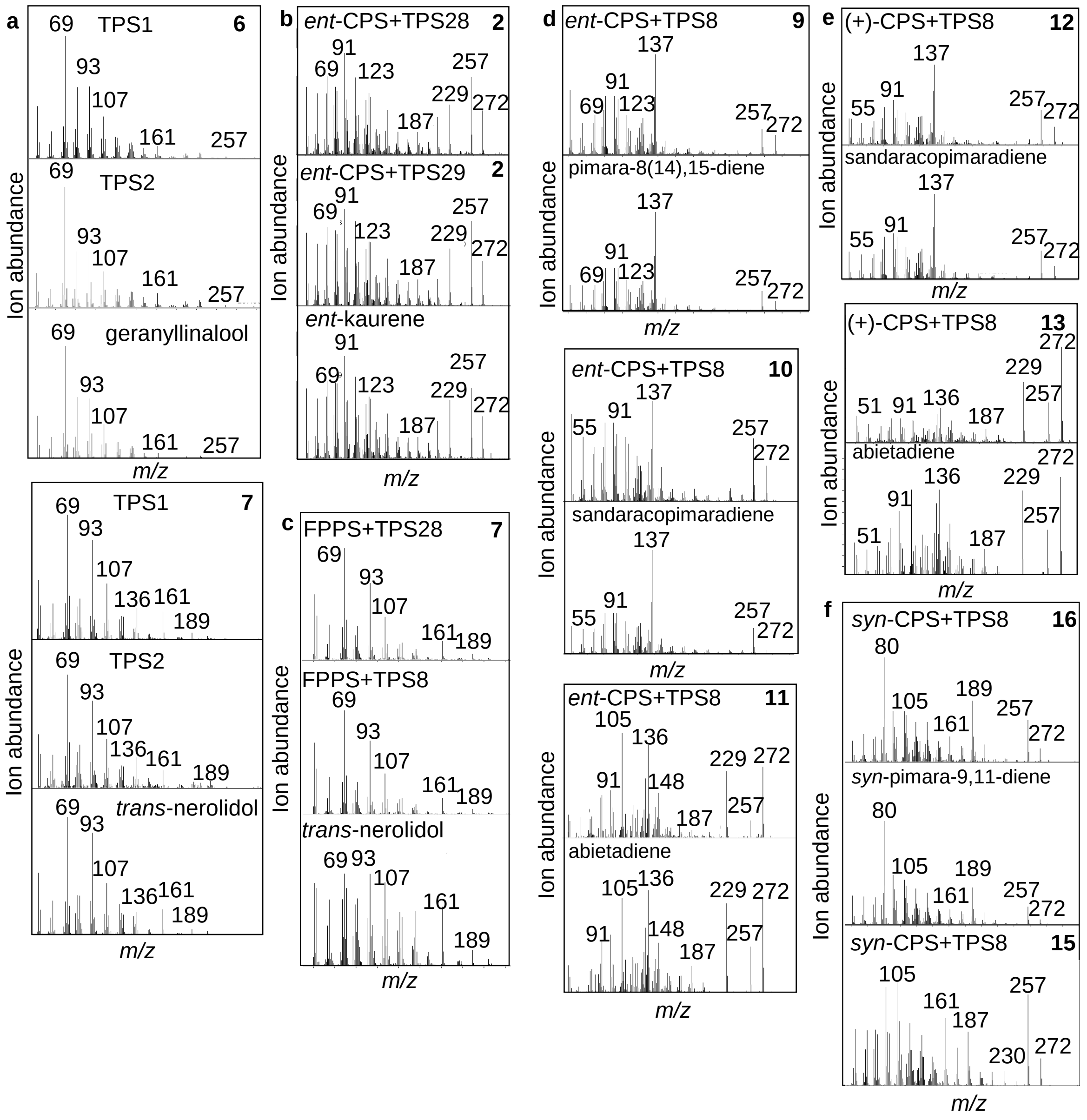

Supplemental Figure S5 : Mass spectra of all products in Figure 3 (**a**-**f**). Mass spectra for *Nicotiana benthamiana* co-expression assays of (**a**) SiTPS1 and SiTPS2 identifying both enzymes as geranyllinalool/*trans*-nerolidol synthases, (**b**) SiTPS28 or SiTPS29 with the *ent*-CPP synthase, *Zm*AN2 (Harris *et al.*, 2005), (**c-f**) *E. coli* co-expression assays of: (**c**) SiTPS28 and SiTPS8 with *E*,*E*-farnesyl pyrophosphate (FPP) produced by the maize FPP synthase, ZmFPPS (Cervantes-Cervantes *et al.*, 2006), (**d**) SiTPS8 with the *ent*-CPP synthase, *Zm*An2, (**e**) SiTPS8 with the grand fir (*Abies grandis*) abietadiene synthase variant D621A that produces (+)-CPP (Cyr *et al.*, 2007, Morrone *et al.*, 2010), (**f**) SiTPS8 with the *syn*-CPP synthase, OsCPS4 (Xu *et al.*, 2004).

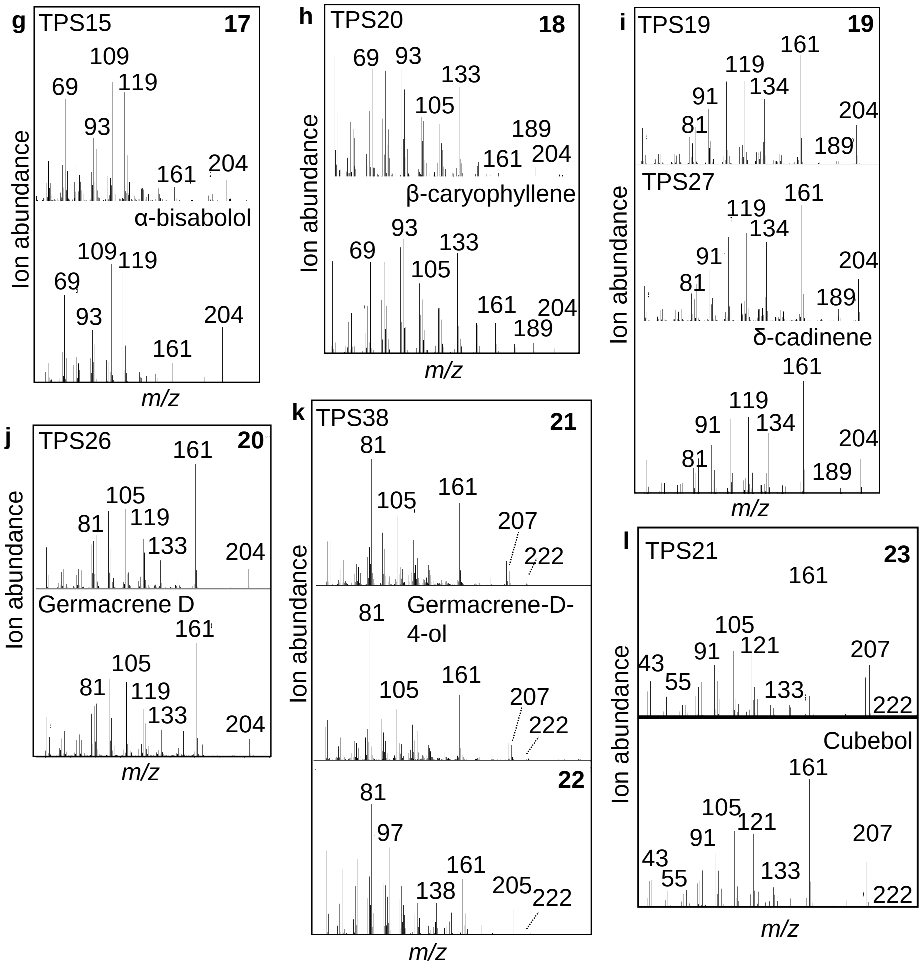

Supplemental Figure S5 : Mass spectra of all products in Figure 4 (**g-l**). Mass spectra for *Nicotiana benthamiana* co-expression of focal sesquiterpene synthases: (**g**) SiTPS15 as an α-bisabolol synthase, (**h**) SiTPS20 as a β-caryophyllene synthase, (**i**) SiTPS19 and SiTPS27 as δ-cadinene synthases, (**j**) SiTPS26 as a germacrene D synthase, (**k**) SiTPS38 as a germacrene-D-4-ol synthase, and (**l**) *E. coli* co-expression assays of the maize *E*,*E*-FPP synthase, ZmFPPS (Cervantes-Cervantes *et al.*, 2006), with SiTPS21 as a cubebol synthase. 2, *ent*-kaurene; 6, geranyllinalool; 7, *trans*-nerolidol; 8, *ent*-pimara-8(9),15-diene; 9, *ent*-pimara-8(14),15-diene; 10, *ent*-sandaracopimaradiene; 11, *ent*-abietadiene; 12, sandaracopimaradiene; 13, abietadiene; 14, *syn*-pimara-7(8),15-diene; 15, unknown diterpene; 16, *syn*-pimara-9,11-diene, 17, α-bisabolol; 18, β-caryophyllene; 19, δ-cadinene; 20, germacrene D; 21, germacrene-D-4-ol; 22, unidentified terpene product; 23, cubebol.
