# Supplementary material for: The foxtail millet (Setaria italica) terpene synthase gene family
Source: Plant J. 2020 May 3;103(2):781–800. doi: 10.1111/tpj.14771 (PMC7497057; doi:10.1111/tpj.14771)
Supplement: Supplementary file 1 — Figure S1. Protein sequence alignment of select class II diterpene synthases. Figure S2. Protein sequence alignment of select class I diterpene synthases. Figure S3. Sequence similarity matrix of terpene synthase candidates from Setaria italica and Setaria viridis. Figure S4. Mass spectra of class II diterpene synthase products identified in this study. Figure S5. Mass spectra of class I terpene synthase products identified in this study. Figure S6. Mass spectra of products resulting from co‐expression assays of SiTPS5 and SiTPS13. Figure S7. The NMR analysis of ent‐pimara‐8,15‐diene. Figure S8. The NMR analysis of syn‐pimara‐7,15‐diene. Figure S9. The NMR analysis of eudesme‐2,11‐diol. Figure S10. Functional analysis of CYP99A17 and CYP99A19. Figure S11. The NMR analysis of abietadien‐19‐ol. Figure S12. The NMR analysis of syn‐pimara‐7,15‐dien‐19‐ol. Figure S13. Gene expression analysis of characterized Setaria italica terpene synthase genes. Figure S14. Occurrence of terpene synthase and CYP99A17 products in Setaria italica. [file TPJ-103-781-s001.zip › tpj14771-sup-0006-FigS6.pdf]

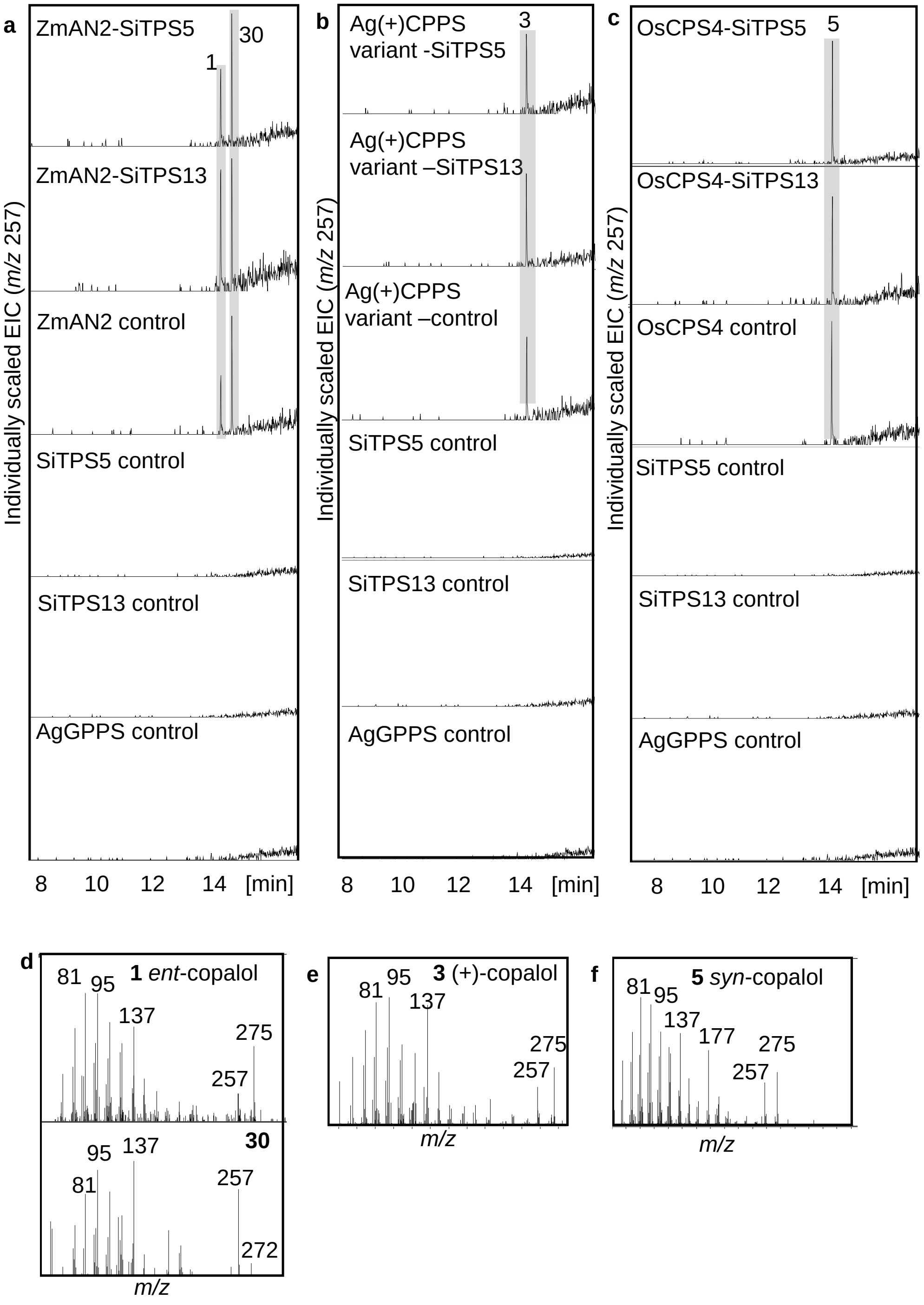

Supplemental Fig. S6: Functional characterization of the *S. italica* class I diTPS candidates TPS5 and TP13 in *E. coli*. (**a**) GC-MS traces of *E. coli* co-expression assays of SiTPS5 and SiTPS13 with the *ent*-CPP synthase, *Zm*AN2 (Harris *et al.*, 2005). (**b**) Co-expression of SiTPS5 or SiTPS13 with the the grand fir (*Abies grandis*) abietadiene synthase variant D621A that produces (+)-CPP (Cyr *et al.*, 2007; Morrone *et al.*, 2010). (**c**) Co-expression of SiTPS5 or SiTPS13 with the *syn*-CPP synthase, OsCPS4 (Xu *et al.*, 2004). (**d-f**) Mass spectra for all detected TPS products. 1, *ent*-copalol (dephosphorylated *ent*-CPP); 3, (+)-copalol (dephosphorylated *(+)*-CPP); 5, *syn*-copalol (dephosphorylated *syn*-CPP); 26-28, unidentified dephosphorylated derivatives of FPP; 29, farnesol (main dephosphorylated product of FPP); 30, unidentified dephosphorylated derivative of *ent*-CPP as reported previously (Pelot *et al.*, 2018).

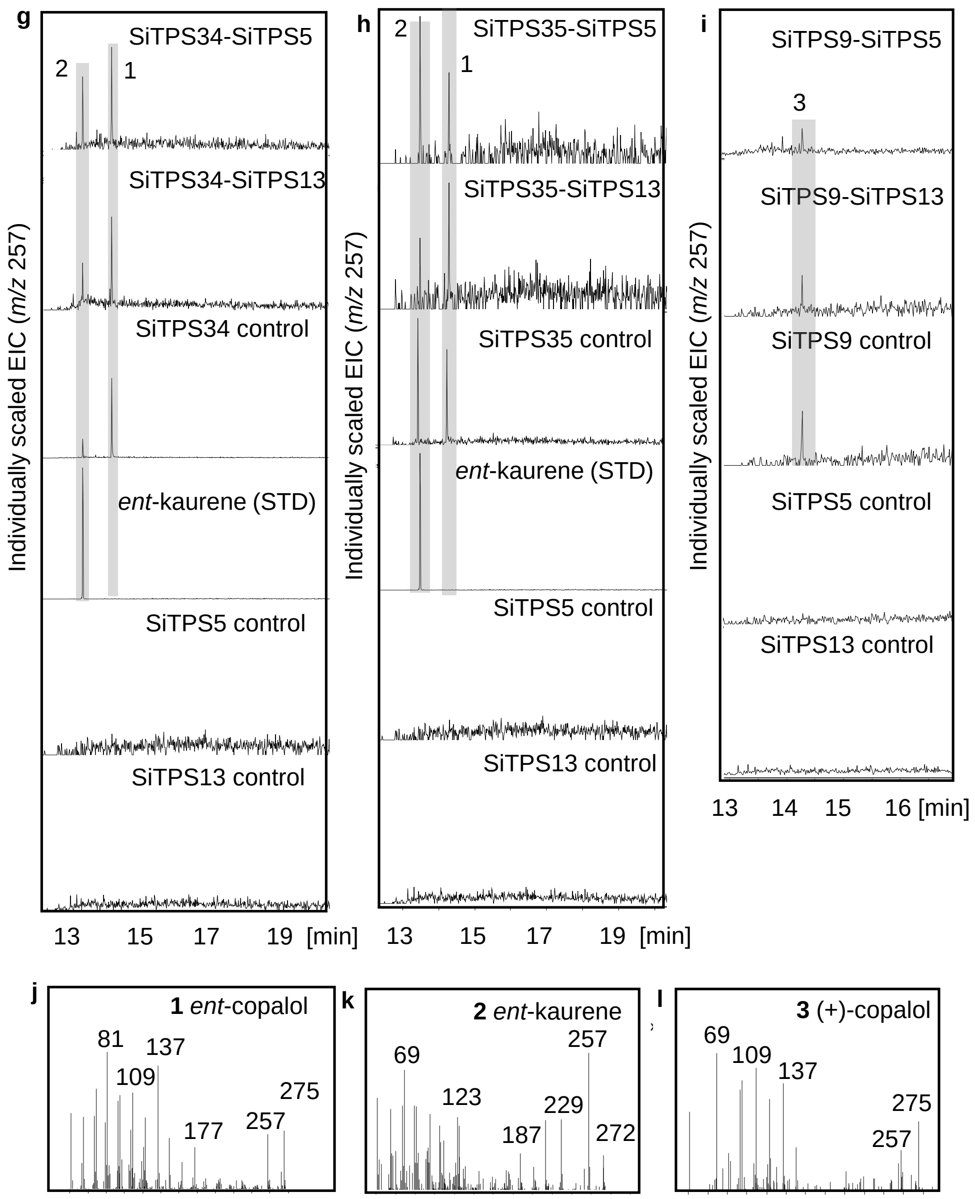

Supplemental Fig. S6: Functional characterization of the *S. italica* class I diTPS candidates TPS5 and TP13 in *N. benthamiana*. (**g**) GC-MS traces of co-expression assays of SiTPS5 and SiTPS13 with the *ent*-CPP synthase, SiTPS34. (**h**) GC-MS traces of co-expression assays of SiTPS5 and SiTPS13 with the *ent*-CPP synthase, SiTPS35. (**i**) GC-MS traces of co-expression assays of SiTPS5 and SiTPS13 with the (+)-CPP synthase, SiTPS9. (**j-l**) Mass spectra for all detected TPS products. 1, *ent*-copalol (dephosphorylated *ent*-CPP); 2, *ent*-kaurene; 3, (+)-copalol (dephosphorylated *(+)*-CPP).
