# Supplementary material for: The foxtail millet (Setaria italica) terpene synthase gene family
Source: Plant J. 2020 May 3;103(2):781–800. doi: 10.1111/tpj.14771 (PMC7497057; doi:10.1111/tpj.14771)

Supplemental Fig. 9: NMR analysis of eudesme-2,11-diol (product 25) formed through the coupled reaction of TPS25 and Zea mays FPP synthase.

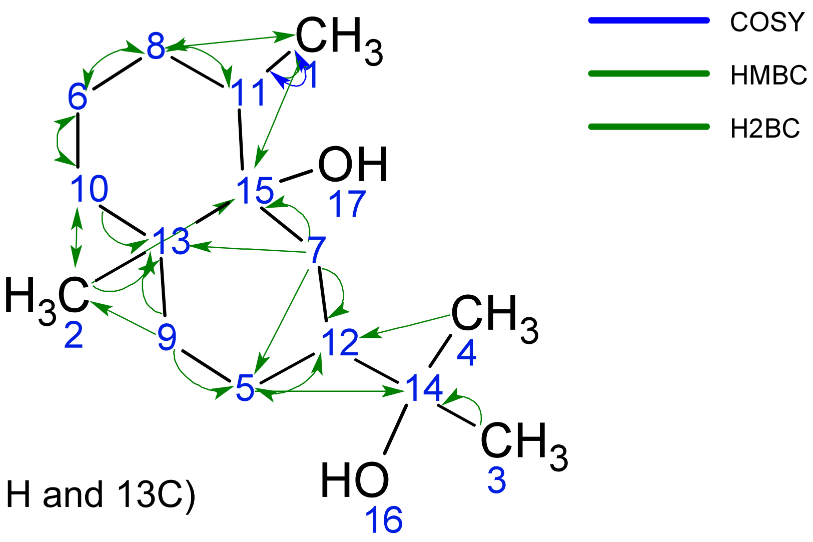

A) 1D NMR (1H and 13C)

#	Atom#	C Label	C Shift	XHn	H Label	H Shift	C Calc Shift (Neural Net)	H Calc Shift (Neural Net)	H Multiplicity	COSY	H HMBC	C HMBC
1	1	M03	14.840	CH3	H 1	0.856	17.359	0.954	d (6.81)	11	8, 11	8, 15
2	5	M05	20.141	CH2	H 15	1.770	23.411	1.550	m		9, 12, 9, 7	12, 14
3	5	M05	20.141	CH2	H 17	1.889	23.411	1.550	m		9, 12, 9, 7	
4	6	M06	20.553	CH2	H 12	1.657	21.184	1.474	m		8, 10	8, 10
5	6	M06	20.553	CH2	H 8	1.459	21.184	1.474	m		8, 10	
6	2	M04	20.747	CH3	H 4	1.060	22.838	0.988	s		10, 9	10, 13, 15
7	7	M01	29.497	CH2	H 18	2.050	34.865	1.703	br d (15.16)			5, 13, 12, 15
8	7	M01	29.497	CH2	H 10	1.483	34.865	1.703	m			
9	3	M11	29.643	CH3	H 6	1.265	27.930	1.219	s			14
10	4	M12	29.701	CH3	H 5	1.262	27.930	1.219	br s			12
11	8	M02	30.573	CH2	H 7	1.294	31.982	1.407	dd (13.11, 5.11)		1, 6, 11	1, 6, 11
12	8	M02	30.573	CH2	H 9	1.474	31.982	1.407	br d (2.04)		1, 6, 11	
13	11	M08	34.172	CH	H 14	1.745	36.641	1.850	m	1	8	1, 8
14	9	M09	34.215	CH2	H 16	1.844	35.493	1.439	m			5, 2, 13
15	9	M09	34.215	CH2	H 2	0.954	35.493	1.439	m			5
16	10	M10	34.693	CH2	H 3	1.008	34.103	1.381	m		2, 6	
17	10	M10	34.693	CH2	H 11	1.535	34.103	1.381	td (13.54, 13.54, 4.09)		2, 6	6, 2, 13
18	13	M15	36.855	C			37.068				2, 10, 9, 7	
19	12	M07	40.786	CH	H 13	1.666	45.660	2.208	m		4, 5, 7	5

A) 1D NMR continued

#	Atom#	C Label	C Shift	XHn	H Label	H Shift	C Calc Shift (Neural Net)	H Calc Shift (Neural Net)	H Multiplicity	COSY	H HMBC	C HMBC
20	14	M14	73.090	C			72.759				3, 5	
21	15	M13	74.257	C			73.956				1, 2, 7	

B) 1H NMR

Acquisition Time (sec)	2.9360	***Comment***	1H-45 sec scan		
D	0.0002	***D1***	2	***DE***	18
DS	2	***Date***	21 Sep 2017 19:18:24		
Date Stamp	21 Sep 2017 19:18:24				
File Name	\\169.237.229.248\share$\ACDLabs\Zerbe\Prema\092117-TPS25-BEST\1\PDATA\1\1r				
Frequency (MHz)	800.1500	***GB***	0	***INSTRUM***	<spect>
LB	0.1	***NS***	8	***Nucleus***	1H
Number of Transients	8	***Origin***	spect	***Original Points Count***	32768
Owner	pkarunan	***PC***	1		
PROBHD	<5 mm CPTCI 1H-13C/15N/D Z-GRD Z107231/0001 >				
PULPROG	<zg30>	***Points Count***	65536	***Pulse Sequence***	zg30
Receiver Gain	18.00	***SF***	800.15	***SFO1***	800.15400075
SI	65536	***SSB***	0	***SW(cyclical) (Hz)***	11160.71
SWH	11160.7142857143			***Solvent***	CHLOROFORM-d
Spectrum Offset (Hz)	3990.0298	***Spectrum Type***	standard	***Sweep Width (Hz)***	11160.54
TD	65536	***TD0***	1	***TE***	303
Temperature (degree C)	30.000	***UNC1***	<1H>	***WDW***	1

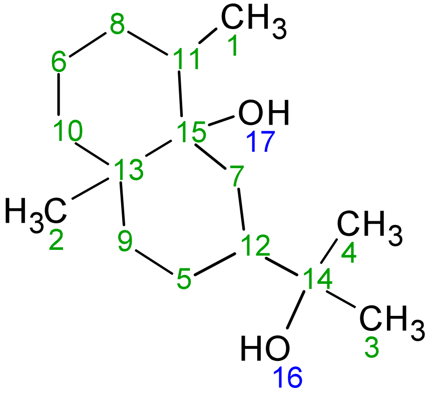

^{1}H NMR (800 MHz, CHLOROFORM-*d*) δ ppm 0.86 (d, *J*=6.81 Hz, 3 H) 0.94 - 0.97 (m, 1 H) 0.99 - 1.03 (m, 1 H) 1.06 (s, 3 H) 1.26 (br s, 4 H) 1.27 (s, 2 H) 1.29 (dd, *J*=13.11, 5.11 Hz, 1 H) 1.44 - 1.48 (m, 1 H) 1.46 (br d, *J*=2.04 Hz, 1 H) 1.46 - 1.51 (m, 1 H) 1.54 (td, *J*=13.54, 4.09 Hz, 1 H) 1.63 - 1.68 (m, 1 H) 1.65 - 1.68 (m, 1 H) 1.72 - 1.76 (m, 1 H) 1.75 - 1.80 (m, 1 H) 1.82 - 1.87 (m, 1 H) 1.87 - 1.91 (m, 1 H) 2.05 (br d, *J*=15.16 Hz, 1 H)

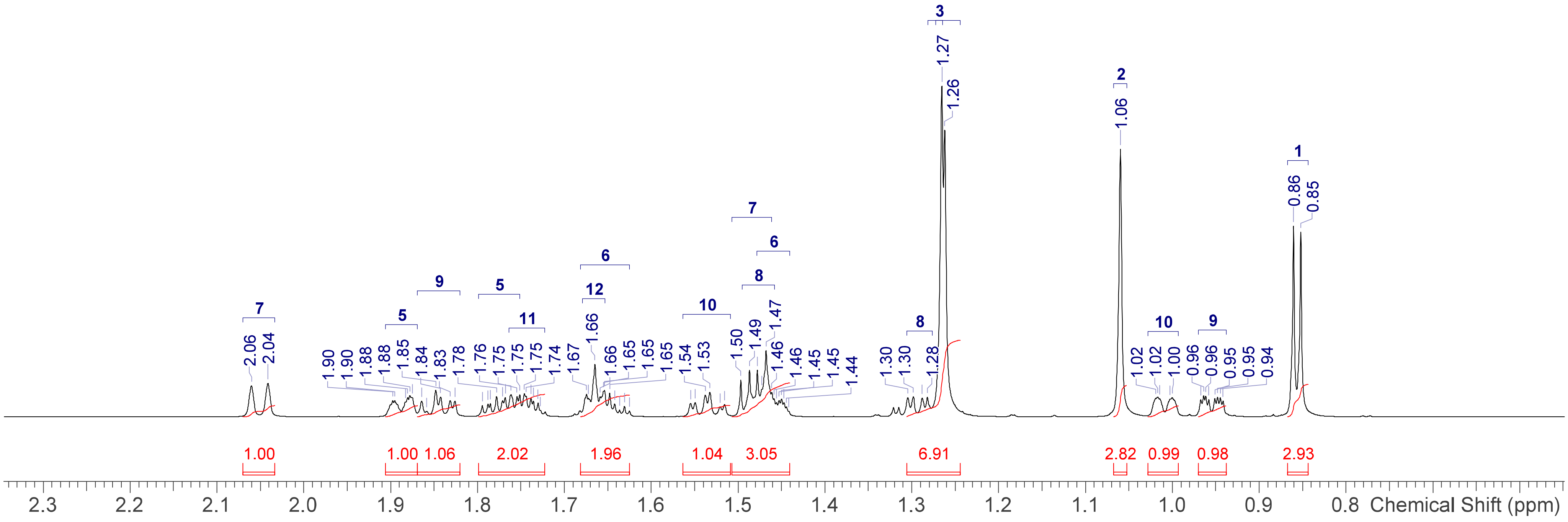

C) 13C NMR

Acquisition Time (sec)	0.6816	***Comment***	13C-20 min
D	1.5	***D1***	1.5
DE	18.85148	***DS***	4
Date	21 Sep 2017 19:45:06		
Date Stamp	21 Sep 2017 19:45:06		
File Name	\\169.237.229.248\share$\ACDLabs\Zerbe\Prema\092117-TPS25-BEST\2\PDATA\1 \1r		
Frequency (MHz)	201.1979	***GB***	0
INSTRUM	<spect>	***LB***	0.3
NS	512	***Nucleus***	13C
Number of Transients	512	***Origin***	spect
Original Points Count	32768	***Owner***	pkarunan
PC	1.4		
PROBHD	<5 mm CPTCI 1H-13C/15N/D Z-GRD Z107231/0001 >		
PULPROG	<zgpg30>	***Points Count***	65536
Pulse Sequence	zgpg30	***Receiver Gain***	645.00
SF	201.19787753		
SFO1	201.220009296528		
SI	65536	***SSB***	0
SW(cyclical) (Hz)	48076.92	***SWH***	48076.9230769231
Solvent	CHLOROFORM-d		
Spectrum Offset (Hz)	22130.7383	***Spectrum Type***	standard
Sweep Width (Hz)	48076.19	***TD***	65536
TD0	1	***TE***	303
Temperature (degree C)	30.000	***UNC1***	<13C>
WDW	1		

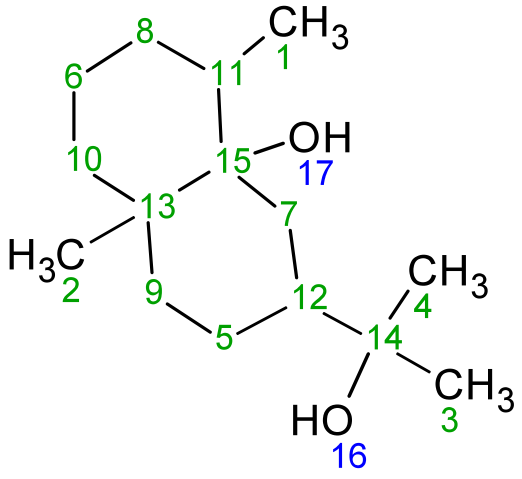

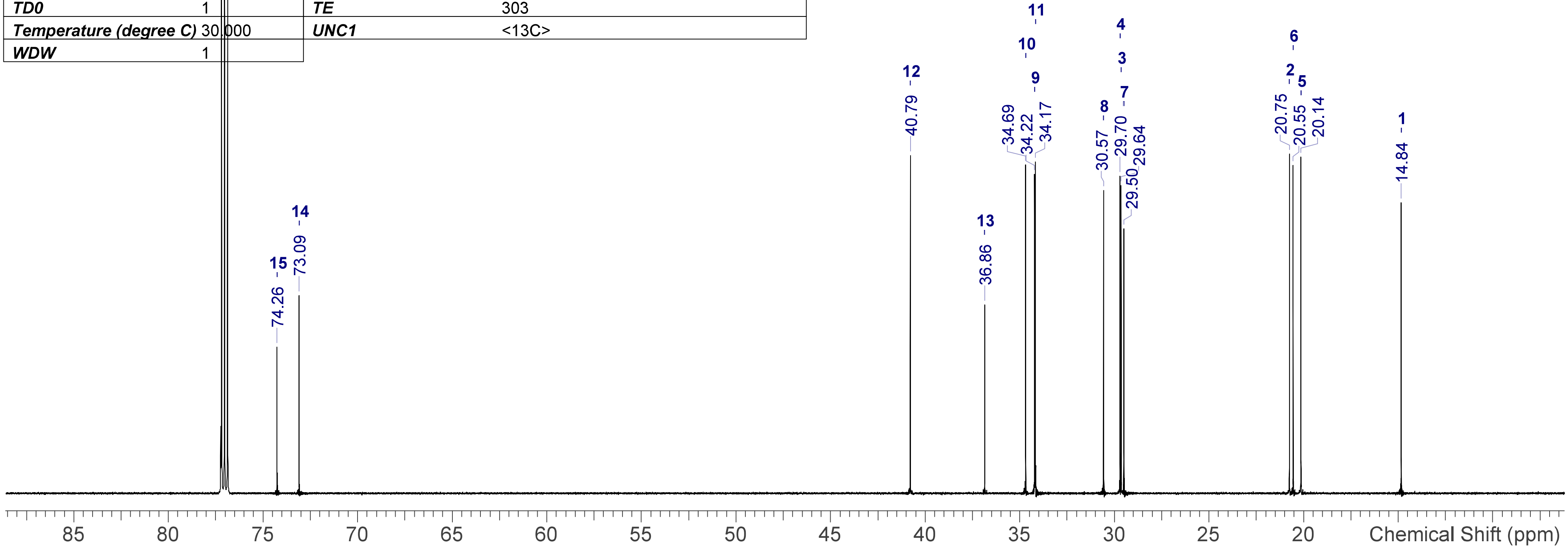

D) 2D NMR HSQC

Acquisition Time (sec)	(0.1507, 0.0201)	***Comment***	2D-HSQC
Constant (Hz)	145.0	***Date***	21 Sep 2017 23:16:16
File Name	\\169.237.229.248\share$\ACDLabs\Zerbe\Prema\092117-TPS25-BEST\4\ser		
Frequency (MHz)	(800.1500, 201.1979)		
Nucleus	(1H, 13C)	***Number of Transients***	8
Origin	spect	***Original Points Count***	(1024, 400)
Owner	pkarunan	***Points Count***	(1024, 1024)
Pulse Sequence	hsqcedetgpsp.3	***Solvent***	CHLOROFORM-d
Spectrum Type	HSQC	***Sweep Width (Hz)***	(6786.84, 19900.87)
Temperature (degree C)	30.000	***Title***	2D-HSQC

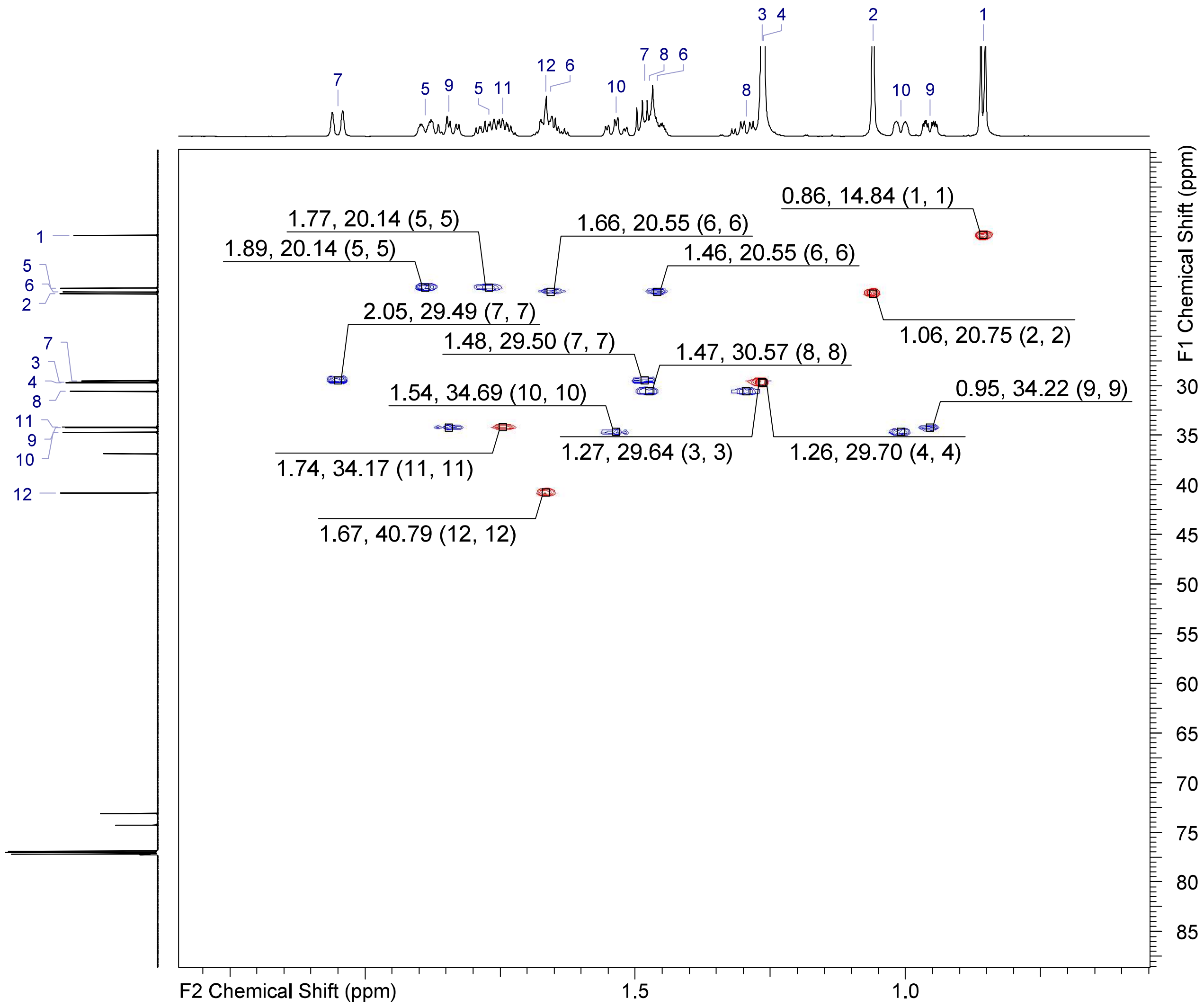

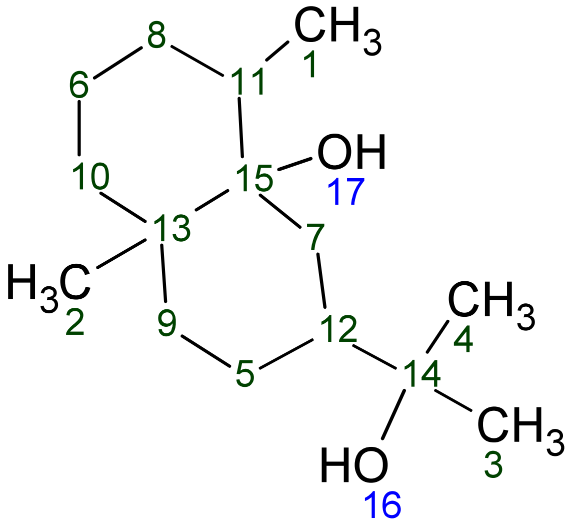

E) 2D NMR COSY

Acquisition Time (sec)	(0.1507, 0.0377)	***Comment***	2D-COSY
Date	21 Sep 2017 21:19:24		
File Name	\\169.237.229.248\share$\ACDLabs\Zerbe\Prema\092117-TPS25-BEST\3\ser		
Frequency (MHz)	(800.1500, 800.1500)		
Nucleus	(1H, 1H)	***Number of Transients***	8
Origin	spect	***Original Points Count***	(1024, 256)
Owner	pkarunan	***Points Count***	(1024, 2048)
Pulse Sequence	cosygpmfppqf	***Solvent***	CHLOROFORM-d
Spectrum Type	COSY	***Sweep Width (Hz)***	(6786.84, 6790.16)
Temperature (degree C)	30.000	***Title***	2D-COSY

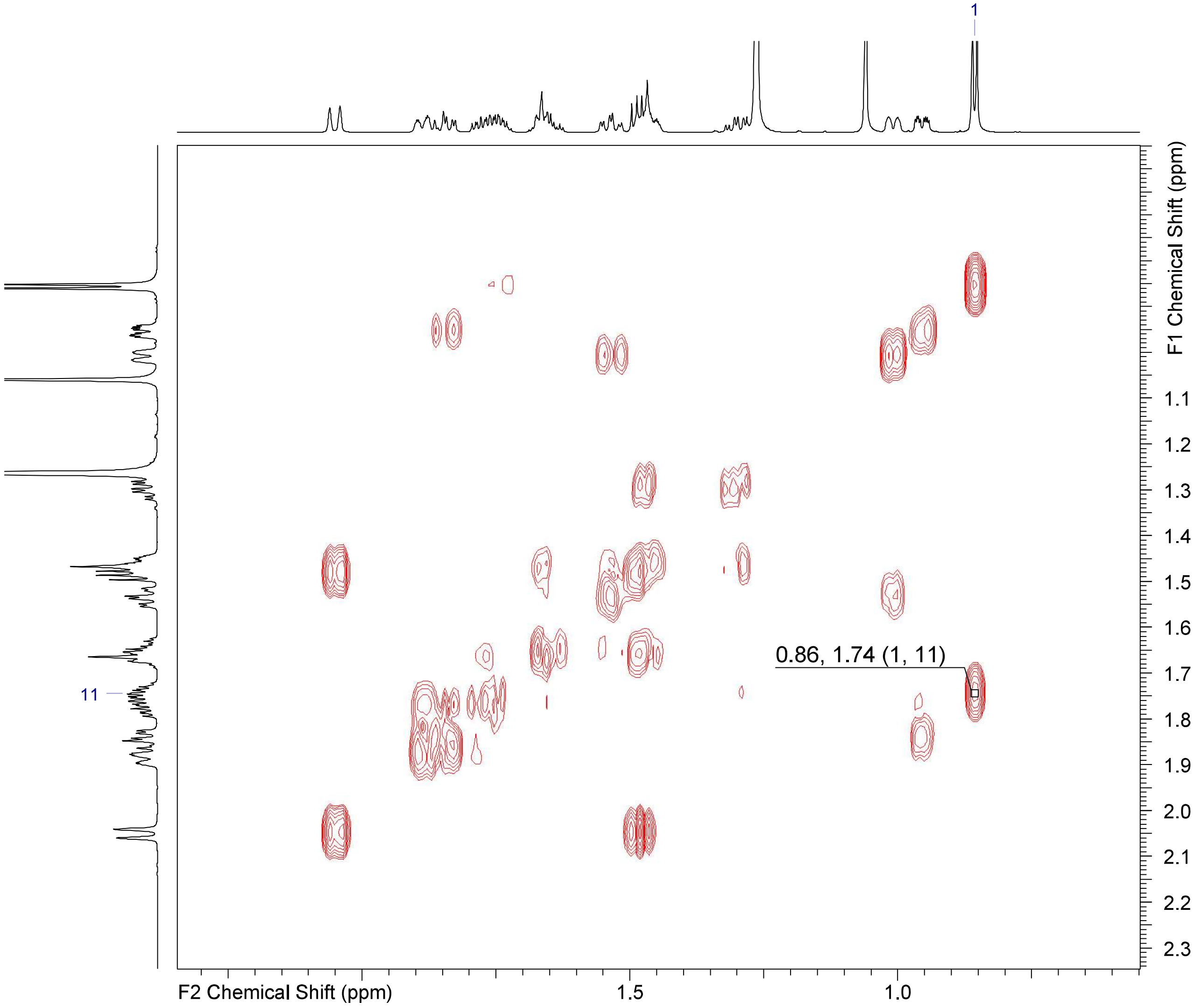

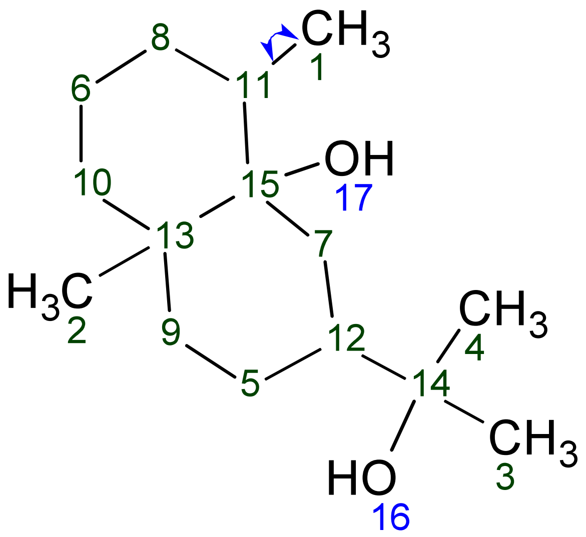

F) 2D NMR HMBC

Acquisition Time (sec)	(0.3015, 0.0257)	***Comment***	2D-HMBC
Constant (Hz)	8.0	***Date***	22 Sep 2017 03:34:42
File Name	\\169.237.229.248\share$\ACDLabs\Zerbe\Prema\092117-TPS25-BEST\5\ser		
Frequency (MHz)	(800.1500, 201.1979)		
Nucleus	(1H, 13C)	***Number of Transients***	16
Origin	spect	***Original Points Count***	(2048, 512)
Owner	pkarunan	***Points Count***	(2048, 2048)
Pulse Sequence	hmbcetgpl3nd	***Solvent***	CHLOROFORM-d
Spectrum Type	HMBC	***Sweep Width (Hz)***	(6790.16, 19910.59)
Temperature (degree C)	30.000	***Title***	2D-HMBC

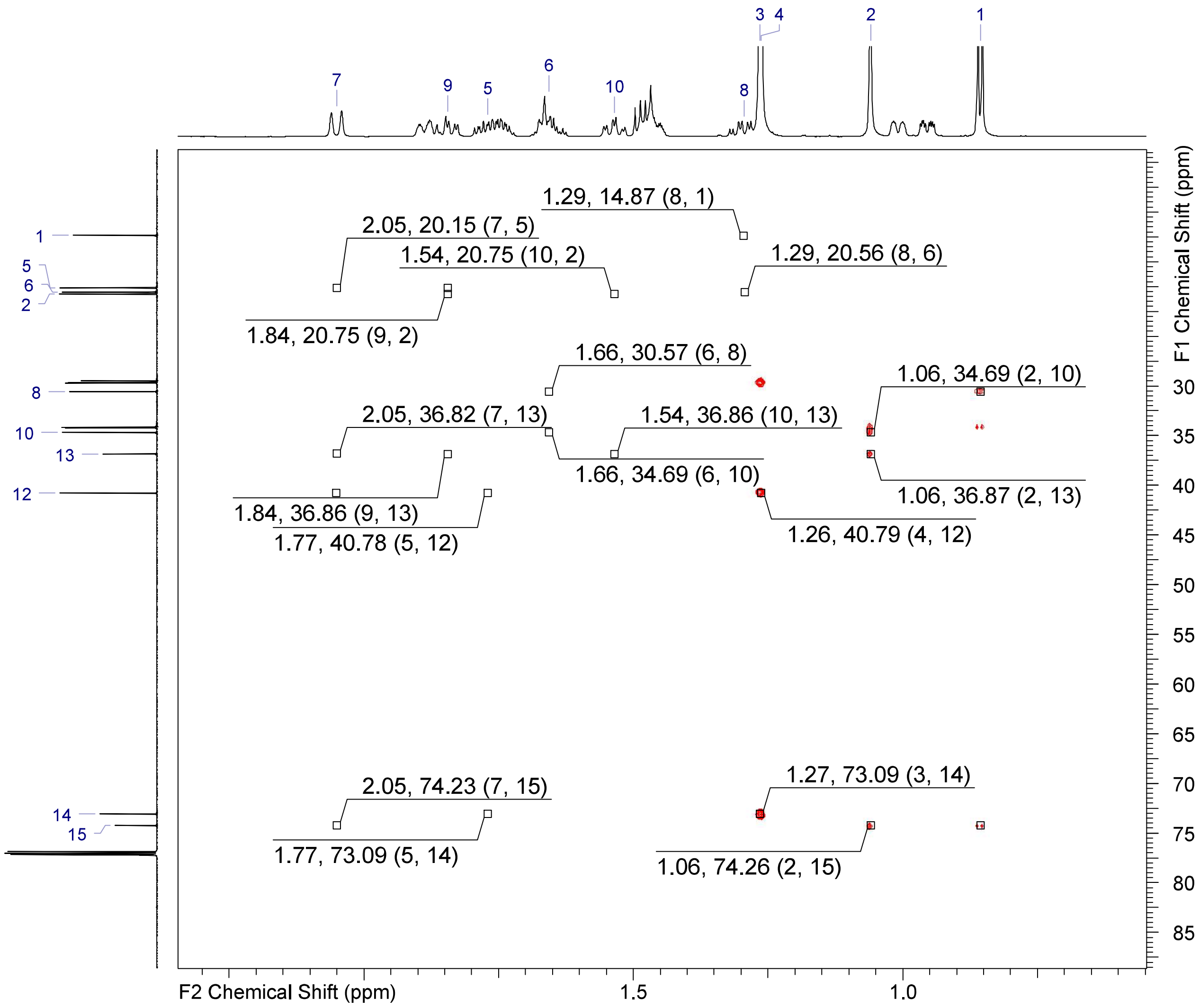

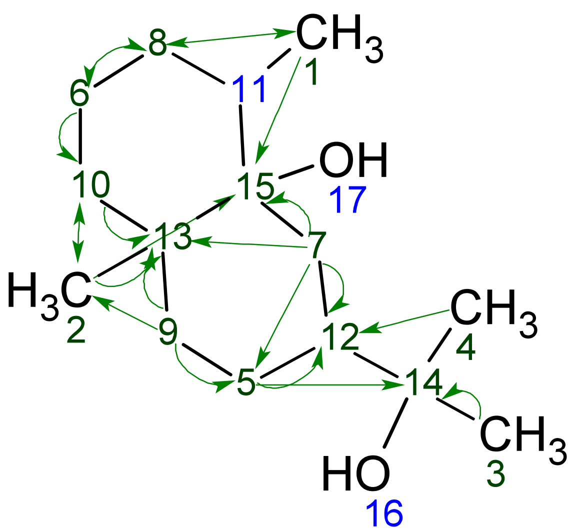

G) 2D NMR H2BC

Acquisition Time (sec)	(0.3015, 0.0129)	***Comment***	2D-H2BC
Date	22 Sep 2017 04:39:16		
File Name	\\169.237.229.248\share$\ACDLabs\Zerbe\Prema\092117-TPS25-BEST\6\ser		
Frequency (MHz)	(800.1500, 201.1979)		
Nucleus	(1H, 13C)	***Number of Transients***	8
Origin	spect	***Original Points Count***	(2048, 256)
Owner	pkarunan	***Points Count***	(2048, 1024)
Pulse Sequence	h2bcetgpl3	***Solvent***	CHLOROFORM-d
Spectrum Type	H2BC	***Sweep Width (Hz)***	(6790.16, 19900.87)
Temperature (degree C)	30.000	***Title***	2D-H2BC

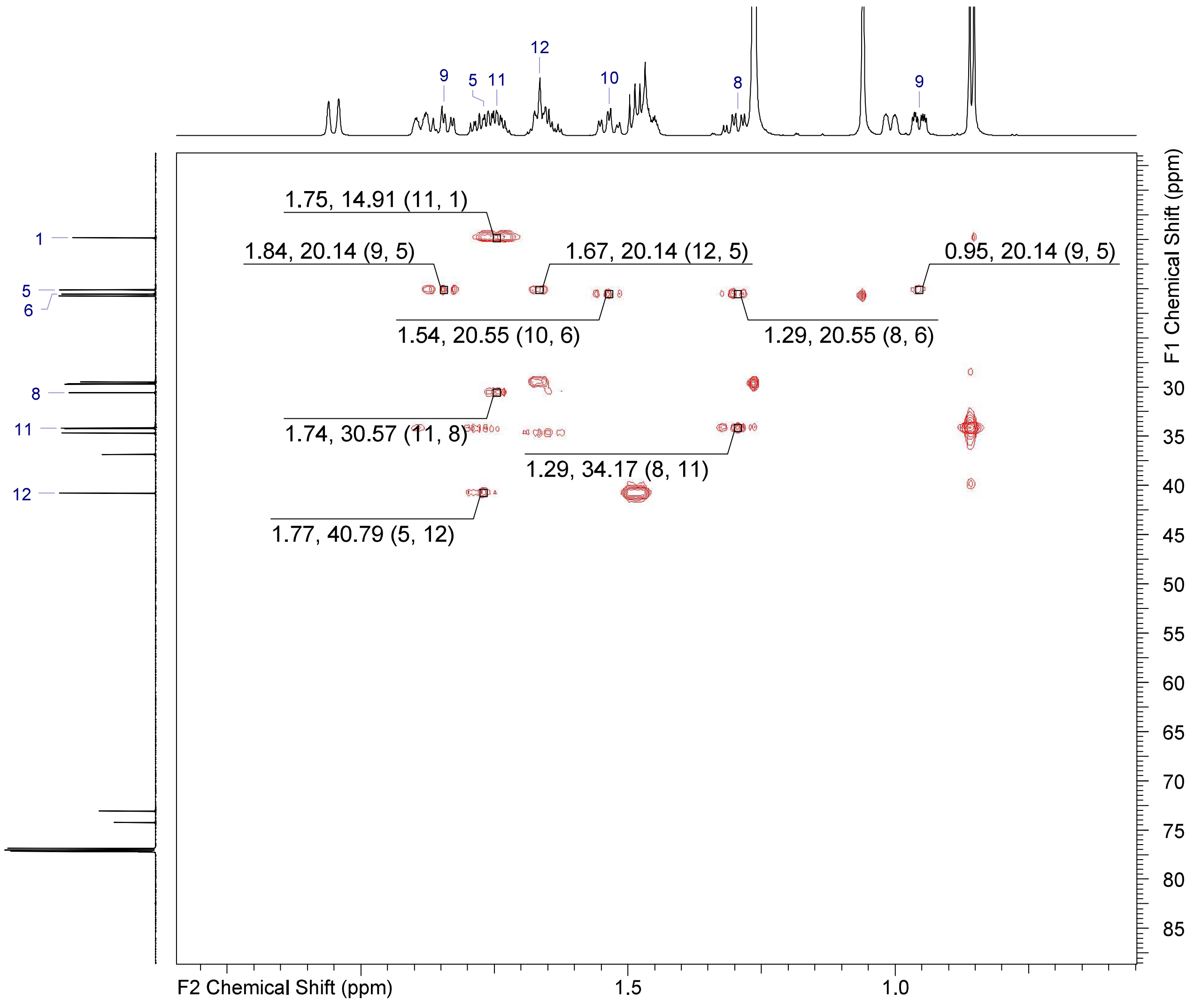

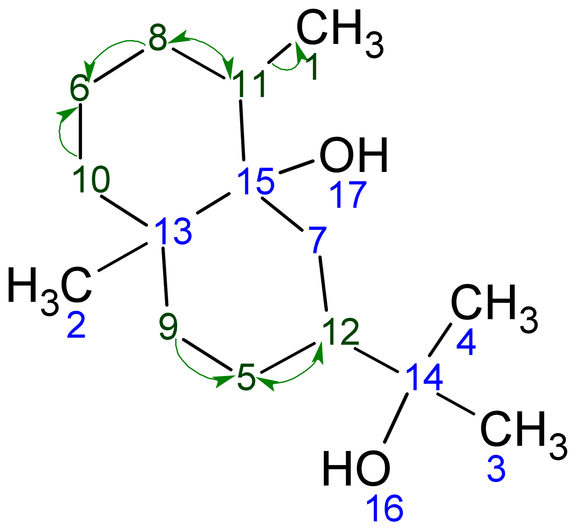

Supplement: Supplementary file 1 — Figure S1. Protein sequence alignment of select class II diterpene synthases. Figure S2. Protein sequence alignment of select class I diterpene synthases. Figure S3. Sequence similarity matrix of terpene synthase candidates from Setaria italica and Setaria viridis. Figure S4. Mass spectra of class II diterpene synthase products identified in this study. Figure S5. Mass spectra of class I terpene synthase products identified in this study. Figure S6. Mass spectra of products resulting from co‐expression assays of SiTPS5 and SiTPS13. Figure S7. The NMR analysis of ent‐pimara‐8,15‐diene. Figure S8. The NMR analysis of syn‐pimara‐7,15‐diene. Figure S9. The NMR analysis of eudesme‐2,11‐diol. Figure S10. Functional analysis of CYP99A17 and CYP99A19. Figure S11. The NMR analysis of abietadien‐19‐ol. Figure S12. The NMR analysis of syn‐pimara‐7,15‐dien‐19‐ol. Figure S13. Gene expression analysis of characterized Setaria italica terpene synthase genes. Figure S14. Occurrence of terpene synthase and CYP99A17 products in Setaria italica. [file TPJ-103-781-s001.zip › tpj14771-sup-0009-FigS9.pdf]
