# Supplementary material for: The foxtail millet (Setaria italica) terpene synthase gene family
Source: Plant J. 2020 May 3;103(2):781–800. doi: 10.1111/tpj.14771 (PMC7497057; doi:10.1111/tpj.14771)
Supplement: Supplementary file 1 — Figure S1. Protein sequence alignment of select class II diterpene synthases. Figure S2. Protein sequence alignment of select class I diterpene synthases. Figure S3. Sequence similarity matrix of terpene synthase candidates from Setaria italica and Setaria viridis. Figure S4. Mass spectra of class II diterpene synthase products identified in this study. Figure S5. Mass spectra of class I terpene synthase products identified in this study. Figure S6. Mass spectra of products resulting from co‐expression assays of SiTPS5 and SiTPS13. Figure S7. The NMR analysis of ent‐pimara‐8,15‐diene. Figure S8. The NMR analysis of syn‐pimara‐7,15‐diene. Figure S9. The NMR analysis of eudesme‐2,11‐diol. Figure S10. Functional analysis of CYP99A17 and CYP99A19. Figure S11. The NMR analysis of abietadien‐19‐ol. Figure S12. The NMR analysis of syn‐pimara‐7,15‐dien‐19‐ol. Figure S13. Gene expression analysis of characterized Setaria italica terpene synthase genes. Figure S14. Occurrence of terpene synthase and CYP99A17 products in Setaria italica. [file TPJ-103-781-s001.zip › tpj14771-sup-0010-FigS10.pdf]

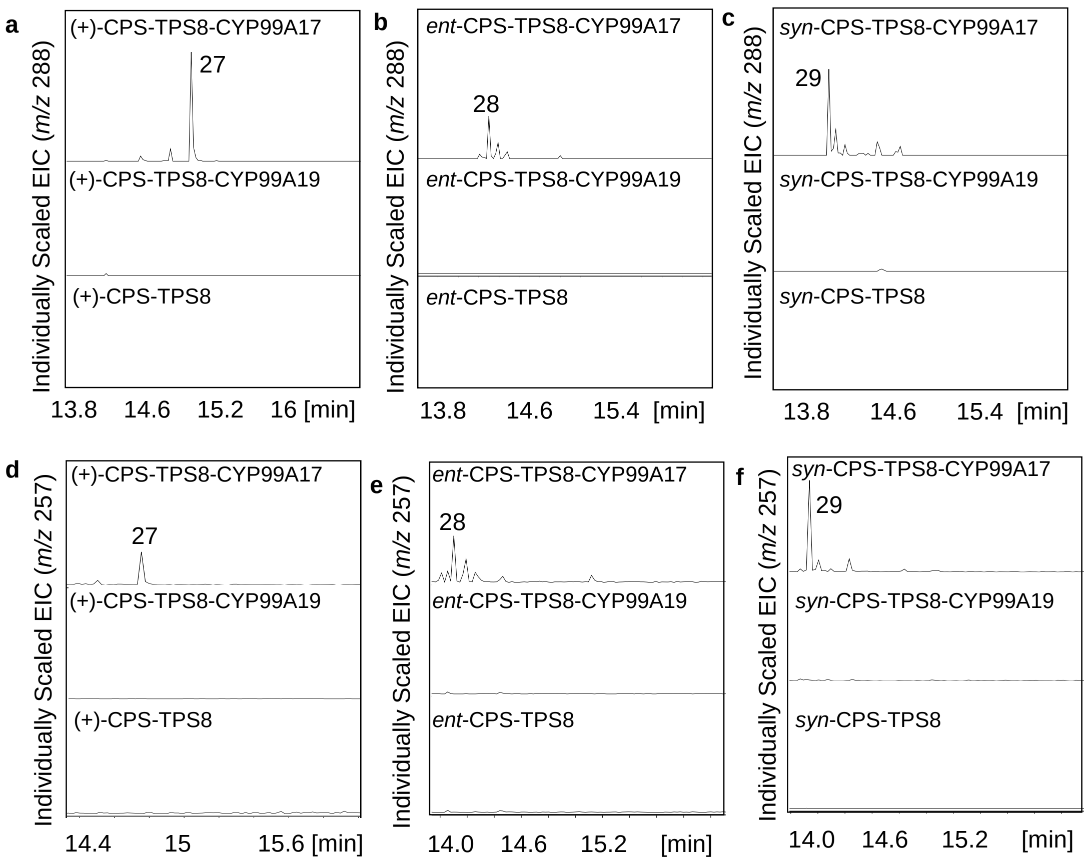

Supplemental Fig. 10: GC-MS traces of *E. coli* co-expression assays of *S. italica* CYP99A17 and CYP99A19. Extracted ion chromatograms for m/z 288 when (+)-CDP (**a**), ent-CDP (**b**), or syn-CDP (**c**) is used as substrate. Extracted ion chromatograms for m/z 257 when (+)-CDP (**d**), ent-CDP (**e**), or syn-CDP (**f**) is used as substrate. 27 as abietadiene-19-ol as verified by NMR analysis, 28 as unidentified, and 29 as syn-pimara-7(8),15-dien-19-ol as verified by NMR analysis.
