# Supplementary material for: The foxtail millet (Setaria italica) terpene synthase gene family
Source: Plant J. 2020 May 3;103(2):781–800. doi: 10.1111/tpj.14771 (PMC7497057; doi:10.1111/tpj.14771)
Supplement: Supplementary file 1 — Figure S1. Protein sequence alignment of select class II diterpene synthases. Figure S2. Protein sequence alignment of select class I diterpene synthases. Figure S3. Sequence similarity matrix of terpene synthase candidates from Setaria italica and Setaria viridis. Figure S4. Mass spectra of class II diterpene synthase products identified in this study. Figure S5. Mass spectra of class I terpene synthase products identified in this study. Figure S6. Mass spectra of products resulting from co‐expression assays of SiTPS5 and SiTPS13. Figure S7. The NMR analysis of ent‐pimara‐8,15‐diene. Figure S8. The NMR analysis of syn‐pimara‐7,15‐diene. Figure S9. The NMR analysis of eudesme‐2,11‐diol. Figure S10. Functional analysis of CYP99A17 and CYP99A19. Figure S11. The NMR analysis of abietadien‐19‐ol. Figure S12. The NMR analysis of syn‐pimara‐7,15‐dien‐19‐ol. Figure S13. Gene expression analysis of characterized Setaria italica terpene synthase genes. Figure S14. Occurrence of terpene synthase and CYP99A17 products in Setaria italica. [file TPJ-103-781-s001.zip › tpj14771-sup-0014-FigS14.pdf]

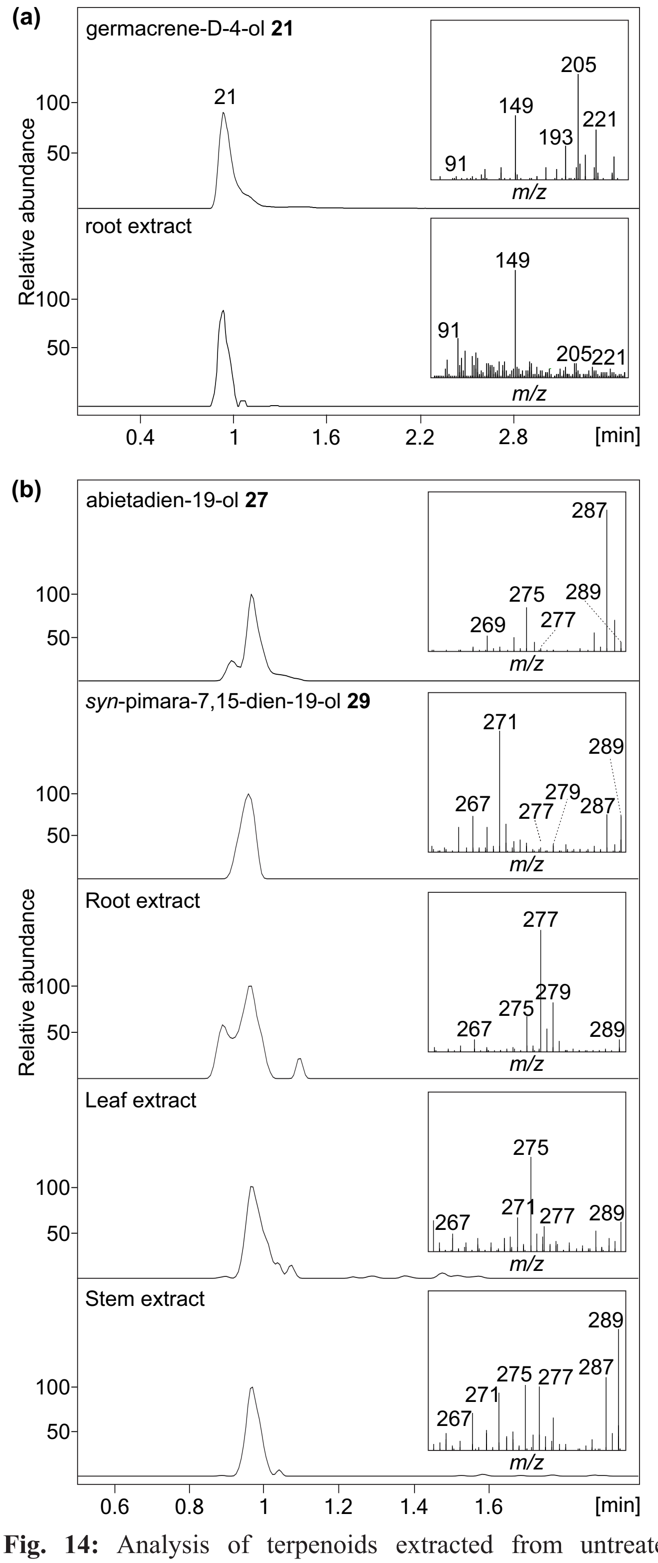

Supplemental Fig. 14: Analysis of terpenoids extracted from untreated and stress-treated samples used in gene expression studies of four-week old *S. italica* plants under $CuSO_4$ (leaves), drought stress (root), UV irradiation (leaves), and mycoprotein (stems) using UPLC-HILIC-MS/MS analysis as compared to the enzyme-produced standards. Chromatograms are representative of three biological replicates with terpenes detected in both stressed and non-stressed samples. (**a**) Traces represent combined extracted ion chromatograms (EIC) (*m*/*z* 205.1942-205.1958). In-source decay of the $[M]^+$ *m*/*z* 205.195076 germacrene-D-4-ol parent ion yields the corresponding dehydrated protonated adduct $[M\text{-}H_2O\text{+}H]^+$ *m*/*z* 222.1984 occurring at a retention time of 0.93 min. (**b**) Traces represent combined extracted ion chromatograms (EIC) (*m*/*z* 289.2511-289.2539). In-source decay of the $[M]^+$ *m*/*z* 288.2453 of abietadien-19-ol and *syn*-pimara-7,15-dien-19-ol parent ion yields the corresponding protonated adducts $[M\text{+}H]^+$ *m*/*z* 289.2525 and the protonated dehydrated adducts $[M\text{-}H_2O\text{+}H]^+$ *m*/*z* 271.2419 occurring in roots, leaves and stems at a retention time of 0.96 min.
